# Comparative safety signal prioritization of CDK4/6 inhibitors in breast cancer: a FAERS pharmacovigilance, machine learning, and clinical contextualization study

**DOI:** 10.3389/fphar.2026.1889178

**Published:** 2026-08-19

**Authors:** Linlin Fan, Shaochun Liu, Yuhan Tang, Xiaoxi Han, Zhaoqi Zhang, Chang Su, Zhiqi Sui, Wenhui Zhao

**Affiliations:** Department of Medical Oncology, Harbin Medical University Cancer Hospital, Harbin Medical University, Harbin, Heilongjiang, China

**Keywords:** breast cancer, cyclin-dependent kinase 4 and 6 inhibitors, food and drug administration adverse event reporting system, machine learning, pharmacovigilance

## Abstract

**Background:**

Cyclin-dependent kinase 4 and 6 (CDK4/6) inhibitors are standard endocrine-based treatments for hormone receptor-positive (HR-positive), HER2-negative breast cancer, but drug-specific adverse event reporting patterns differ among agents. This study prioritized adverse events of special interest (AESIs) for palbociclib, abemaciclib, and ribociclib using the Food and Drug Administration Adverse Event Reporting System (FAERS), exploratory machine learning (ML), and institutional clinical contextualization.

**Methods:**

Primary suspect FAERS reports from 2015Q1 to 2025Q4 were analyzed at the report level. Reports were mapped to eight AESI domains and assessed using disproportionality measures, intraclass-adjusted reporting odds, reported time to onset, and exploratory ML signal prioritization. ML models were developed using data from 2015Q1 to 2022Q4 and temporally validated using data from 2023Q1 to 2025Q4. Consecutive patients treated at a Chinese tertiary cancer center between 6 January 2023 and 29 December 2024 were reviewed.

**Results:**

FAERS included 115,971 class-wide primary suspect reports and 67,314 reports with breast cancer recorded as the indication. The three-agent ML dataset included 115,771 reports, with 76,581 for model development and 39,190 for temporal validation. Palbociclib was characterized mainly by hematologic reporting; abemaciclib by gastrointestinal, thrombotic, pulmonary, and renal- or creatinine-related prioritization; and ribociclib by QT interval and hepatobiliary prioritization. The institutional cohort included 82 patients and contextualized these AESIs through treatment-emergent events, grade 3 or higher toxicity, dose modification, and temporal clinical consequence markers.

**Conclusion:**

The three agents showed distinct report-level safety reporting profiles. These findings prioritize drug-specific AESIs for pharmacovigilance review and safety hypothesis generation; however, incidence, causal effects, and patient-level risk estimation require denominator-based clinical datasets.

## Introduction

Cyclin-dependent kinase 4 and 6 (CDK4/6) inhibitors are a standard component of endocrine-based treatment for hormone receptor-positive (HR-positive) and human epidermal growth factor receptor 2-negative (HER2-negative) breast cancer. Pivotal phase 3 programs established the efficacy of palbociclib in the PALOMA studies (Finn et al., 2016; Cristofanilli et al., 2016), ribociclib in the MONALEESA program and the early breast cancer setting (Hortobagyi et al., 2016; Tripathy et al., 2018; Slamon et al., 2018; Hortobagyi et al., 2022; Slamon et al., 2024), and abemaciclib in MONARCH and monarchE studies (Sledge et al., 2017; Goetz et al., 2017; Johnston et al., 2020; Rastogi et al., 2024). Regulatory approvals have extended palbociclib, ribociclib, and abemaciclib across metastatic and adjuvant treatment settings (Beaver et al., 2015; Shah et al., 2018; Royce et al., 2022; Gao et al., 2025). This expanding exposure makes drug-specific safety prioritization, dose management, and monitoring burden central to agent selection.

The three main breast cancer CDK4/6 inhibitors have clinically recognizable but overlapping safety profiles. Palbociclib is dominated by hematologic toxicity in PALOMA safety analyses (Diéras et al., 2019; Verma et al., 2016), abemaciclib has a prominent gastrointestinal toxicity profile in MONARCH clinical evidence and meta-analytic syntheses plus thrombotic signals in post-marketing literature (Sledge et al., 2017; Goetz et al., 2017; Onesti and Jerusalem, 2021; Hua et al., 2024; Gao et al., 2023), and ribociclib has a stronger QT and hepatobiliary monitoring profile (Yan et al., 2024; Tang et al., 2025). Umbrella review evidence supports the same broad pattern while showing that adverse-event trade-offs vary by endpoint, grade, and trial context (Pu et al., 2024).

Post-marketing evidence provides complementary information by integrating signals across multiple safety domains in the Food and Drug Administration Adverse Event Reporting System (FAERS). Recent FAERS analyses have described class-wide CDK4/6 signals, abemaciclib-specific signals, and three-agent comparative patterns (Peng et al., 2025; Shu et al., 2023; Lin et al., 2024). Methodological work in pharmacovigilance shows how machine learning (ML) methods, timing features, and multivariable prioritization can organize individual case safety reports for review (Al-Azzawi et al., 2023; Battini et al., 2024; Gosselt et al., 2022). Few CDK4/6 inhibitor studies have combined multidomain adverse events of special interest (AESI) mapping, intraclass-adjusted reporting odds, reported time to onset (TTO), temporal ML validation, model interpretability, and institutional clinical contextualization within a single analysis.

Therefore, this study integrated FAERS primary suspect reports from 2015Q1 to 2025Q4 with an exploratory FAERS-based ML signal prioritization analysis and a single-center Chinese institutional cohort. Broad CDK4/6 agent retrieval was used for class completeness, comparative modeling was restricted to clinically comparable and sufficiently captured agents, AESIs were mapped across organ domains, intraclass adjusted reporting odds and reported TTO were estimated, temporal report-level ML prioritization was assessed, and selected prioritized signals were examined for clinically observable management correlates in routine care.

## Methods

### Study design and data sources

Three analytical components were integrated: FAERS pharmacovigilance signal detection, exploratory FAERS-based ML signal prioritization, and a single-center retrospective institutional clinical contextualization cohort. FAERS was analyzed as a report-level spontaneous-reporting system, and disproportionality results were interpreted as signal-prioritization outputs, consistent with evidence on underreporting, spontaneous-report interpretation, and disproportionality methods (Hazell and Shakir, 2006; García-Abeijon et al., 2023; Kant et al., 2022; van Puijenbroek et al., 2002; Cutroneo et al., 2023). TRIPOD + AI and PROBAST + AI-informed transparency principles were followed for model reporting and appraisal (Collins et al., 2024; Moons et al., 2025). Detailed data-processing rules, AESI dictionaries, model specifications, and institutional variable derivation are provided in the supplementary methods.

### FAERS data source and agent eligibility

Quarterly public FAERS files for 2015Q1 to 2025Q4 were downloaded from the FDA website, and primary suspect individual case safety reports were constructed. Each report was used as the unit of analysis. Deleted or duplicate records were removed according to FDA report identifiers, and CDK4/6 inhibitor names and synonyms were standardized before report construction. Palbociclib, abemaciclib, ribociclib, dalpiciclib, and trilaciclib were screened for class completeness. Primary comparative analyses were restricted *a priori* to palbociclib, abemaciclib, and ribociclib because these clinically comparable breast cancer endocrine therapy CDK4/6 inhibitors had sufficient FAERS capture. Agent eligibility, analysis roles, reporter geography, and region-stratified AESI reporting patterns are detailed in Supplementary Table S1.

### AESI definitions and preferred term cleaning

Eight AESI domains were prespecified: hematologic toxicity, gastrointestinal toxicity, hepatobiliary toxicity, QT and cardiac toxicity, venous thromboembolism and thrombotic events, interstitial lung disease and pneumonitis, infection and febrile events, and renal or creatinine abnormalities. Medical Dictionary for Regulatory Activities (MedDRA) preferred terms were mapped to these domains with prespecified AESI dictionaries. Disease progression, metastasis, therapeutic response decreased, condition aggravated, off-label use, product issue, drug ineffective, and indication-like terms were excluded from the main preferred term panel. Death/fatal information was analyzed at the report outcome/seriousness level. For preferred-term signal screening, at least three drug-event reports and concordant evidence across prespecified disproportionality thresholds were required. Preferred-term cleaning sensitivity and death/fatal outcome-handling analyses are described in the supplementary methods.

### Disproportionality, intraclass reporting odds, and reported TTO

Reporting odds ratio (ROR), proportional reporting ratio (PRR), information component (IC), and empirical Bayes geometric mean (EBGM) were estimated as established spontaneous-reporting signal-detection measures (van Puijenbroek et al., 2002; Cutroneo et al., 2023) and were interpreted as reporting disproportionality within a denominator-free system. For each AESI, intraclass-adjusted reporting-odds models were fitted within CDK4/6 inhibitor reports, with palbociclib as the reference and adjustment for age, sex, report year, reporter country or region, and reporter type when models converged. Intraclass outputs and abemaciclib-versus-ribociclib contrasts are provided in Supplementary Table S2. Reported TTO was calculated for reports that contained plausible therapy start and event dates; TTO summaries and availability information are provided in Supplementary Table S3.

### Exploratory ML signal prioritization

The ML dataset was restricted to primary suspect reports for palbociclib, abemaciclib, and ribociclib. Reports from 2015Q1 to 2022Q4 were used for model development, and those from 2023Q1 to 2025Q4 were used for temporal validation. For each AESI, a post-report elastic-net logistic regression model was specified, with baseline-only elastic-net and random-forest models used as sensitivity analyses. Preferred terms were not used as predictors, and serious outcomes, death, hospitalization, and institutional variables were reserved for consequence and supplementary analyses outside the AESI predictor set. Available report-level proxy features included drug identity, report year, reporter country or region, reporter type, concomitant medication burden, endocrine therapy recording, and reported TTO where available; FAERS lacks standardized fields for cancer stage, comorbidity, treatment line, laboratory trajectories, and prior chemotherapy. Report-level prioritization was evaluated with area under the receiver operating characteristic curve (AUROC), area under the precision-recall curve (AUPRC), AUPRC lift, calibration, threshold metrics, top-k enrichment, and incremental value beyond drug identity and reporting-context variables, following pharmacovigilance ML and individual case safety report prioritization work (Al-Azzawi et al., 2023; Battini et al., 2024; Gosselt et al., 2022; Dauner et al., 2023). Model outputs, sensitivity analyses, incremental-value summaries, threshold metrics, grouped feature contributions, and interpretation boundaries are provided in Supplementary Tables S4–S7.

### Institutional cohort and clinical contextualization

Consecutive patients with breast cancer at a tertiary cancer center in China who initiated palbociclib, abemaciclib, ribociclib, or another CDK4/6 inhibitor from 6 January 2023 to 29 December 2024 were screened, and safety follow-up was censored on 21 March 2026. The institutional cohort included 82 patients with documented CDK4/6 exposure and follow-up safety information. This cohort was used for exploratory clinical contextualization from a Chinese tertiary practice setting. Safety was ascertained through the center’s routine safety-monitoring workflow, including clinician-documented toxicity review, serial hematology and chemistry testing recorded in the medical record, imaging when available, dose-modification records, hospitalization records, and survival status. Clinically meaningful treatment-emergent AESI was defined by new onset or worsening after CDK4/6 inhibitor initiation plus event-specific clinical evidence, including laboratory abnormality, explicit clinical documentation, or toxicity-related dose interruption, reduction, or discontinuation. All-cause hospitalization and all-cause death occurring after a documented AESI were analyzed separately as temporal clinical-consequence markers. Institutional recoding, clinical consequences, endocrine and concomitant-medication context, cross-source concordance, and variable availability are detailed in Supplementary Tables S8–S12.

## Results

### Study derivation and analytic datasets

**FIGURE 1:**
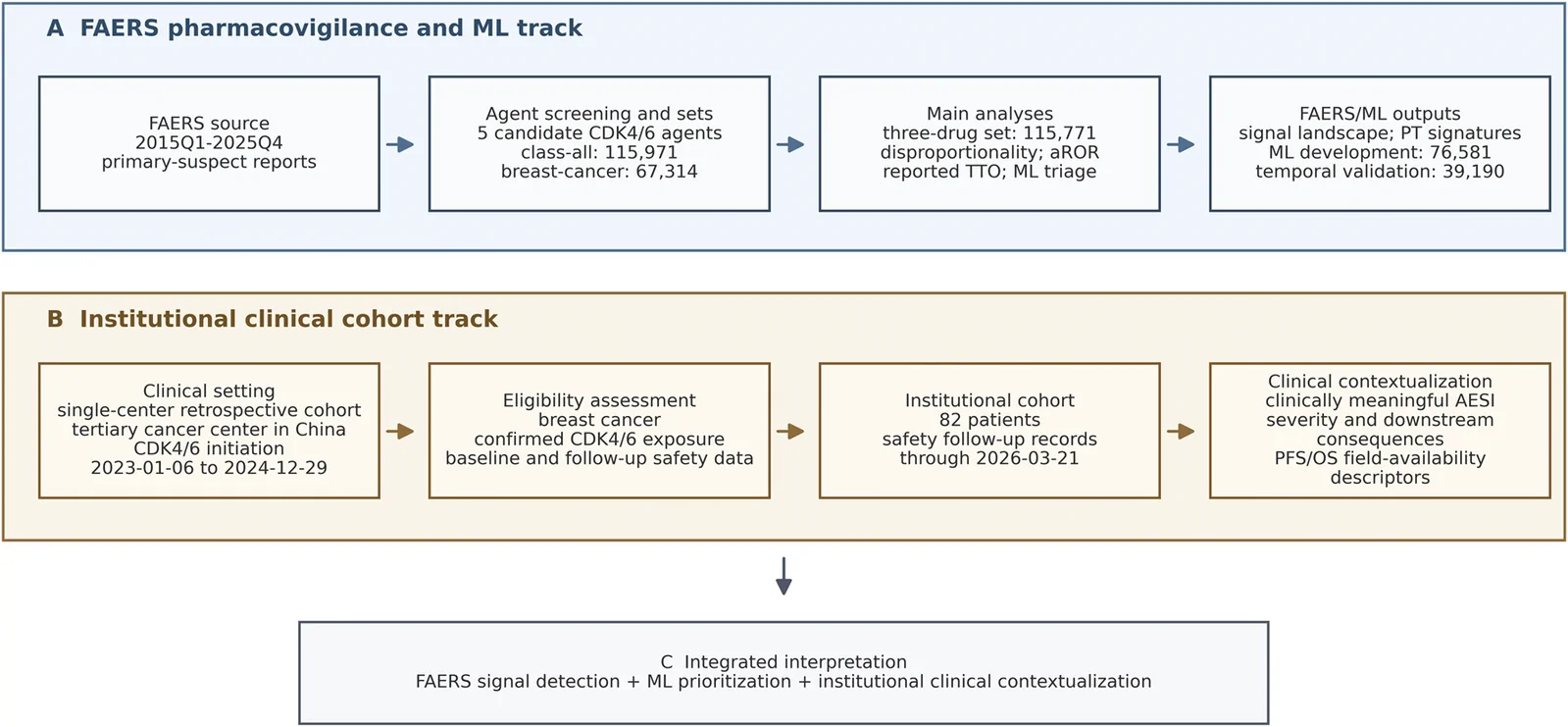
Study design and analytic overview. **(A)** Shows the FAERS pharmacovigilance and ML component from primary suspect reports through broad agent screening, prespecified three-drug comparative analyses, AESI mapping, reported TTO analysis, and temporal ML validation. **(B)** Shows the single-center institutional cohort, including CDK4/6 inhibitor initiation from 6 January 2023 to 29 December 2024 and safety follow-up censored on 21 March 2026. Panel B also notes PFS/OS field-availability descriptors, which document clinical-data availability for exploratory cohort context outside the FAERS/ML signal-definition layer. **(C)** Shows integrated interpretation across FAERS signal detection, ML prioritization, and institutional clinical contextualization. aROR, adjusted reporting odds ratio; AESI, adverse events of special interest; FAERS, Food and Drug Administration Adverse Event Reporting System; ML, machine learning; OS, overall survival; PFS, progression-free survival; PT, preferred term; TTO, time to onset.

Figure 1 summarizes the dual-source study design and analytic derivation. The FAERS class-wide dataset contained 115,971 all-indication primary suspect CDK4/6 inhibitor reports, including 67,314 reports with breast cancer recorded as the indication. The three-drug comparative dataset contained 115,771 primary suspect reports, including 80,682 for palbociclib, 15,330 for abemaciclib, and 19,759 for ribociclib. Within this three-drug dataset, 67,306 reports had breast cancer recorded as the indication because eight breast cancer indication reports belonged to non-main agents retained for class eligibility screening. Table 1 summarizes report characteristics for the three FAERS comparative agents, including age group, sex, reporter region, reporter type, indication recording, concomitant endocrine therapy, time-to-onset availability, and AESI reporting domains. Reporter region distribution and region-stratified AESI summaries are provided in Supplementary Table S1. The model development set comprised 76,581 reports, and the temporal validation set comprised 39,190 reports. The institutional contextualization cohort included 82 patients, of whom 44 received palbociclib, 21 abemaciclib, six ribociclib, and 11 other CDK4/6 inhibitors.

**TABLE 1:** Characteristics of FAERS primary suspect reports for palbociclib, abemaciclib, and ribociclib.

Characteristic	Overall (N = 115,771)	Palbociclib (N = 80,682)	Abemaciclib (N = 15,330)	Ribociclib (N = 19,759)
Patient and report characteristics				
Age group				
Younger than 50 years	10,805 (9.3)	7,074 (8.8)	1,650 (10.8)	2,081 (10.5)
50–64 years	30,054 (26.0)	23,053 (28.6)	3,615 (23.6)	3,386 (17.1)
65–74 years	26,382 (22.8)	21,827 (27.1)	2,223 (14.5)	2,332 (11.8)
75 years or older	20,864 (18.0)	18,093 (22.4)	1,365 (8.9)	1,406 (7.1)
Missing	27,666 (23.9)	10,635 (13.2)	6,477 (42.3)	10,554 (53.4)
Sex				
Female	106,927 (92.4)	75,677 (93.8)	13,484 (88.0)	17,766 (89.9)
Male	2,404 (2.1)	1,835 (2.3)	250 (1.6)	319 (1.6)
Missing or unknown	6,440 (5.6)	3,170 (3.9)	1,596 (10.4)	1,674 (8.5)
Reporter region				
United States	84,244 (72.8)	65,583 (81.3)	11,303 (73.7)	7,358 (37.2)
Europe	7,584 (6.6)	2,779 (3.4)	1,272 (8.3)	3,533 (17.9)
Asia-Pacific	7,780 (6.7)	4,666 (5.8)	1,696 (11.1)	1,418 (7.2)
Other or missing	16,163 (14.0)	7,654 (9.5)	1,059 (6.9)	7,450 (37.7)
Reporter type				
Consumer	54,387 (47.0)	34,911 (43.3)	9,602 (62.6)	9,874 (50.0)
Physician	17,964 (15.5)	10,897 (13.5)	1,730 (11.3)	5,337 (27.0)
Other health professional	19,778 (17.1)	15,453 (19.2)	1,732 (11.3)	2,593 (13.1)
Pharmacist	10,952 (9.5)	9,210 (11.4)	945 (6.2)	797 (4.0)
Other or missing	12,690 (11.0)	10,211 (12.7)	1,321 (8.6)	1,158 (5.9)
Breast cancer recorded as the indication	67,306 (58.1)	44,922 (55.7)	10,155 (66.2)	12,229 (61.9)
Concomitant endocrine therapy recorded	35,610 (30.8)	25,576 (31.7)	3,283 (21.4)	6,751 (34.2)
Time to onset available	23,670 (20.4)	14,424 (17.9)	3,227 (21.1)	6,019 (30.5)
Reported time to onset, median (IQR), days	65.0 (16.0–261.0)	84.0 (20.0–316.0)	32.0 (10.0–116.0)	57.0 (15.0–235.0)
AESI reported				
Hematologic toxicity	25,978 (22.4)	19,685 (24.4)	2,112 (13.8)	4,181 (21.2)
Gastrointestinal toxicity	24,116 (20.8)	14,412 (17.9)	5,780 (37.7)	3,924 (19.9)
Hepatobiliary toxicity	5,855 (5.1)	2,054 (2.5)	1,123 (7.3)	2,678 (13.6)
QT or cardiac toxicity	1,325 (1.1)	305 (0.4)	71 (0.5)	949 (4.8)
Venous thromboembolism or thrombotic events	2,421 (2.1)	1,432 (1.8)	515 (3.4)	474 (2.4)
Interstitial lung disease or pneumonitis	1,229 (1.1)	486 (0.6)	454 (3.0)	289 (1.5)
Infection or febrile events	6,978 (6.0)	4,817 (6.0)	636 (4.1)	1,525 (7.7)
Renal or creatinine abnormality	3,902 (3.4)	1,897 (2.4)	868 (5.7)	1,137 (5.8)

Values are presented as n (%), unless otherwise stated. The unit of analysis is the primary suspect individual case safety report. Percentages use the number of reports in each column as the denominator. Reported time to onset was summarized among reports with valid nonnegative intervals between treatment start and event date. A report could contribute to more than one AESI domain; AESI percentages are domain-specific. The 67,306 breast cancer indication reports shown here are restricted to the three-drug comparative dataset. Region and endocrine/concomitant medication context are summarized in Supplementary Tables S1 and S8. AESI, adverse events of special interest; FAERS, Food and Drug Administration Adverse Event Reporting System; IQR, interquartile range.

### FAERS AESI signal patterns

**FIGURE 2:**
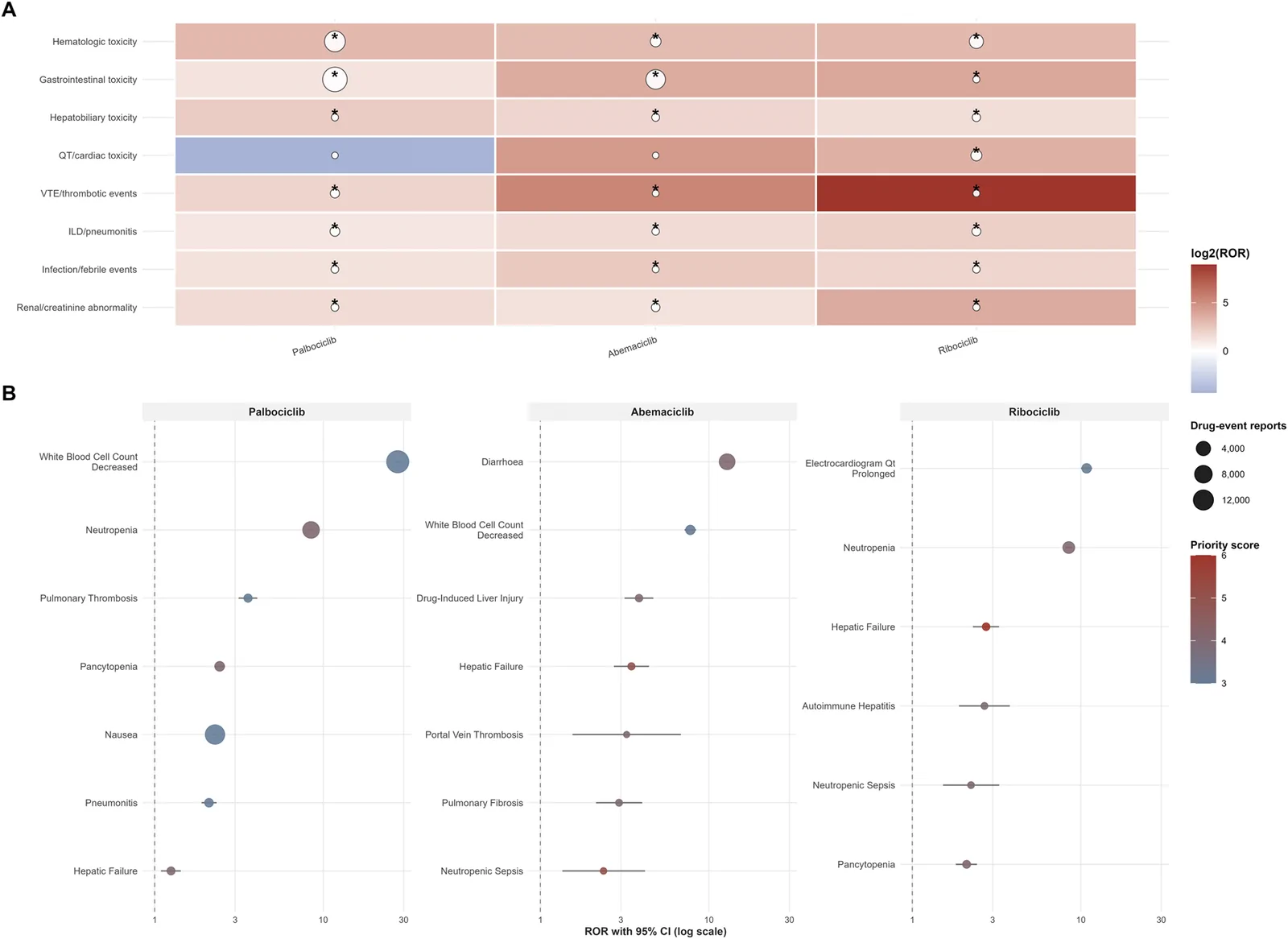
FAERS AESI signal patterns and representative preferred term signals. **(A)** Shows drug-by-AESI reporting signal patterns using log2 ROR and drug-event report counts; circle size indicates drug-event report counts, and asterisks denote concordant evidence across at least two prespecified signal-detection measures. **(B)** Shows representative PT signatures with ROR and 95% confidence intervals after clinical review and after excluding disease progression, metastasis, drug ineffectiveness, product issue, and indication-like terms. Displayed PT labels retain MedDRA preferred-term wording. PT cleaning sensitivity results and death/fatal outcome-handling analysis results are provided in Supplementary Table S15. The priority score reflects clinical review priority within the FAERS signal panel. AESI, adverse events of special interest; FAERS, Food and Drug Administration Adverse Event Reporting System; MedDRA, Medical Dictionary for Regulatory Activities; PT, preferred term; ROR, reporting odds ratio.

Figure 2 shows FAERS AESI reporting patterns and representative preferred term signal signatures. Palbociclib showed the most prominent hematologic reporting pattern, with neutropenia represented by 6,823 drug-event reports and an ROR of 8.47. Abemaciclib showed stronger gastrointestinal, pulmonary, thrombotic, and renal or creatinine-related reporting patterns, including diarrhea as a high-volume gastrointestinal signal. Ribociclib showed the clearest QT and cardiac and hepatobiliary signal profile, including QT prolongation and liver-related preferred terms. Representative preferred terms were selected after removing disease progression, metastasis, drug ineffectiveness, product issue, and indication-like concepts from the main visual panel. Representative preferred terms, excluded preferred terms, terms requiring clinical review, preferred term cleaning sensitivity results, and the death/fatal outcome-handling analysis are listed in Supplementary Tables S13–S15.

### Intraclass-adjusted reporting odds and reported TTO

**FIGURE 3:**
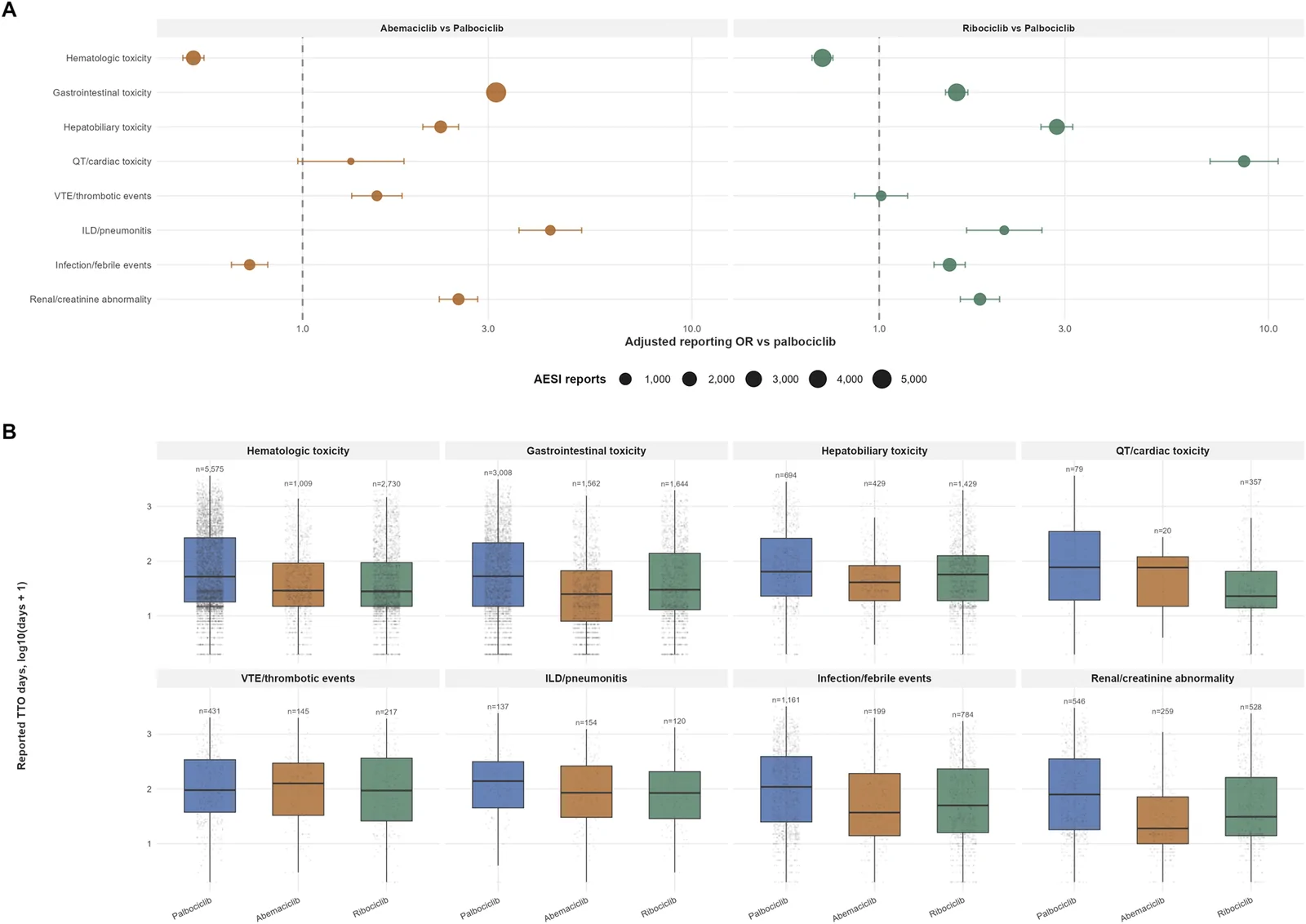
Intraclass-adjusted reporting odds and reported time to onset. **(A)** Shows adjusted reporting odds ratio (aROR) estimates within CDK4/6 inhibitor reports using palbociclib as the reference and adjustment for age, sex, report year, reporter country or region, and reporter type when models converged. **(B)** Shows reported TTO distributions on a log_10_(days +1) scale among reports with valid nonnegative TTO. Reported TTO is a report-based timing summary for safety-review hypothesis generation; TTO availability and interpretation boundaries are summarized in Supplementary Table S3. AESI, adverse events of special interest; aROR, adjusted reporting odds ratio; TTO, time to onset.

Figure 3 shows intraclass-adjusted reporting odds and reported TTO distributions. In intraclass-adjusted models, abemaciclib had higher reporting odds than palbociclib for gastrointestinal toxicity, with an adjusted reporting odds ratio (aROR) of 3.14 and a 95% confidence interval of 2.98–3.31; hepatobiliary toxicity, with an aROR of 2.26 and a 95% confidence interval of 2.04–2.52; venous thromboembolism and thrombotic events, with an aROR of 1.55 and a 95% confidence interval of 1.34–1.80; interstitial lung disease and pneumonitis, with an aROR of 4.33 and a 95% confidence interval of 3.60–5.21; and renal or creatinine abnormality, with an aROR of 2.52 and a 95% confidence interval of 2.25–2.82. Ribociclib had higher reporting odds than palbociclib for QT and cardiac toxicity, with an aROR of 8.66 and a 95% confidence interval of 7.08–10.59; hepatobiliary toxicity, with an aROR of 2.86 and a 95% confidence interval of 2.60–3.14; infection or febrile events, with an aROR of 1.52 and a 95% confidence interval of 1.38–1.66; and renal or creatinine abnormality, with an aROR of 1.82 and a 95% confidence interval of 1.62–2.04. Abemaciclib versus ribociclib direct contrasts are reported in Supplementary Table S2. Reported TTO distributions were based on reports with valid nonnegative intervals and are summarized with the availability summary in Supplementary Table S3.

### ML performance in temporal validation

**FIGURE 4:**
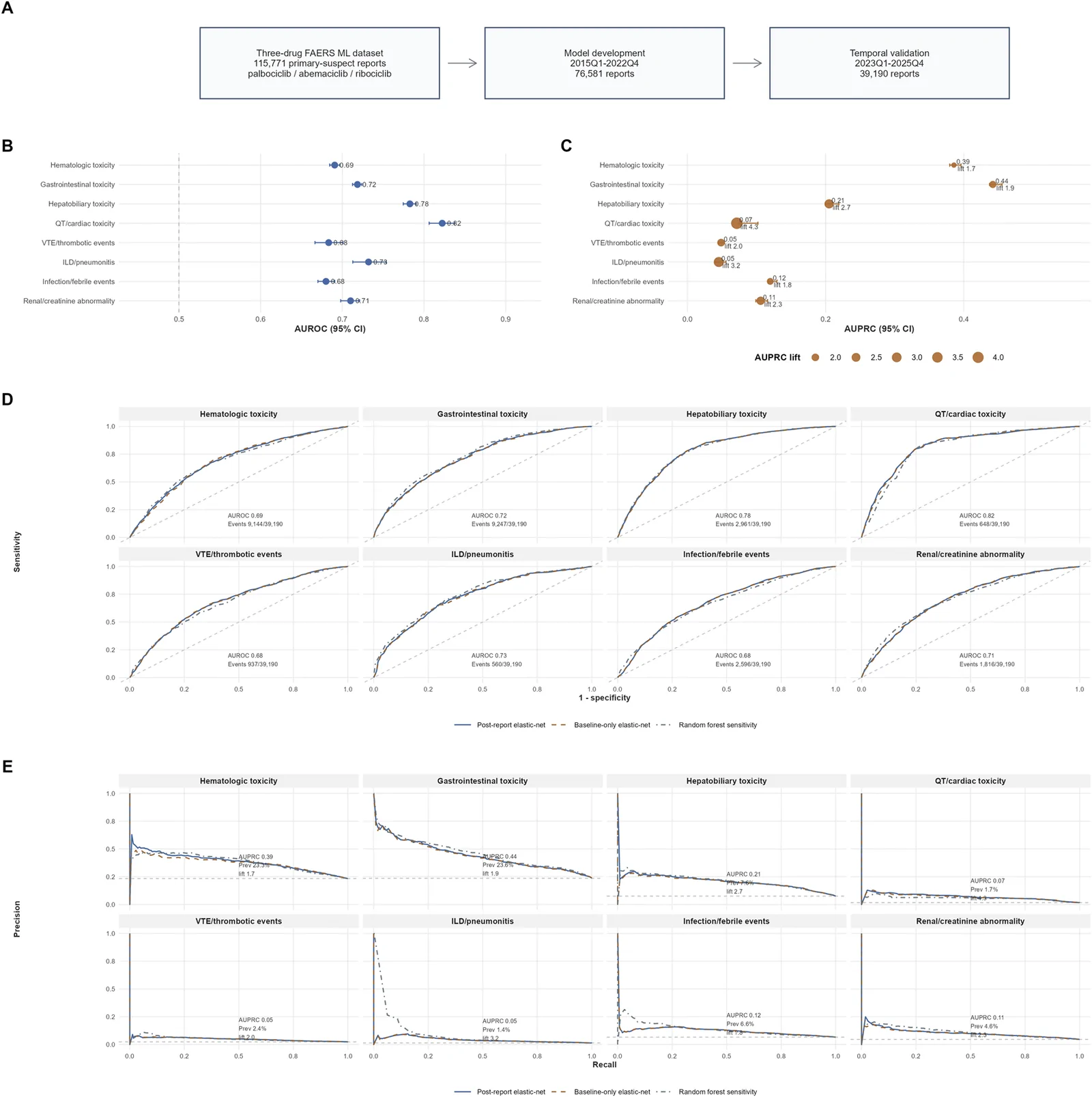
Machine learning prioritization performance in temporal validation. **(A)** Shows the three-drug ML dataset and temporal split. **(B,C)** Show AUROC, AUPRC, and AUPRC lift across AESI domains. **(D,E)** Show receiver operating characteristic and precision–recall curves in the temporal validation set. Curves describe report-level prioritization performance within FAERS, and incremental value beyond drug identity is summarized in Supplementary Table S6. AESI, adverse events of special interest; AUPRC, area under the precision–recall curve; AUROC, area under the receiver operating characteristic curve; FAERS, Food and Drug Administration Adverse Event Reporting System; ML, machine learning.

Figure 4 shows report-level prioritization model development, temporal validation performance, ROC curves, and precision–recall curves. In temporal validation, the primary post-report elastic-net models achieved AUROC values from 0.680 for infection and febrile events to 0.822 for QT and cardiac toxicity. AUPRC lift ranged from 1.654 for hematologic toxicity to 4.330 for QT and cardiac toxicity. For common AESIs, absolute AUPRC was higher, including 0.442 for gastrointestinal toxicity and 0.386 for hematologic toxicity. Rare AESIs had lower absolute AUPRC but higher enrichment over validation prevalence. Incremental values beyond drug identity and practical interpretation of ML performance by AESI are summarized in Supplementary Table S6.

### ML interpretability and prioritization

**FIGURE 5:**
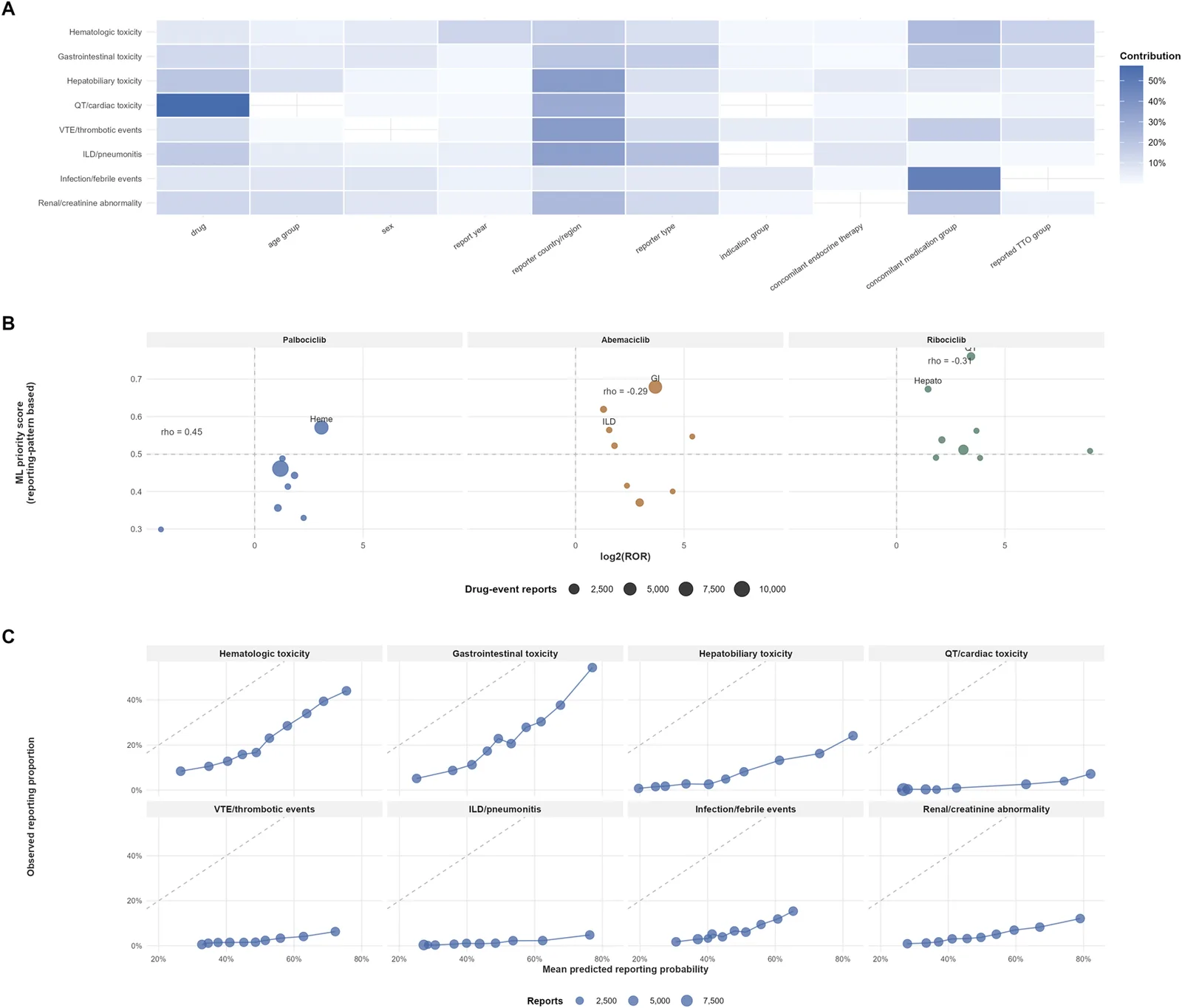
Model interpretability, prioritization, and calibration. **(A)** Shows grouped feature contribution in the post-report elastic-net model. **(B)** Compares ML priority scores with log_2_ ROR by drug. **(C)** Shows report-level calibration of FAERS prioritization strata across AESI domains. Discordant high-priority signals were retained as manual-review priorities. Grouped feature contributions and practical use of ML priority scores are summarized in Supplementary Table S7. AESI, adverse events of special interest; FAERS, Food and Drug Administration Adverse Event Reporting System; ML, machine learning; ROR, reporting odds ratio.

Figure 5 shows grouped feature contribution, concordance between ML priority and conventional log_2_ ROR, and calibration across FAERS prioritization strata. Drug identity, reporter country or region, reporter type, concomitant medication group, and reported TTO group contributed materially to several AESI models. ML priority scores showed partial concordance with conventional ROR, indicating that the models incorporated reporting context beyond conventional disproportionality. Discordant high-priority signals were flagged as manual-review priorities that combine model rank, signal metrics, literature context, and clinical plausibility. Grouped feature contributions and practical use of ML priority scores are provided in Supplementary Table S7, and a supplementary map of main figures and boundary statements is provided in Supplementary Table S16.

### Institutional clinical contextualization

**FIGURE 6:**
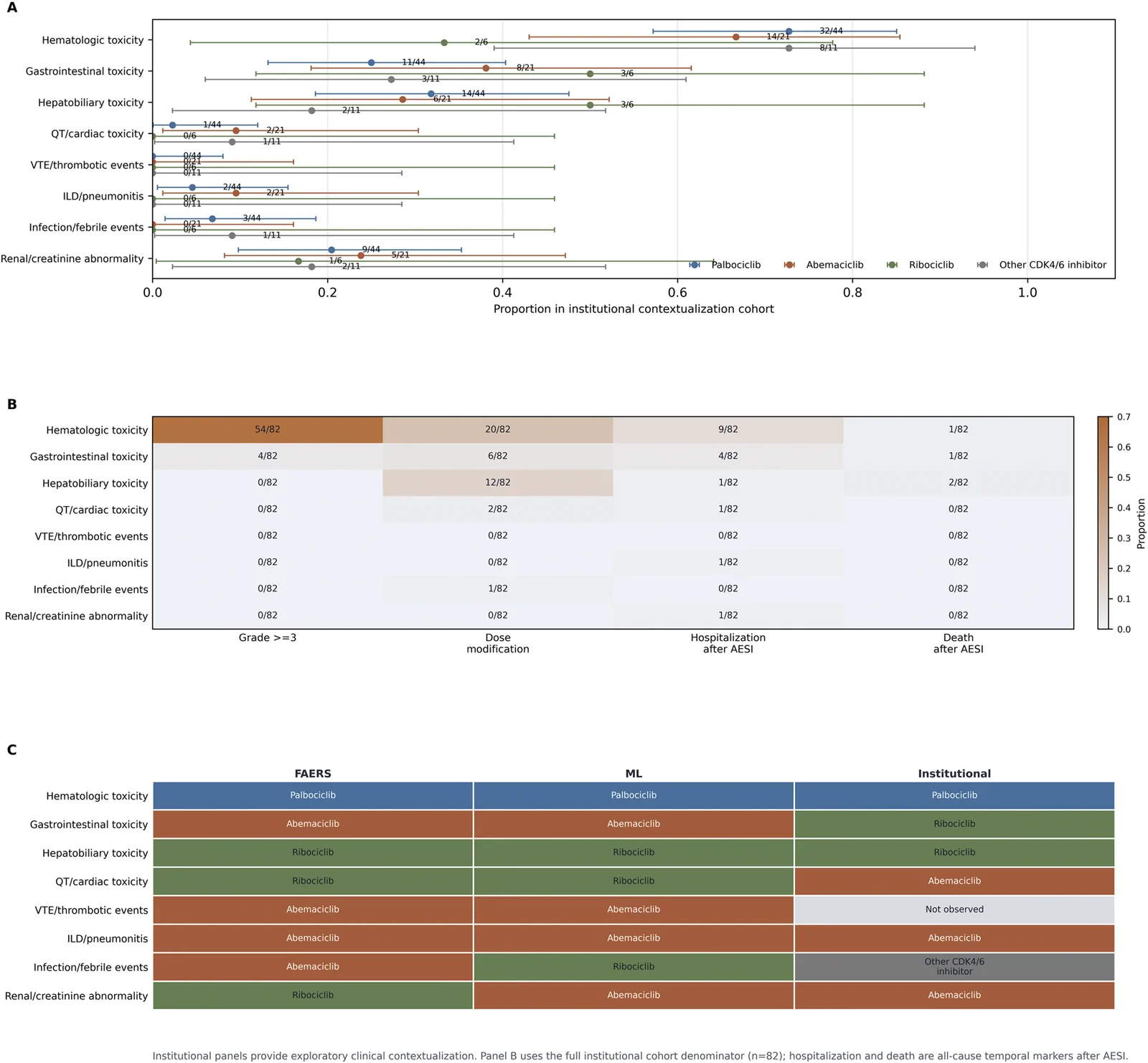
Institutional clinical contextualization of prioritized AESI. **(A)** Shows drug-specific clinically meaningful AESI proportions with exact 95% confidence intervals in the institutional contextualization cohort. **(B)** Shows grade 3 or higher toxicity, dose interruption, reduction, or discontinuation, all-cause hospitalization, and all-cause death occurring after documented AESI as temporal, non-attributional consequence markers; n/82 labels indicate the number of affected patients among all 82 cohort members for domain-specific clinical consequences. **(C)** Summarizes the top-drug context across FAERS, ML, and institutional layers for each AESI; institutional cells indicate the drug group with the highest observed local proportion or no observed event. Other CDK4/6 inhibitor recipients provide local clinical context. Supplementary figure mapping and the source-data summary for Figure 6 are summarized in Supplementary Table S16. AESI, adverse events of special interest; FAERS, Food and Drug Administration Adverse Event Reporting System; ML, machine learning.

Table 2 summarizes clinical characteristics, treatment characteristics, and treatment-emergent AESIs in the institutional cohort. The cohort included 82 patients, of whom 44 received palbociclib, 21 received abemaciclib, 6 received ribociclib, and 11 received other CDK4/6 inhibitors. These clinical data provided exploratory clinical contextualization. Figure 6 shows institutional clinical contextualization of FAERS-prioritized AESIs. The grade 3 or higher AESI row in Table 2 denotes patient-level occurrence across any AESI domain, and Figure 6B shows domain-specific clinical consequences using n/82 labels for grade 3 or higher toxicity, dose interruption/reduction/discontinuation, all-cause hospitalization, and all-cause death occurring after documented AESIs as temporal, non-attributional consequence markers. Clinically meaningful hematologic toxicity and grade 3 or higher hematologic toxicity were the most frequent institutional management correlates. Gastrointestinal and hepatobiliary AESIs were also observed, whereas several rare domains remained sparse in this single-center cohort. Endocrine partner context, concomitant medication strata, variable availability, and the source data summary for Figure 6 are provided in Supplementary Tables S8, S10, S12, S16.

**TABLE 2:** Clinical characteristics and treatment-emergent AESIs in the institutional cohort.

Characteristic	Overall (N = 82)	Palbociclib (N = 44)	Abemaciclib (N = 21)	Ribociclib (N = 6)	Other CDK4/6 inhibitor (N = 11)
Patient characteristics					
Age, median (IQR), years	56.0 (47.2–62.8)	57.0 (48.8–62.2)	58.0 (47.0–63.0)	48.5 (43.2–59.8)	54.0 (50.5–59.5)
Postmenopausal status	46 (56.1)	27 (61.4)	9 (42.9)	6 (100.0)	4 (36.4)
Stage IV disease	65 (79.3)	34 (77.3)	17 (81.0)	5 (83.3)	9 (81.8)
HR-positive and HER2-negative disease	80 (97.6)	42 (95.5)	21 (100.0)	6 (100.0)	11 (100.0)
Visceral metastasis	36 (43.9)	21 (47.7)	11 (52.4)	2 (33.3)	2 (18.2)
Bone metastasis	34 (41.5)	16 (36.4)	5 (23.8)	4 (66.7)	9 (81.8)
Liver metastasis	12 (14.6)	6 (13.6)	4 (19.0)	1 (16.7)	1 (9.1)
Lung metastasis	10 (12.2)	5 (11.4)	4 (19.0)	0 (0.0)	1 (9.1)
Brain metastasis	2 (2.4)	1 (2.3)	0 (0.0)	1 (16.7)	0 (0.0)
Diabetes	17 (20.7)	5 (11.4)	6 (28.6)	2 (33.3)	4 (36.4)
Cardiovascular disease	9 (11.0)	6 (13.6)	3 (14.3)	0 (0.0)	0 (0.0)
Baseline hepatic or renal dysfunction	1 (1.2)	1 (2.3)	0 (0.0)	0 (0.0)	0 (0.0)
Treatment characteristics					
Endocrine partner					
Fulvestrant	25 (30.5)	13 (29.5)	5 (23.8)	3 (50.0)	4 (36.4)
Aromatase inhibitor	57 (69.5)	31 (70.5)	16 (76.2)	3 (50.0)	7 (63.6)
Follow-up duration, median (IQR), months	28.9 (22.2–37.1)	27.6 (21.9–35.2)	37.0 (28.5–44.1)	27.7 (22.4–35.9)	23.4 (21.4–31.9)
Treatment-emergent AESI					
Any clinically meaningful AESI	76 (92.7)	41 (93.2)	18 (85.7)	6 (100.0)	11 (100.0)
Grade 3 or higher AESI	55 (67.1)	31 (70.5)	14 (66.7)	2 (33.3)	8 (72.7)
Dose interruption, reduction, or discontinuation after AESI	39 (47.6)	23 (52.3)	10 (47.6)	3 (50.0)	3 (27.3)
All-cause hospitalization after AESI	11 (13.4)	7 (15.9)	1 (4.8)	1 (16.7)	2 (18.2)
All-cause death after AESI	3 (3.7)	3 (6.8)	0 (0.0)	0 (0.0)	0 (0.0)
AESI domains					
Hematologic toxicity	56 (68.3)	32 (72.7)	14 (66.7)	2 (33.3)	8 (72.7)
Gastrointestinal toxicity	25 (30.5)	11 (25.0)	8 (38.1)	3 (50.0)	3 (27.3)
Hepatobiliary toxicity	25 (30.5)	14 (31.8)	6 (28.6)	3 (50.0)	2 (18.2)
QT or cardiac toxicity	4 (4.9)	1 (2.3)	2 (9.5)	0 (0.0)	1 (9.1)
Venous thromboembolism or thrombotic events	0 (0.0)	0 (0.0)	0 (0.0)	0 (0.0)	0 (0.0)
Interstitial lung disease or pneumonitis	4 (4.9)	2 (4.5)	2 (9.5)	0 (0.0)	0 (0.0)
Infection or febrile events	4 (4.9)	3 (6.8)	0 (0.0)	0 (0.0)	1 (9.1)
Renal or creatinine abnormality	17 (20.7)	9 (20.5)	5 (23.8)	1 (16.7)	2 (18.2)

Values are presented as n (%), unless otherwise stated. Percentages use the number of patients in each treatment column as the denominator. All patients in the institutional cohort were female, and all patients had an endocrine partner recorded. The institutional cohort retained two patients outside the HR-positive and HER2-negative descriptor because this component contextualized documented CDK4/6 inhibitor exposure for safety review. Treatment-emergent AESIs required new onset or worsening after CDK4/6 inhibitor initiation, plus event-specific clinical evidence, such as laboratory abnormality, explicit clinical documentation, or toxicity-related dose interruption, reduction, or discontinuation. Any clinically meaningful AESI is a patient-level variable, and each patient is counted once irrespective of the number of AESI domains. Grade 3 or higher AESIs denote patient-level occurrences of grade 3 or higher toxicity in any AESI domain. Grade 3 or higher events were recoded from laboratory or clinical documentation and aligned with Common Terminology Criteria for Adverse Events thresholds where sufficient information was available. All-cause hospitalization and all-cause death occurring after a documented AESI contributed temporal clinical consequence markers. Patients could have more than one AESI domain; AESI percentages are domain-specific. Other CDK4/6 inhibitors include local or region-specific CDK4/6 inhibitor exposure outside the FAERS three-drug comparative models; AESI, adverse events of special interest; HER2, human epidermal growth factor receptor 2; HR, hormone receptor; IQR, interquartile range.

## Discussion

This study prioritized drug-specific AESI reporting patterns for the three main breast cancer CDK4/6 inhibitors and connected those patterns to report-level ML triage and single-center clinical contextualization. The main contribution is the integration of broad FAERS class screening, multidomain AESI mapping, intraclass-adjusted reporting odds, reported TTO, temporal ML signal prioritization, and separate patient-level institutional management correlates in a single combined analysis. Palbociclib, abemaciclib, and ribociclib showed distinct and clinically interpretable reporting profiles that aligned partly across conventional disproportionality, ML prioritization, and institutional context. This combined structure is useful for pharmacovigilance review because it ranks signals for manual assessment while preserving the differences between spontaneous reporting, model-based triage, and bedside toxicity management.

Palbociclib findings mainly contributed to hematologic and dose-management review domains, extending PALOMA and comparative safety evidence into a post-marketing prioritization layer (Finn et al., 2016; Diéras et al., 2019; Verma et al., 2016). Exposure-response evidence for palbociclib and MONALEESA dose-reduction evidence for ribociclib contextualize dose-modification signals (Zheng et al., 2021; Buijs et al., 2025; Burris et al., 2021). The hematologic signal and early reported TTO can prioritize early-cycle neutropenia-related narratives, laboratory data, and dose-modification records for manual pharmacovigilance review. For example, within a pharmacovigilance review queue, a palbociclib report with early neutropenia-related TTO can be reviewed for narrative coherence, laboratory severity, and dose-modification documentation rather than treated as an incidence or monitoring-schedule estimate; denominator-based clinical protocols remain the source for monitoring schedules.

Abemaciclib findings mainly contributed to gastrointestinal, thrombotic, pulmonary, and renal- or creatinine-related review domains, consistent with post-marketing and translational evidence (Hua et al., 2024; Gao et al., 2023; Shu et al., 2023; Zhu et al., 2025). These domains provide focused safety-review hypotheses when interpreted alongside clinical trial, product label, and denominator-based clinical evidence.

Ribociclib findings mainly contributed to QT/cardiac, hepatobiliary, electrolyte, and interaction-review domains, aligning with QT-focused FAERS evidence, hepatic safety synthesis, MONALEESA survival data, and recent regulatory expansion (Hortobagyi et al., 2022; Gao et al., 2025; Yan et al., 2024; Tang et al., 2025; Im et al., 2019; Slamon et al., 2020). These domains provide focused pharmacovigilance review priorities when interpreted alongside trial, label, and denominator-based clinical evidence.

The ML component adds a second triage layer by combining drug identity with reporting context. Prior pharmacovigilance studies have used multivariable models, machine learning methods, and timing features to prioritize individual case safety reports and signal candidates for review (Al-Azzawi et al., 2023; Battini et al., 2024; Gosselt et al., 2022; Dauner et al., 2023). In this study, the strongest temporal validation performance was observed for the QT and cardiac toxicity and hepatobiliary toxicity domains, which have more drug-specific reporting structures and more concentrated terminology. Venous thromboembolism, infection or febrile events, and renal or creatinine abnormality showed more modest discrimination, consistent with multifactorial etiology, oncology comorbidity, supportive care variation, concomitant treatment, and report-level missingness. Available proxy features captured reporting context and concomitant medication burden, while the variable availability summary documents structured clinical variables for future denominator-based modeling. TRIPOD + AI and PROBAST + AI provide reporting and appraisal guidance for this model layer (Collins et al., 2024; Moons et al., 2025), and broader prediction model evidence reinforces the value of benchmarking ML models against simpler clinical or reporting-context predictors (Christodoulou et al., 2019). The practical use of the ML priority score is to rank reports and AESI-drug pairs for manual pharmacovigilance review, with drug identity, ROR, reporter type, region, concomitant medication group, endocrine-therapy recording, and TTO considered together.

FAERS provides broad post-marketing coverage through spontaneous reports, and reporting behavior shapes its interpretation. A systematic review estimated a median underreporting rate of 94% across spontaneous-reporting studies, and updated evidence shows that healthcare professional, event, and system factors influence underreporting (Hazell and Shakir, 2006; García-Abeijon et al., 2023). Spontaneous report counts and disproportionality metrics therefore describe reporting patterns within the submitted case set, a distinction emphasized in methodological work on spontaneous report interpretation and disproportionality analysis (Kant et al., 2022; van Puijenbroek et al., 2002; Cutroneo et al., 2023). This reporting structure is especially relevant for common lower-grade events such as abemaciclib-associated mild diarrhea, where clinical frequency and spontaneous reporting volume measure different constructs. Denominator-based clinical datasets with standardized severity capture frequency and absolute-risk questions for mild diarrhea and other low-grade events. Comparator choice can also create quasi-quantitative bias in pharmacovigilance, supporting the inclusion of intraclass comparisons and abemaciclib-versus-ribociclib contrasts (Grave et al., 2024). Death/fatal information contributed a report outcome and seriousness layer, adding severity context, while the PT signal panel focused on clinically specific adverse-event concepts. Reported TTO contributed a timing summary of submitted cases and guided safety-review hypotheses.

The institutional cohort adds exploratory clinical contextualization from a Chinese tertiary cancer center. This setting links prioritized signals to concrete management consequences, including grade 3 or higher toxicity, dose interruption, dose reduction, dose discontinuation, all-cause hospitalization, and all-cause death occurring after documented AESIs as temporal clinical consequence markers. Safety ascertainment reflected the center’s routine safety-monitoring workflow through available clinical documentation, laboratory testing, imaging, dose-modification records, hospitalization records, and survival status. China/Asia trial and real-world data provide a local practice context that may differ from Western trial populations (Zhang et al., 2023; Zhang et al., 2024; Zhao et al., 2026; Chen et al., 2024). The local cohort therefore functions as a patient-level context layer for FAERS and ML triage. Available endocrine-partner and concomitant-medication strata contextualize co-treatment patterns, and the variable-availability summary records prior-chemotherapy and treatment-line fields for future denominator-based modeling. These themes organize pharmacovigilance review pathways, and denominator-based clinical cohorts, clinical trials, labels, and guidelines remain the evidence base for absolute risk estimates and practice recommendations.

These data define the scope of inference: FAERS supports signal prioritization, and denominator-based cohorts remain necessary for incidence, causal contrast, and patient-level risk modeling. The ML models reflect the available report-level features; future denominator-based models can incorporate structured variables for cancer stage, comorbidity, treatment line, laboratory trajectories, prior therapy, and supportive care. The institutional cohort adds patient-level clinical context from consecutive screening at one Chinese tertiary center, and multicenter data can test how routine monitoring protocols, dose management, ethnicity, frailty, and treatment sequence modify the observed patterns. The current analysis combines signal triage in FAERS, temporal ML prioritization, and real-world clinical contextualization.

## Conclusion

Palbociclib, abemaciclib, and ribociclib showed distinct report-level FAERS profiles. ML signal prioritization and single-center clinical contextualization organized these patterns into drug-tailored pharmacovigilance review priorities and focused safety-review hypotheses for CDK4/6 inhibitor AESIs.

## Data Availability

The original contributions presented in the study are included in the article/Supplementary Material; further inquiries can be directed to the corresponding author.
