# Supplementary material for: Comparative safety signal prioritization of CDK4/6 inhibitors in breast cancer: a FAERS pharmacovigilance, machine learning, and clinical contextualization study
Source: Front Pharmacol. 2026 Aug 19;17:1889178. doi: 10.3389/fphar.2026.1889178 (PMC13534059; doi:10.3389/fphar.2026.1889178)
Supplement: Supplementary file 1 [file DataSheet1.pdf]

Supplementary material

This supplementary file provides detailed methods and tabular evidence supporting the manuscript. FAERS and ML outputs describe reporting patterns for pharmacovigilance signal prioritization. The institutional cohort provides exploratory clinical contextualization from routine care.

Supplementary methods

Agent eligibility and analysis-set hierarchy. We screened candidate CDK4/6 agents broadly to preserve class completeness, then assigned analysis roles according to FAERS capture, breast cancer endocrine-therapy relevance, and comparability with palbociclib, abemaciclib, and ribociclib. The broad retrieval set supports class screening, whereas the three-agent comparative set supports intraclass reporting odds and ML prioritization.

FAERS data extraction and primary suspect report construction. We downloaded quarterly public Food and Drug Administration Adverse Event Reporting System files for 2015Q1 to 2025Q4 from the FDA website and constructed primary suspect individual case safety reports. The report was the unit of analysis. We used breast-cancer exact indication subsets for disease-context sensitivity and retained all-indication primary suspect reports where broader signal capture was required. MedDRA preferred term labels and other source-controlled PT strings are reproduced as controlled vocabulary labels and may retain their source spelling.

AESI mapping and preferred term cleaning. We mapped Medical Dictionary for Regulatory Activities preferred terms into prespecified clinical AESI domains. We excluded disease progression, metastasis, therapeutic ineffectiveness, product issue, and indication-like terms from the main visual preferred-term panel when they did not represent drug safety events. We handled generic death or fatal-outcome MedDRA preferred terms as report-level patient outcome/seriousness markers in the primary analysis and reported death counts descriptively. For transparency, the death/fatal outcome-handling analysis retains 20 generic death/fatal PT rows as descriptive outcome metadata, and the source-data file retains the full exclusion list for 22 non-specific or clinically ambiguous death-like PTs.

Disproportionality algorithms. We calculated reporting odds ratio, proportional reporting ratio, information component, and empirical Bayes geometric mean as spontaneous-reporting disproportionality measures. These outputs describe reporting signals for pharmacovigilance triage.

Intraclass adjusted reporting odds. For each AESI domain, we fitted adjusted reporting-odds models within CDK4/6 inhibitor primary suspect reports using palbociclib as the reference. Models compared abemaciclib and ribociclib with palbociclib and adjusted for age, sex, report year, reporter country or region, and reporter type when convergence permitted.

Reported TTO derivation. We calculated reported time to onset from therapy start date and event date when both dates were available and plausible. We excluded reports with missing, negative, or implausible intervals from TTO summaries. TTO distributions summarize reported timing patterns among submitted reports.

ML dataset and temporal split. We restricted the machine learning dataset to primary suspect reports for palbociclib, abemaciclib, and ribociclib. We used reports from 2015Q1 to 2022Q4 for model development and reports from 2023Q1 to 2025Q4 for temporal validation within FAERS.

ML model specification and leakage prevention. The primary classifier was a post-report elastic-net logistic regression. We used a baseline-only model and a random-forest model as sensitivity analyses. We used preferred terms only to define AESI labels and kept them out of the predictor set. We reserved serious outcome, death, hospitalization, and institutional outcomes outside the predictor set to prevent outcome leakage into signal prioritization. ML priority scores triage FAERS reports or signal domains for pharmacovigilance review; clinical and methodological confirmation define actionability.

Institutional cohort derivation. The institutional component was a single-center retrospective cohort from a tertiary cancer center in China. We screened consecutive patients with breast cancer who initiated a CDK4/6 inhibitor from 6 Jan 2023 to 29 Dec 2024 and censored safety follow-up on 21 Mar 2026. The Ethics Committee of Harbin Medical University Cancer Hospital, Harbin Medical University, reviewed and approved the institutional clinical component, approval number KY2026-36, and waived written informed consent because the study used deidentified retrospective clinical records. Toxicity ascertainment, dose management, supportive-care access, and monitoring practices may differ from other healthcare settings; the institutional cohort provides exploratory contextualization of signal patterns observed in FAERS.

Institutional AESI recoding and clinical consequence definitions. Clinically meaningful treatment-emergent AESI required new onset or worsening after CDK4/6 inhibitor initiation plus event-specific clinical evidence, such as laboratory abnormality, explicit clinical documentation, or toxicity-related dose interruption, reduction, or discontinuation. We analyzed all-cause hospitalization and all-cause death occurring after a documented AESI separately as temporal clinical-consequence markers.

Supplementary tables

Reviewer-requested analyses are incorporated into Supplementary Tables S1-S16 using single-header long-table structures; each Word table below has one complete column set.

NA denotes a cell that is not applicable to that evidence layer or not available in the source field; source-defined missing data are labelled Missing where applicable.

Table S1. Agent eligibility, reporter geography, and region-stratified AESI reporting patterns.

Rows were rebuilt from the agent-eligibility CSV and stage-1 reporter-region source sheets.

Evidence layer	Agent or drug	Search terms or stratum	AESI or role	Reports or capture	Percent or relevance	Analysis role	Interpretation boundary	Notes
Agent eligibility	Palbociclib	palbociclib; IBRANCE; common spelling/case variants	CDK4/6 antitumour endocrine-therapy agent	sufficient; all-indication primary-suspect reports=80682; breast-cancer exact subset=44922	high; HR+/HER2- breast-cancer endocrine-therapy context	main comparative analysis	prespecified clinical comparability and sufficient FAERS capture	Included in disproportionality, intraclass adjusted reporting OR, TTO and ML signal-prioritization analyses.
Agent eligibility	Abemaciclib	abemaciclib; VERZENIO; common spelling/case variants	CDK4/6 antitumour endocrine-therapy agent	sufficient; all-indication primary-suspect reports=15330; breast-cancer exact subset=10155	high; HR+/HER2- breast-cancer endocrine-therapy context	main comparative analysis	prespecified clinical comparability and sufficient FAERS capture	Included in disproportionality, intraclass adjusted reporting OR, TTO and ML signal-prioritization analyses.
Agent eligibility	Ribociclib	ribociclib; KISQALI; common spelling/case variants	CDK4/6 antitumour endocrine-therapy agent	sufficient; all-indication primary-suspect reports=19759; breast-cancer exact subset=12229	high; HR+/HER2- breast-cancer endocrine-therapy context	main comparative analysis	prespecified clinical comparability and sufficient FAERS capture	Included in disproportionality, intraclass adjusted reporting OR, TTO and ML signal-prioritization analyses.
Agent eligibility	Dalpiciclib	dalpiciclib; SHR6390; common spelling/case variants	CDK4/6 antitumour agent with regional clinical use	insufficient in FAERS; all-indication primary-suspect reports=0; breast-cancer exact subset=0	region-dependent; not adequately captured in FAERS	excluded from three-drug comparative models; descriptive only if locally observed	prespecified data-sufficiency criterion not met; FAERS regional coverage limitation	Absence of FAERS capture should not be interpreted as absence of adverse events.
Agent eligibility	Trilaciclib	trilaciclib; COSELA; common spelling/case variants	chemotherapy myeloprotection agent outside the endocrine-therapy antitumour	small and clinically heterogeneous for this research question; all-indication primary-	clinically heterogeneous relative to breast-cancer endocrine-therapy	excluded from three-drug comparative models; descriptive only	prespecified clinical-comparability criterion not met	Not pooled with palbociclib/abemaciclib/ribociclib to avoid clinical heterogeneity.

Evidence layer	Agent or drug	Search terms or stratum	AESI or role	Reports or capture	Percent or relevance	Analysis role	Interpretation boundary	Notes
			CDK4/6 treatment context	suspect reports=200; breast-cancer exact subset=8	CDK4/6 antitumour agents			
Reporter region distribution	Abemaciclib	China/region	NA	756	4.9%	Reporter geography context	Describes FAERS reporting composition within submitted reports.	Within-drug percentage.
Reporter region distribution	Palbociclib	China/region	NA	709	0.9%	Reporter geography context	Describes FAERS reporting composition within submitted reports.	Within-drug percentage.
Reporter region distribution	Ribociclib	China/region	NA	231	1.2%	Reporter geography context	Describes FAERS reporting composition within submitted reports.	Within-drug percentage.
Reporter region distribution	Abemaciclib	Europe	NA	1,272	8.3%	Reporter geography context	Describes FAERS reporting composition within submitted reports.	Within-drug percentage.
Reporter region distribution	Palbociclib	Europe	NA	2,779	3.4%	Reporter geography context	Describes FAERS reporting composition within submitted reports.	Within-drug percentage.
Reporter region distribution	Ribociclib	Europe	NA	3,533	17.9%	Reporter geography context	Describes FAERS reporting composition within submitted reports.	Within-drug percentage.
Reporter region distribution	Abemaciclib	Japan	NA	783	5.1%	Reporter geography context	Describes FAERS reporting composition within submitted reports.	Within-drug percentage.
Reporter region distribution	Palbociclib	Japan	NA	1,190	1.5%	Reporter geography context	Describes FAERS reporting composition within submitted reports.	Within-drug percentage.
Reporter region distribution	Ribociclib	Japan	NA	6	0.0%	Reporter geography context	Describes FAERS reporting composition within submitted reports.	Within-drug percentage.
Reporter region distribution	Palbociclib	Missing	NA	1	0.0%	Reporter geography context	Describes FAERS reporting composition within submitted reports.	Within-drug percentage.
Reporter region distribution	Ribociclib	Missing	NA	1	0.0%	Reporter geography context	Describes FAERS reporting composition within submitted reports.	Within-drug percentage.
Reporter region distribution	Abemaciclib	Other	NA	1,059	6.9%	Reporter geography context	Describes FAERS reporting composition within submitted reports.	Within-drug percentage.
Reporter region	Palbociclib	Other	NA	7,653	9.5%	Reporter geography	Describes FAERS	Within-drug percentage.

Evidence layer	Agent or drug	Search terms or stratum	AESI or role	Reports or capture	Percent or relevance	Analysis role	Interpretation boundary	Notes
distribution						context	reporting composition within submitted reports.	
Reporter region distribution	Ribociclib	Other	NA	7,449	37.7%	Reporter geography context	Describes FAERS reporting composition within submitted reports.	Within-drug percentage.
Reporter region distribution	Abemaciclib	Other Asia	NA	157	1.0%	Reporter geography context	Describes FAERS reporting composition within submitted reports.	Within-drug percentage.
Reporter region distribution	Palbociclib	Other Asia	NA	2,767	3.4%	Reporter geography context	Describes FAERS reporting composition within submitted reports.	Within-drug percentage.
Reporter region distribution	Ribociclib	Other Asia	NA	1,181	6.0%	Reporter geography context	Describes FAERS reporting composition within submitted reports.	Within-drug percentage.
Reporter region distribution	Abemaciclib	United States	NA	11,303	73.7%	Reporter geography context	Describes FAERS reporting composition within submitted reports.	Within-drug percentage.
Reporter region distribution	Palbociclib	United States	NA	65,583	81.3%	Reporter geography context	Describes FAERS reporting composition within submitted reports.	Within-drug percentage.
Reporter region distribution	Ribociclib	United States	NA	7,358	37.2%	Reporter geography context	Describes FAERS reporting composition within submitted reports.	Within-drug percentage.
Region-stratified AESI reporting	Abemaciclib	China/region	Hematologic toxicity	472/756	62.4%	Region-stratified reporting context	Describes FAERS reporting composition for descriptive context.	Report-level descriptive stratification.
Region-stratified AESI reporting	Palbociclib	China/region	Hematologic toxicity	196/709	27.6%	Region-stratified reporting context	Describes FAERS reporting composition for descriptive context.	Report-level descriptive stratification.
Region-stratified AESI reporting	Ribociclib	China/region	Hematologic toxicity	134/231	58.0%	Region-stratified reporting context	Describes FAERS reporting composition for descriptive context.	Report-level descriptive stratification.
Region-stratified AESI reporting	Abemaciclib	Europe	Hematologic toxicity	243/1,272	19.1%	Region-stratified reporting context	Describes FAERS reporting composition for descriptive context.	Report-level descriptive stratification.
Region-stratified AESI reporting	Palbociclib	Europe	Hematologic toxicity	834/2,779	30.0%	Region-stratified reporting context	Describes FAERS reporting composition for descriptive context.	Report-level descriptive stratification.
Region-stratified AESI reporting	Ribociclib	Europe	Hematologic toxicity	1,013/3,533	28.7%	Region-stratified reporting context	Describes FAERS reporting composition for descriptive context.	Report-level descriptive stratification.
Region-stratified AESI reporting	Abemaciclib	Japan	Hematologic toxicity	151/783	19.3%	Region-stratified reporting context	Describes FAERS reporting composition	Report-level descriptive stratification.

Evidence layer	Agent or drug	Search terms or stratum	AESI or role	Reports or capture	Percent or relevance	Analysis role	Interpretation boundary	Notes
							for descriptive context.	
Region-stratified AESI reporting	Palbociclib	Japan	Hematologic toxicity	527/1,190	44.3%	Region-stratified reporting context	Describes FAERS reporting composition for descriptive context.	Report-level descriptive stratification.
Region-stratified AESI reporting	Ribociclib	Japan	Hematologic toxicity	1/6	16.7%	Region-stratified reporting context	Describes FAERS reporting composition for descriptive context.	Report-level descriptive stratification.
Region-stratified AESI reporting	Palbociclib	Missing	Hematologic toxicity	0/1	0.0%	Region-stratified reporting context	Describes FAERS reporting composition for descriptive context.	Report-level descriptive stratification.
Region-stratified AESI reporting	Ribociclib	Missing	Hematologic toxicity	0/1	0.0%	Region-stratified reporting context	Describes FAERS reporting composition for descriptive context.	Report-level descriptive stratification.
Region-stratified AESI reporting	Abemaciclib	Other	Hematologic toxicity	199/1,059	18.8%	Region-stratified reporting context	Describes FAERS reporting composition for descriptive context.	Report-level descriptive stratification.
Region-stratified AESI reporting	Palbociclib	Other	Hematologic toxicity	1,112/7,653	14.5%	Region-stratified reporting context	Describes FAERS reporting composition for descriptive context.	Report-level descriptive stratification.
Region-stratified AESI reporting	Ribociclib	Other	Hematologic toxicity	1,707/7,449	22.9%	Region-stratified reporting context	Describes FAERS reporting composition for descriptive context.	Report-level descriptive stratification.
Region-stratified AESI reporting	Abemaciclib	Other Asia	Hematologic toxicity	43/157	27.4%	Region-stratified reporting context	Describes FAERS reporting composition for descriptive context.	Report-level descriptive stratification.
Region-stratified AESI reporting	Palbociclib	Other Asia	Hematologic toxicity	800/2,767	28.9%	Region-stratified reporting context	Describes FAERS reporting composition for descriptive context.	Report-level descriptive stratification.
Region-stratified AESI reporting	Ribociclib	Other Asia	Hematologic toxicity	338/1,181	28.6%	Region-stratified reporting context	Describes FAERS reporting composition for descriptive context.	Report-level descriptive stratification.
Region-stratified AESI reporting	Abemaciclib	United States	Hematologic toxicity	1,004/11,303	8.9%	Region-stratified reporting context	Describes FAERS reporting composition for descriptive context.	Report-level descriptive stratification.
Region-stratified AESI reporting	Palbociclib	United States	Hematologic toxicity	16,216/65,583	24.7%	Region-stratified reporting context	Describes FAERS reporting composition for descriptive context.	Report-level descriptive stratification.
Region-stratified AESI reporting	Ribociclib	United States	Hematologic toxicity	988/7,358	13.4%	Region-stratified reporting context	Describes FAERS reporting composition for descriptive context.	Report-level descriptive stratification.
Region-stratified AESI reporting	Abemaciclib	China/region	Gastrointestinal toxicity	128/756	16.9%	Region-stratified reporting context	Describes FAERS reporting composition for descriptive context.	Report-level descriptive stratification.
Region-stratified AESI reporting	Palbociclib	China/region	Gastrointestinal toxicity	11/709	1.6%	Region-stratified reporting context	Describes FAERS reporting composition for descriptive context.	Report-level descriptive stratification.
Region-stratified AESI reporting	Ribociclib	China/region	Gastrointestinal toxicity	12/231	5.2%	Region-stratified reporting context	Describes FAERS reporting composition for descriptive context.	Report-level descriptive stratification.
Region-stratified AESI	Abemaciclib	Europe	Gastrointestinal toxicity	349/1,272	27.4%	Region-stratified	Describes FAERS	Report-level descriptive

Evidence layer	Agent or drug	Search terms or stratum	AESI or role	Reports or capture	Percent or relevance	Analysis role	Interpretation boundary	Notes
reporting						reporting context	reporting composition for descriptive context.	stratification.
Region-stratified AESI reporting	Palbociclib	Europe	Gastrointestinal toxicity	295/2,779	10.6%	Region-stratified reporting context	Describes FAERS reporting composition for descriptive context.	Report-level descriptive stratification.
Region-stratified AESI reporting	Ribociclib	Europe	Gastrointestinal toxicity	557/3,533	15.8%	Region-stratified reporting context	Describes FAERS reporting composition for descriptive context.	Report-level descriptive stratification.
Region-stratified AESI reporting	Abemaciclib	Japan	Gastrointestinal toxicity	174/783	22.2%	Region-stratified reporting context	Describes FAERS reporting composition for descriptive context.	Report-level descriptive stratification.
Region-stratified AESI reporting	Palbociclib	Japan	Gastrointestinal toxicity	99/1,190	8.3%	Region-stratified reporting context	Describes FAERS reporting composition for descriptive context.	Report-level descriptive stratification.
Region-stratified AESI reporting	Ribociclib	Japan	Gastrointestinal toxicity	1/6	16.7%	Region-stratified reporting context	Describes FAERS reporting composition for descriptive context.	Report-level descriptive stratification.
Region-stratified AESI reporting	Palbociclib	Missing	Gastrointestinal toxicity	1/1	100.0%	Region-stratified reporting context	Describes FAERS reporting composition for descriptive context.	Report-level descriptive stratification.
Region-stratified AESI reporting	Ribociclib	Missing	Gastrointestinal toxicity	0/1	0.0%	Region-stratified reporting context	Describes FAERS reporting composition for descriptive context.	Report-level descriptive stratification.
Region-stratified AESI reporting	Abemaciclib	Other	Gastrointestinal toxicity	269/1,059	25.4%	Region-stratified reporting context	Describes FAERS reporting composition for descriptive context.	Report-level descriptive stratification.
Region-stratified AESI reporting	Palbociclib	Other	Gastrointestinal toxicity	557/7,653	7.3%	Region-stratified reporting context	Describes FAERS reporting composition for descriptive context.	Report-level descriptive stratification.
Region-stratified AESI reporting	Ribociclib	Other	Gastrointestinal toxicity	1,762/7,449	23.7%	Region-stratified reporting context	Describes FAERS reporting composition for descriptive context.	Report-level descriptive stratification.
Region-stratified AESI reporting	Abemaciclib	Other Asia	Gastrointestinal toxicity	35/157	22.3%	Region-stratified reporting context	Describes FAERS reporting composition for descriptive context.	Report-level descriptive stratification.
Region-stratified AESI reporting	Palbociclib	Other Asia	Gastrointestinal toxicity	305/2,767	11.0%	Region-stratified reporting context	Describes FAERS reporting composition for descriptive context.	Report-level descriptive stratification.
Region-stratified AESI reporting	Ribociclib	Other Asia	Gastrointestinal toxicity	143/1,181	12.1%	Region-stratified reporting context	Describes FAERS reporting composition for descriptive context.	Report-level descriptive stratification.
Region-stratified AESI reporting	Abemaciclib	United States	Gastrointestinal toxicity	4,825/11,303	42.7%	Region-stratified reporting context	Describes FAERS reporting composition for descriptive context.	Report-level descriptive stratification.
Region-stratified AESI reporting	Palbociclib	United States	Gastrointestinal toxicity	13,144/65,583	20.0%	Region-stratified reporting context	Describes FAERS reporting composition for descriptive context.	Report-level descriptive stratification.
Region-stratified AESI reporting	Ribociclib	United States	Gastrointestinal toxicity	1,449/7,358	19.7%	Region-stratified reporting context	Describes FAERS reporting composition for descriptive context.	Report-level descriptive stratification.

Evidence layer	Agent or drug	Search terms or stratum	AESI or role	Reports or capture	Percent or relevance	Analysis role	Interpretation boundary	Notes
Region-stratified AESI reporting	Abemaciclib	China/region	Hepatobiliary toxicity	130/756	17.2%	Region-stratified reporting context	Describes FAERS reporting composition for descriptive context.	Report-level descriptive stratification.
Region-stratified AESI reporting	Palbociclib	China/region	Hepatobiliary toxicity	40/709	5.6%	Region-stratified reporting context	Describes FAERS reporting composition for descriptive context.	Report-level descriptive stratification.
Region-stratified AESI reporting	Ribociclib	China/region	Hepatobiliary toxicity	25/231	10.8%	Region-stratified reporting context	Describes FAERS reporting composition for descriptive context.	Report-level descriptive stratification.
Region-stratified AESI reporting	Abemaciclib	Europe	Hepatobiliary toxicity	258/1,272	20.3%	Region-stratified reporting context	Describes FAERS reporting composition for descriptive context.	Report-level descriptive stratification.
Region-stratified AESI reporting	Palbociclib	Europe	Hepatobiliary toxicity	305/2,779	11.0%	Region-stratified reporting context	Describes FAERS reporting composition for descriptive context.	Report-level descriptive stratification.
Region-stratified AESI reporting	Ribociclib	Europe	Hepatobiliary toxicity	691/3,533	19.6%	Region-stratified reporting context	Describes FAERS reporting composition for descriptive context.	Report-level descriptive stratification.
Region-stratified AESI reporting	Abemaciclib	Japan	Hepatobiliary toxicity	233/783	29.8%	Region-stratified reporting context	Describes FAERS reporting composition for descriptive context.	Report-level descriptive stratification.
Region-stratified AESI reporting	Palbociclib	Japan	Hepatobiliary toxicity	127/1,190	10.7%	Region-stratified reporting context	Describes FAERS reporting composition for descriptive context.	Report-level descriptive stratification.
Region-stratified AESI reporting	Ribociclib	Japan	Hepatobiliary toxicity	1/6	16.7%	Region-stratified reporting context	Describes FAERS reporting composition for descriptive context.	Report-level descriptive stratification.
Region-stratified AESI reporting	Palbociclib	Missing	Hepatobiliary toxicity	0/1	0.0%	Region-stratified reporting context	Describes FAERS reporting composition for descriptive context.	Report-level descriptive stratification.
Region-stratified AESI reporting	Ribociclib	Missing	Hepatobiliary toxicity	0/1	0.0%	Region-stratified reporting context	Describes FAERS reporting composition for descriptive context.	Report-level descriptive stratification.
Region-stratified AESI reporting	Abemaciclib	Other	Hepatobiliary toxicity	147/1,059	13.9%	Region-stratified reporting context	Describes FAERS reporting composition for descriptive context.	Report-level descriptive stratification.
Region-stratified AESI reporting	Palbociclib	Other	Hepatobiliary toxicity	333/7,653	4.4%	Region-stratified reporting context	Describes FAERS reporting composition for descriptive context.	Report-level descriptive stratification.
Region-stratified AESI reporting	Ribociclib	Other	Hepatobiliary toxicity	1,109/7,449	14.9%	Region-stratified reporting context	Describes FAERS reporting composition for descriptive context.	Report-level descriptive stratification.
Region-stratified AESI reporting	Abemaciclib	Other Asia	Hepatobiliary toxicity	17/157	10.8%	Region-stratified reporting context	Describes FAERS reporting composition for descriptive context.	Report-level descriptive stratification.
Region-stratified AESI reporting	Palbociclib	Other Asia	Hepatobiliary toxicity	210/2,767	7.6%	Region-stratified reporting context	Describes FAERS reporting composition for descriptive context.	Report-level descriptive stratification.
Region-stratified AESI reporting	Ribociclib	Other Asia	Hepatobiliary toxicity	179/1,181	15.2%	Region-stratified reporting context	Describes FAERS reporting composition	Report-level descriptive stratification.

Evidence layer	Agent or drug	Search terms or stratum	AESI or role	Reports or capture	Percent or relevance	Analysis role	Interpretation boundary	Notes
							for descriptive context.	
Region-stratified AESI reporting	Abemaciclib	United States	Hepatobiliary toxicity	338/11,303	3.0%	Region-stratified reporting context	Describes FAERS reporting composition for descriptive context.	Report-level descriptive stratification.
Region-stratified AESI reporting	Palbociclib	United States	Hepatobiliary toxicity	1,039/65,583	1.6%	Region-stratified reporting context	Describes FAERS reporting composition for descriptive context.	Report-level descriptive stratification.
Region-stratified AESI reporting	Ribociclib	United States	Hepatobiliary toxicity	673/7,358	9.1%	Region-stratified reporting context	Describes FAERS reporting composition for descriptive context.	Report-level descriptive stratification.
Region-stratified AESI reporting	Abemaciclib	China/region	QT/cardiac toxicity	4/756	0.5%	Region-stratified reporting context	Describes FAERS reporting composition for descriptive context.	Report-level descriptive stratification.
Region-stratified AESI reporting	Palbociclib	China/region	QT/cardiac toxicity	5/709	0.7%	Region-stratified reporting context	Describes FAERS reporting composition for descriptive context.	Report-level descriptive stratification.
Region-stratified AESI reporting	Ribociclib	China/region	QT/cardiac toxicity	8/231	3.5%	Region-stratified reporting context	Describes FAERS reporting composition for descriptive context.	Report-level descriptive stratification.
Region-stratified AESI reporting	Abemaciclib	Europe	QT/cardiac toxicity	9/1,272	0.7%	Region-stratified reporting context	Describes FAERS reporting composition for descriptive context.	Report-level descriptive stratification.
Region-stratified AESI reporting	Palbociclib	Europe	QT/cardiac toxicity	63/2,779	2.3%	Region-stratified reporting context	Describes FAERS reporting composition for descriptive context.	Report-level descriptive stratification.
Region-stratified AESI reporting	Ribociclib	Europe	QT/cardiac toxicity	257/3,533	7.3%	Region-stratified reporting context	Describes FAERS reporting composition for descriptive context.	Report-level descriptive stratification.
Region-stratified AESI reporting	Abemaciclib	Japan	QT/cardiac toxicity	10/783	1.3%	Region-stratified reporting context	Describes FAERS reporting composition for descriptive context.	Report-level descriptive stratification.
Region-stratified AESI reporting	Palbociclib	Japan	QT/cardiac toxicity	3/1,190	0.3%	Region-stratified reporting context	Describes FAERS reporting composition for descriptive context.	Report-level descriptive stratification.
Region-stratified AESI reporting	Ribociclib	Japan	QT/cardiac toxicity	0/6	0.0%	Region-stratified reporting context	Describes FAERS reporting composition for descriptive context.	Report-level descriptive stratification.
Region-stratified AESI reporting	Palbociclib	Missing	QT/cardiac toxicity	0/1	0.0%	Region-stratified reporting context	Describes FAERS reporting composition for descriptive context.	Report-level descriptive stratification.
Region-stratified AESI reporting	Ribociclib	Missing	QT/cardiac toxicity	0/1	0.0%	Region-stratified reporting context	Describes FAERS reporting composition for descriptive context.	Report-level descriptive stratification.
Region-stratified AESI reporting	Abemaciclib	Other	QT/cardiac toxicity	8/1,059	0.8%	Region-stratified reporting context	Describes FAERS reporting composition for descriptive context.	Report-level descriptive stratification.
Region-stratified AESI reporting	Palbociclib	Other	QT/cardiac toxicity	47/7,653	0.6%	Region-stratified reporting context	Describes FAERS reporting composition for descriptive context.	Report-level descriptive stratification.
Region-stratified AESI	Ribociclib	Other	QT/cardiac toxicity	366/7,449	4.9%	Region-stratified	Describes FAERS	Report-level descriptive

Evidence layer	Agent or drug	Search terms or stratum	AESI or role	Reports or capture	Percent or relevance	Analysis role	Interpretation boundary	Notes
reporting						reporting context	reporting composition for descriptive context.	stratification.
Region-stratified AESI reporting	Abemaciclib	Other Asia	QT/cardiac toxicity	0/157	0.0%	Region-stratified reporting context	Describes FAERS reporting composition for descriptive context.	Report-level descriptive stratification.
Region-stratified AESI reporting	Palbociclib	Other Asia	QT/cardiac toxicity	13/2,767	0.5%	Region-stratified reporting context	Describes FAERS reporting composition for descriptive context.	Report-level descriptive stratification.
Region-stratified AESI reporting	Ribociclib	Other Asia	QT/cardiac toxicity	55/1,181	4.7%	Region-stratified reporting context	Describes FAERS reporting composition for descriptive context.	Report-level descriptive stratification.
Region-stratified AESI reporting	Abemaciclib	United States	QT/cardiac toxicity	40/11,303	0.4%	Region-stratified reporting context	Describes FAERS reporting composition for descriptive context.	Report-level descriptive stratification.
Region-stratified AESI reporting	Palbociclib	United States	QT/cardiac toxicity	174/65,583	0.3%	Region-stratified reporting context	Describes FAERS reporting composition for descriptive context.	Report-level descriptive stratification.
Region-stratified AESI reporting	Ribociclib	United States	QT/cardiac toxicity	263/7,358	3.6%	Region-stratified reporting context	Describes FAERS reporting composition for descriptive context.	Report-level descriptive stratification.
Region-stratified AESI reporting	Abemaciclib	China/region	VTE/thrombotic events	48/756	6.3%	Region-stratified reporting context	Describes FAERS reporting composition for descriptive context.	Report-level descriptive stratification.
Region-stratified AESI reporting	Palbociclib	China/region	VTE/thrombotic events	5/709	0.7%	Region-stratified reporting context	Describes FAERS reporting composition for descriptive context.	Report-level descriptive stratification.
Region-stratified AESI reporting	Ribociclib	China/region	VTE/thrombotic events	0/231	0.0%	Region-stratified reporting context	Describes FAERS reporting composition for descriptive context.	Report-level descriptive stratification.
Region-stratified AESI reporting	Abemaciclib	Europe	VTE/thrombotic events	103/1,272	8.1%	Region-stratified reporting context	Describes FAERS reporting composition for descriptive context.	Report-level descriptive stratification.
Region-stratified AESI reporting	Palbociclib	Europe	VTE/thrombotic events	178/2,779	6.4%	Region-stratified reporting context	Describes FAERS reporting composition for descriptive context.	Report-level descriptive stratification.
Region-stratified AESI reporting	Ribociclib	Europe	VTE/thrombotic events	143/3,533	4.0%	Region-stratified reporting context	Describes FAERS reporting composition for descriptive context.	Report-level descriptive stratification.
Region-stratified AESI reporting	Abemaciclib	Japan	VTE/thrombotic events	47/783	6.0%	Region-stratified reporting context	Describes FAERS reporting composition for descriptive context.	Report-level descriptive stratification.
Region-stratified AESI reporting	Palbociclib	Japan	VTE/thrombotic events	25/1,190	2.1%	Region-stratified reporting context	Describes FAERS reporting composition for descriptive context.	Report-level descriptive stratification.
Region-stratified AESI reporting	Ribociclib	Japan	VTE/thrombotic events	0/6	0.0%	Region-stratified reporting context	Describes FAERS reporting composition for descriptive context.	Report-level descriptive stratification.
Region-stratified AESI reporting	Palbociclib	Missing	VTE/thrombotic events	0/1	0.0%	Region-stratified reporting context	Describes FAERS reporting composition for descriptive context.	Report-level descriptive stratification.

Evidence layer	Agent or drug	Search terms or stratum	AESI or role	Reports or capture	Percent or relevance	Analysis role	Interpretation boundary	Notes
Region-stratified AESI reporting	Ribociclib	Missing	VTE/thrombotic events	0/1	0.0%	Region-stratified reporting context	Describes FAERS reporting composition for descriptive context.	Report-level descriptive stratification.
Region-stratified AESI reporting	Abemaciclib	Other	VTE/thrombotic events	96/1,059	9.1%	Region-stratified reporting context	Describes FAERS reporting composition for descriptive context.	Report-level descriptive stratification.
Region-stratified AESI reporting	Palbociclib	Other	VTE/thrombotic events	241/7,653	3.1%	Region-stratified reporting context	Describes FAERS reporting composition for descriptive context.	Report-level descriptive stratification.
Region-stratified AESI reporting	Ribociclib	Other	VTE/thrombotic events	234/7,449	3.1%	Region-stratified reporting context	Describes FAERS reporting composition for descriptive context.	Report-level descriptive stratification.
Region-stratified AESI reporting	Abemaciclib	Other Asia	VTE/thrombotic events	15/157	9.6%	Region-stratified reporting context	Describes FAERS reporting composition for descriptive context.	Report-level descriptive stratification.
Region-stratified AESI reporting	Palbociclib	Other Asia	VTE/thrombotic events	44/2,767	1.6%	Region-stratified reporting context	Describes FAERS reporting composition for descriptive context.	Report-level descriptive stratification.
Region-stratified AESI reporting	Ribociclib	Other Asia	VTE/thrombotic events	23/1,181	1.9%	Region-stratified reporting context	Describes FAERS reporting composition for descriptive context.	Report-level descriptive stratification.
Region-stratified AESI reporting	Abemaciclib	United States	VTE/thrombotic events	206/11,303	1.8%	Region-stratified reporting context	Describes FAERS reporting composition for descriptive context.	Report-level descriptive stratification.
Region-stratified AESI reporting	Palbociclib	United States	VTE/thrombotic events	939/65,583	1.4%	Region-stratified reporting context	Describes FAERS reporting composition for descriptive context.	Report-level descriptive stratification.
Region-stratified AESI reporting	Ribociclib	United States	VTE/thrombotic events	74/7,358	1.0%	Region-stratified reporting context	Describes FAERS reporting composition for descriptive context.	Report-level descriptive stratification.
Region-stratified AESI reporting	Abemaciclib	China/region	ILD/pneumonitis	16/756	2.1%	Region-stratified reporting context	Describes FAERS reporting composition for descriptive context.	Report-level descriptive stratification.
Region-stratified AESI reporting	Palbociclib	China/region	ILD/pneumonitis	4/709	0.6%	Region-stratified reporting context	Describes FAERS reporting composition for descriptive context.	Report-level descriptive stratification.
Region-stratified AESI reporting	Ribociclib	China/region	ILD/pneumonitis	3/231	1.3%	Region-stratified reporting context	Describes FAERS reporting composition for descriptive context.	Report-level descriptive stratification.
Region-stratified AESI reporting	Abemaciclib	Europe	ILD/pneumonitis	69/1,272	5.4%	Region-stratified reporting context	Describes FAERS reporting composition for descriptive context.	Report-level descriptive stratification.
Region-stratified AESI reporting	Palbociclib	Europe	ILD/pneumonitis	73/2,779	2.6%	Region-stratified reporting context	Describes FAERS reporting composition for descriptive context.	Report-level descriptive stratification.
Region-stratified AESI reporting	Ribociclib	Europe	ILD/pneumonitis	89/3,533	2.5%	Region-stratified reporting context	Describes FAERS reporting composition for descriptive context.	Report-level descriptive stratification.
Region-stratified AESI reporting	Abemaciclib	Japan	ILD/pneumonitis	162/783	20.7%	Region-stratified reporting context	Describes FAERS reporting composition	Report-level descriptive stratification.

Evidence layer	Agent or drug	Search terms or stratum	AESI or role	Reports or capture	Percent or relevance	Analysis role	Interpretation boundary	Notes
							for descriptive context.	
Region-stratified AESI reporting	Palbociclib	Japan	ILD/pneumonitis	55/1,190	4.6%	Region-stratified reporting context	Describes FAERS reporting composition for descriptive context.	Report-level descriptive stratification.
Region-stratified AESI reporting	Ribociclib	Japan	ILD/pneumonitis	1/6	16.7%	Region-stratified reporting context	Describes FAERS reporting composition for descriptive context.	Report-level descriptive stratification.
Region-stratified AESI reporting	Palbociclib	Missing	ILD/pneumonitis	0/1	0.0%	Region-stratified reporting context	Describes FAERS reporting composition for descriptive context.	Report-level descriptive stratification.
Region-stratified AESI reporting	Ribociclib	Missing	ILD/pneumonitis	1/1	100.0%	Region-stratified reporting context	Describes FAERS reporting composition for descriptive context.	Report-level descriptive stratification.
Region-stratified AESI reporting	Abemaciclib	Other	ILD/pneumonitis	29/1,059	2.7%	Region-stratified reporting context	Describes FAERS reporting composition for descriptive context.	Report-level descriptive stratification.
Region-stratified AESI reporting	Palbociclib	Other	ILD/pneumonitis	45/7,653	0.6%	Region-stratified reporting context	Describes FAERS reporting composition for descriptive context.	Report-level descriptive stratification.
Region-stratified AESI reporting	Ribociclib	Other	ILD/pneumonitis	90/7,449	1.2%	Region-stratified reporting context	Describes FAERS reporting composition for descriptive context.	Report-level descriptive stratification.
Region-stratified AESI reporting	Abemaciclib	Other Asia	ILD/pneumonitis	6/157	3.8%	Region-stratified reporting context	Describes FAERS reporting composition for descriptive context.	Report-level descriptive stratification.
Region-stratified AESI reporting	Palbociclib	Other Asia	ILD/pneumonitis	20/2,767	0.7%	Region-stratified reporting context	Describes FAERS reporting composition for descriptive context.	Report-level descriptive stratification.
Region-stratified AESI reporting	Ribociclib	Other Asia	ILD/pneumonitis	19/1,181	1.6%	Region-stratified reporting context	Describes FAERS reporting composition for descriptive context.	Report-level descriptive stratification.
Region-stratified AESI reporting	Abemaciclib	United States	ILD/pneumonitis	172/11,303	1.5%	Region-stratified reporting context	Describes FAERS reporting composition for descriptive context.	Report-level descriptive stratification.
Region-stratified AESI reporting	Palbociclib	United States	ILD/pneumonitis	289/65,583	0.4%	Region-stratified reporting context	Describes FAERS reporting composition for descriptive context.	Report-level descriptive stratification.
Region-stratified AESI reporting	Ribociclib	United States	ILD/pneumonitis	86/7,358	1.2%	Region-stratified reporting context	Describes FAERS reporting composition for descriptive context.	Report-level descriptive stratification.
Region-stratified AESI reporting	Abemaciclib	China/region	Infection/febrile events	15/756	2.0%	Region-stratified reporting context	Describes FAERS reporting composition for descriptive context.	Report-level descriptive stratification.
Region-stratified AESI reporting	Palbociclib	China/region	Infection/febrile events	4/709	0.6%	Region-stratified reporting context	Describes FAERS reporting composition for descriptive context.	Report-level descriptive stratification.
Region-stratified AESI reporting	Ribociclib	China/region	Infection/febrile events	15/231	6.5%	Region-stratified reporting context	Describes FAERS reporting composition for descriptive context.	Report-level descriptive stratification.
Region-stratified AESI	Abemaciclib	Europe	Infection/febrile events	89/1,272	7.0%	Region-stratified	Describes FAERS	Report-level descriptive

Evidence layer	Agent or drug	Search terms or stratum	AESI or role	Reports or capture	Percent or relevance	Analysis role	Interpretation boundary	Notes
reporting						reporting context	reporting composition for descriptive context.	stratification.
Region-stratified AESI reporting	Palbociclib	Europe	Infection/febrile events	226/2,779	8.1%	Region-stratified reporting context	Describes FAERS reporting composition for descriptive context.	Report-level descriptive stratification.
Region-stratified AESI reporting	Ribociclib	Europe	Infection/febrile events	346/3,533	9.8%	Region-stratified reporting context	Describes FAERS reporting composition for descriptive context.	Report-level descriptive stratification.
Region-stratified AESI reporting	Abemaciclib	Japan	Infection/febrile events	44/783	5.6%	Region-stratified reporting context	Describes FAERS reporting composition for descriptive context.	Report-level descriptive stratification.
Region-stratified AESI reporting	Palbociclib	Japan	Infection/febrile events	67/1,190	5.6%	Region-stratified reporting context	Describes FAERS reporting composition for descriptive context.	Report-level descriptive stratification.
Region-stratified AESI reporting	Ribociclib	Japan	Infection/febrile events	0/6	0.0%	Region-stratified reporting context	Describes FAERS reporting composition for descriptive context.	Report-level descriptive stratification.
Region-stratified AESI reporting	Palbociclib	Missing	Infection/febrile events	0/1	0.0%	Region-stratified reporting context	Describes FAERS reporting composition for descriptive context.	Report-level descriptive stratification.
Region-stratified AESI reporting	Ribociclib	Missing	Infection/febrile events	0/1	0.0%	Region-stratified reporting context	Describes FAERS reporting composition for descriptive context.	Report-level descriptive stratification.
Region-stratified AESI reporting	Abemaciclib	Other	Infection/febrile events	64/1,059	6.0%	Region-stratified reporting context	Describes FAERS reporting composition for descriptive context.	Report-level descriptive stratification.
Region-stratified AESI reporting	Palbociclib	Other	Infection/febrile events	297/7,653	3.9%	Region-stratified reporting context	Describes FAERS reporting composition for descriptive context.	Report-level descriptive stratification.
Region-stratified AESI reporting	Ribociclib	Other	Infection/febrile events	758/7,449	10.2%	Region-stratified reporting context	Describes FAERS reporting composition for descriptive context.	Report-level descriptive stratification.
Region-stratified AESI reporting	Abemaciclib	Other Asia	Infection/febrile events	12/157	7.6%	Region-stratified reporting context	Describes FAERS reporting composition for descriptive context.	Report-level descriptive stratification.
Region-stratified AESI reporting	Palbociclib	Other Asia	Infection/febrile events	295/2,767	10.7%	Region-stratified reporting context	Describes FAERS reporting composition for descriptive context.	Report-level descriptive stratification.
Region-stratified AESI reporting	Ribociclib	Other Asia	Infection/febrile events	113/1,181	9.6%	Region-stratified reporting context	Describes FAERS reporting composition for descriptive context.	Report-level descriptive stratification.
Region-stratified AESI reporting	Abemaciclib	United States	Infection/febrile events	412/11,303	3.6%	Region-stratified reporting context	Describes FAERS reporting composition for descriptive context.	Report-level descriptive stratification.
Region-stratified AESI reporting	Palbociclib	United States	Infection/febrile events	3,928/65,583	6.0%	Region-stratified reporting context	Describes FAERS reporting composition for descriptive context.	Report-level descriptive stratification.
Region-stratified AESI reporting	Ribociclib	United States	Infection/febrile events	293/7,358	4.0%	Region-stratified reporting context	Describes FAERS reporting composition for descriptive context.	Report-level descriptive stratification.

Evidence layer	Agent or drug	Search terms or stratum	AESI or role	Reports or capture	Percent or relevance	Analysis role	Interpretation boundary	Notes
Region-stratified AESI reporting	Abemaciclib	China/region	Renal/creatinine abnormality	48/756	6.3%	Region-stratified reporting context	Describes FAERS reporting composition for descriptive context.	Report-level descriptive stratification.
Region-stratified AESI reporting	Palbociclib	China/region	Renal/creatinine abnormality	15/709	2.1%	Region-stratified reporting context	Describes FAERS reporting composition for descriptive context.	Report-level descriptive stratification.
Region-stratified AESI reporting	Ribociclib	China/region	Renal/creatinine abnormality	9/231	3.9%	Region-stratified reporting context	Describes FAERS reporting composition for descriptive context.	Report-level descriptive stratification.
Region-stratified AESI reporting	Abemaciclib	Europe	Renal/creatinine abnormality	181/1,272	14.2%	Region-stratified reporting context	Describes FAERS reporting composition for descriptive context.	Report-level descriptive stratification.
Region-stratified AESI reporting	Palbociclib	Europe	Renal/creatinine abnormality	207/2,779	7.4%	Region-stratified reporting context	Describes FAERS reporting composition for descriptive context.	Report-level descriptive stratification.
Region-stratified AESI reporting	Ribociclib	Europe	Renal/creatinine abnormality	329/3,533	9.3%	Region-stratified reporting context	Describes FAERS reporting composition for descriptive context.	Report-level descriptive stratification.
Region-stratified AESI reporting	Abemaciclib	Japan	Renal/creatinine abnormality	59/783	7.5%	Region-stratified reporting context	Describes FAERS reporting composition for descriptive context.	Report-level descriptive stratification.
Region-stratified AESI reporting	Palbociclib	Japan	Renal/creatinine abnormality	130/1,190	10.9%	Region-stratified reporting context	Describes FAERS reporting composition for descriptive context.	Report-level descriptive stratification.
Region-stratified AESI reporting	Ribociclib	Japan	Renal/creatinine abnormality	1/6	16.7%	Region-stratified reporting context	Describes FAERS reporting composition for descriptive context.	Report-level descriptive stratification.
Region-stratified AESI reporting	Palbociclib	Missing	Renal/creatinine abnormality	0/1	0.0%	Region-stratified reporting context	Describes FAERS reporting composition for descriptive context.	Report-level descriptive stratification.
Region-stratified AESI reporting	Ribociclib	Missing	Renal/creatinine abnormality	0/1	0.0%	Region-stratified reporting context	Describes FAERS reporting composition for descriptive context.	Report-level descriptive stratification.
Region-stratified AESI reporting	Abemaciclib	Other	Renal/creatinine abnormality	98/1,059	9.3%	Region-stratified reporting context	Describes FAERS reporting composition for descriptive context.	Report-level descriptive stratification.
Region-stratified AESI reporting	Palbociclib	Other	Renal/creatinine abnormality	223/7,653	2.9%	Region-stratified reporting context	Describes FAERS reporting composition for descriptive context.	Report-level descriptive stratification.
Region-stratified AESI reporting	Ribociclib	Other	Renal/creatinine abnormality	469/7,449	6.3%	Region-stratified reporting context	Describes FAERS reporting composition for descriptive context.	Report-level descriptive stratification.
Region-stratified AESI reporting	Abemaciclib	Other Asia	Renal/creatinine abnormality	11/157	7.0%	Region-stratified reporting context	Describes FAERS reporting composition for descriptive context.	Report-level descriptive stratification.
Region-stratified AESI reporting	Palbociclib	Other Asia	Renal/creatinine abnormality	189/2,767	6.8%	Region-stratified reporting context	Describes FAERS reporting composition for descriptive context.	Report-level descriptive stratification.
Region-stratified AESI reporting	Ribociclib	Other Asia	Renal/creatinine abnormality	86/1,181	7.3%	Region-stratified reporting context	Describes FAERS reporting composition	Report-level descriptive stratification.

Evidence layer	Agent or drug	Search terms or stratum	AESI or role	Reports or capture	Percent or relevance	Analysis role	Interpretation boundary	Notes
							for descriptive context.	
Region-stratified AESI reporting	Abemaciclib	United States	Renal/creatinine abnormality	471/11,303	4.2%	Region-stratified reporting context	Describes FAERS reporting composition for descriptive context.	Report-level descriptive stratification.
Region-stratified AESI reporting	Palbociclib	United States	Renal/creatinine abnormality	1,133/65,583	1.7%	Region-stratified reporting context	Describes FAERS reporting composition for descriptive context.	Report-level descriptive stratification.
Region-stratified AESI reporting	Ribociclib	United States	Renal/creatinine abnormality	243/7,358	3.3%	Region-stratified reporting context	Describes FAERS reporting composition for descriptive context.	Report-level descriptive stratification.

Table S2. Intraclass adjusted reporting odds models and abemaciclib-versus-ribociclib contrasts by AESI.

Rows were rebuilt from intraclass adjusted OR source CSV and stage-1 abemaciclib-versus-ribociclib source sheet.

AESI	Contrast layer	Comparison	Reference	OR (95% CI)	p value	Compared-drug events	Compared-drug reports	Reference-drug events	Reference-drug reports	Adjustment or boundary
Hematologic toxicity	Palbociclib-reference intraclass model	Abemaciclib vs Palbociclib	Palbociclib	0.52 (0.49 to 0.56)	p<0.001	2,112	15,330	19,685	80,682	Adjusted for age group, sex group, report year, reporter region, and reporter type when convergence permitted.
Hematologic toxicity	Palbociclib-reference intraclass model	Ribociclib vs Palbociclib	Palbociclib	0.71 (0.67 to 0.76)	p<0.001	4,181	19,759	19,685	80,682	Adjusted for age group, sex group, report year, reporter region, and reporter type when convergence permitted.
Gastrointestinal toxicity	Palbociclib-reference intraclass model	Abemaciclib vs Palbociclib	Palbociclib	3.14 (2.98 to 3.31)	p<0.001	5,780	15,330	14,412	80,682	Adjusted for age group, sex group, report year, reporter region, and reporter type when convergence permitted.
Gastrointestinal toxicity	Palbociclib-reference intraclass model	Ribociclib vs Palbociclib	Palbociclib	1.58 (1.48 to 1.69)	p<0.001	3,924	19,759	14,412	80,682	Adjusted for age group, sex group, report year, reporter region, and reporter type when convergence permitted.
Hepatobiliary toxicity	Palbociclib-reference intraclass model	Abemaciclib vs Palbociclib	Palbociclib	2.26 (2.04 to 2.52)	p<0.001	1,123	15,330	2,054	80,682	Adjusted for age group, sex group, report year, reporter region, and reporter type when convergence permitted.
Hepatobiliary toxicity	Palbociclib-reference intraclass model	Ribociclib vs Palbociclib	Palbociclib	2.86 (2.6 to 3.14)	p<0.001	2,678	19,759	2,054	80,682	Adjusted for age group, sex group,

AESI	Contrast layer	Comparison	Reference	OR (95% CI)	p value	Compared-drug events	Compared-drug reports	Reference-drug events	Reference-drug reports	Adjustment or boundary
										report year, reporter region, and reporter type when convergence permitted.
QT/cardiac toxicity	Palbociclib-reference intraclass model	Abemaciclib vs Palbociclib	Palbociclib	1.33 (0.97 to 1.82)	p=0.075	71	15,330	305	80,682	Adjusted for age group, sex group, report year, reporter region, and reporter type when convergence permitted.
QT/cardiac toxicity	Palbociclib-reference intraclass model	Ribociclib vs Palbociclib	Palbociclib	8.66 (7.08 to 10.59)	p<0.001	949	19,759	305	80,682	Adjusted for age group, sex group, report year, reporter region, and reporter type when convergence permitted.
VTE/thrombotic events	Palbociclib-reference intraclass model	Abemaciclib vs Palbociclib	Palbociclib	1.55 (1.34 to 1.8)	p<0.001	515	15,330	1,432	80,682	Adjusted for age group, sex group, report year, reporter region, and reporter type when convergence permitted.
VTE/thrombotic events	Palbociclib-reference intraclass model	Ribociclib vs Palbociclib	Palbociclib	1.01 (0.86 to 1.18)	p=0.886	474	19,759	1,432	80,682	Adjusted for age group, sex group, report year, reporter region, and reporter type when convergence permitted.
ILD/pneumonitis	Palbociclib-reference intraclass model	Abemaciclib vs Palbociclib	Palbociclib	4.33 (3.6 to 5.21)	p<0.001	454	15,330	486	80,682	Adjusted for age group, sex group, report year, reporter region, and reporter type when convergence permitted.
ILD/pneumonitis	Palbociclib-reference intraclass model	Ribociclib vs Palbociclib	Palbociclib	2.1 (1.68 to 2.62)	p<0.001	289	19,759	486	80,682	Adjusted for age group, sex group, report year, reporter region, and reporter type when convergence permitted.
Infection/febrile events	Palbociclib-reference intraclass model	Abemaciclib vs Palbociclib	Palbociclib	0.73 (0.66 to 0.81)	p<0.001	636	15,330	4,817	80,682	Adjusted for age group, sex group, report year, reporter region, and reporter type when convergence permitted.
Infection/febrile events	Palbociclib-reference intraclass model	Ribociclib vs Palbociclib	Palbociclib	1.52 (1.38 to 1.66)	p<0.001	1,525	19,759	4,817	80,682	Adjusted for age group, sex group, report year, reporter region, and reporter

AESI	Contrast layer	Comparison	Reference	OR (95% CI)	p value	Compared-drug events	Compared-drug reports	Reference-drug events	Reference-drug reports	Adjustment or boundary
										type when convergence permitted.
Renal/creatinine abnormality	Palbociclib-reference intraclass model	Abemaciclib vs Palbociclib	Palbociclib	2.52 (2.25 to 2.82)	p<0.001	868	15,330	1,897	80,682	Adjusted for age group, sex group, report year, reporter region, and reporter type when convergence permitted.
Renal/creatinine abnormality	Palbociclib-reference intraclass model	Ribociclib vs Palbociclib	Palbociclib	1.82 (1.62 to 2.04)	p<0.001	1,137	19,759	1,897	80,682	Adjusted for age group, sex group, report year, reporter region, and reporter type when convergence permitted.
Hematologic toxicity	Abemaciclib-versus-ribociclib contrast	Abemaciclib vs Ribociclib	Ribociclib	0.62 (0.57 to 0.66)	p<0.001	2,112	15,330	4,181	19,759	age group, sex group, report year, reporter region, reporter type; adjusted reporting odds within submitted FAERS reports.
Gastrointestinal toxicity	Abemaciclib-versus-ribociclib contrast	Abemaciclib vs Ribociclib	Ribociclib	2.08 (1.97 to 2.21)	p<0.001	5,780	15,330	3,924	19,759	age group, sex group, report year, reporter region, reporter type; adjusted reporting odds within submitted FAERS reports.
Hepatobiliary toxicity	Abemaciclib-versus-ribociclib contrast	Abemaciclib vs Ribociclib	Ribociclib	0.58 (0.53 to 0.63)	p<0.001	1,123	15,330	2,678	19,759	age group, sex group, report year, reporter region, reporter type; adjusted reporting odds within submitted FAERS reports.
QT/cardiac toxicity	Abemaciclib-versus-ribociclib contrast	Abemaciclib vs Ribociclib	Ribociclib	0.1 (0.08 to 0.13)	p<0.001	71	15,330	949	19,759	age group, sex group, report year, reporter region, reporter type; adjusted reporting odds within submitted FAERS reports.
VTE/thrombotic events	Abemaciclib-versus-ribociclib contrast	Abemaciclib vs Ribociclib	Ribociclib	2.61 (2.23 to 3.05)	p<0.001	515	15,330	474	19,759	age group, sex group, report year, reporter region, reporter type; adjusted reporting odds within submitted FAERS reports.
ILD/pneumonitis	Abemaciclib-versus-	Abemaciclib vs	Ribociclib	1.93 (1.59 to 2.35)	p<0.001	454	15,330	289	19,759	age group, sex

AESI	Contrast layer	Comparison	Reference	OR (95% CI)	p value	Compared-drug events	Compared-drug reports	Reference-drug events	Reference-drug reports	Adjustment or boundary
	ribociclib contrast	Ribociclib								group, report year, reporter region, reporter type; adjusted reporting odds within submitted FAERS reports.
Infection/febrile events	Abemaciclib-versus-ribociclib contrast	Abemaciclib vs Ribociclib	Ribociclib	0.63 (0.56 to 0.71)	p<0.001	636	15,330	1,525	19,759	age group, sex group, report year, reporter region, reporter type; adjusted reporting odds within submitted FAERS reports.
Renal/creatinine abnormality	Abemaciclib-versus-ribociclib contrast	Abemaciclib vs Ribociclib	Ribociclib	1.41 (1.26 to 1.57)	p<0.001	868	15,330	1,137	19,759	age group, sex group, report year, reporter region, reporter type; adjusted reporting odds within submitted FAERS reports.

Table S3. Reported TTO availability, summary statistics, and reported TTO boundary by drug and AESI.

TTO summaries include reports with valid non-negative TTO and describe reported timing among submitted reports.

Drug	AESI	Drug-AESI reports	Valid TTO reports, n (%)	Median TTO, days (IQR)	Interpretation boundary
Palbociclib	Hematologic toxicity	19,685	3,500 (17.8%)	50 (18 to 230)	TTO distributions include only reports with valid non-negative TTO; denominator is report-level drug-AESI reports.
Palbociclib	Gastrointestinal toxicity	14,412	2,140 (14.8%)	53.5 (16 to 218)	TTO distributions include only reports with valid non-negative TTO; denominator is report-level drug-AESI reports.
Palbociclib	Hepatobiliary toxicity	2,054	521 (25.4%)	70 (24 to 320)	TTO distributions include only reports with valid non-negative TTO; denominator is report-level drug-AESI reports.
Palbociclib	QT/cardiac toxicity	305	77 (25.2%)	76 (18 to 351)	TTO distributions include only reports with valid non-negative TTO; denominator is report-level drug-AESI reports.
Palbociclib	VTE/thrombotic events	1,432	377 (26.3%)	103 (41 to 363)	TTO distributions include only reports with valid non-negative TTO; denominator is report-level drug-AESI reports.
Palbociclib	ILD/pneumonitis	486	132 (27.2%)	138 (41.2 to 317.5)	TTO distributions include only reports with valid non-negative TTO; denominator is report-level

Drug	AESI	Drug-AESI reports	Valid TTO reports, n (%)	Median TTO, days (IQR)	Interpretation boundary
					drug-AESI reports.
Palbociclib	Infection/febrile events	4,817	1,002 (20.8%)	101.5 (24 to 382.8)	TTO distributions include only reports with valid non-negative TTO; denominator is report-level drug-AESI reports.
Palbociclib	Renal/creatinine abnormality	1,897	476 (25.1%)	70 (17 to 348)	TTO distributions include only reports with valid non-negative TTO; denominator is report-level drug-AESI reports.
Abemaciclib	Hematologic toxicity	2,112	753 (35.7%)	29 (14 to 92)	TTO distributions include only reports with valid non-negative TTO; denominator is report-level drug-AESI reports.
Abemaciclib	Gastrointestinal toxicity	5,780	953 (16.5%)	25 (7 to 77)	TTO distributions include only reports with valid non-negative TTO; denominator is report-level drug-AESI reports.
Abemaciclib	Hepatobiliary toxicity	1,123	337 (30.0%)	41 (17 to 84)	TTO distributions include only reports with valid non-negative TTO; denominator is report-level drug-AESI reports.
Abemaciclib	QT/cardiac toxicity	71	20 (28.2%)	76.5 (14 to 119.5)	TTO distributions include only reports with valid non-negative TTO; denominator is report-level drug-AESI reports.
Abemaciclib	VTE/thrombotic events	515	119 (23.1%)	102 (31 to 275.5)	TTO distributions include only reports with valid non-negative TTO; denominator is report-level drug-AESI reports.
Abemaciclib	ILD/pneumonitis	454	143 (31.5%)	70 (28 to 223)	TTO distributions include only reports with valid non-negative TTO; denominator is report-level drug-AESI reports.
Abemaciclib	Infection/febrile events	636	172 (27.0%)	37.5 (12.8 to 182)	TTO distributions include only reports with valid non-negative TTO; denominator is report-level drug-AESI reports.
Abemaciclib	Renal/creatinine abnormality	868	225 (25.9%)	17 (9 to 74)	TTO distributions include only reports with valid non-negative TTO; denominator is report-level drug-AESI reports.
Ribociclib	Hematologic toxicity	4,181	1,551 (37.1%)	28 (14 to 108.5)	TTO distributions include only reports with valid non-negative TTO; denominator is report-level drug-AESI reports.
Ribociclib	Gastrointestinal toxicity	3,924	1,055 (26.9%)	30 (12 to 145.5)	TTO distributions include only reports with valid non-negative TTO; denominator is report-level drug-AESI reports.

Drug	AESI	Drug-AESI reports	Valid TTO reports, n (%)	Median TTO, days (IQR)	Interpretation boundary
Ribociclib	Hepatobiliary toxicity	2,678	871 (32.5%)	58 (20 to 140.5)	TTO distributions include only reports with valid non-negative TTO; denominator is report-level drug-AESI reports.
Ribociclib	QT/cardiac toxicity	949	299 (31.5%)	24 (13 to 74)	TTO distributions include only reports with valid non-negative TTO; denominator is report-level drug-AESI reports.
Ribociclib	VTE/thrombotic events	474	197 (41.6%)	76 (25 to 340)	TTO distributions include only reports with valid non-negative TTO; denominator is report-level drug-AESI reports.
Ribociclib	ILD/pneumonitis	289	114 (39.4%)	82 (27.2 to 192.2)	TTO distributions include only reports with valid non-negative TTO; denominator is report-level drug-AESI reports.
Ribociclib	Infection/febrile events	1,525	631 (41.4%)	49 (15 to 228)	TTO distributions include only reports with valid non-negative TTO; denominator is report-level drug-AESI reports.
Ribociclib	Renal/creatinine abnormality	1,137	429 (37.7%)	31 (13 to 177)	TTO distributions include only reports with valid non-negative TTO; denominator is report-level drug-AESI reports.

Table S4. ML temporal validation, sensitivity performance, and calibration.

Primary and sensitivity model rows use one shared header.

Model layer	AESI	Model	Includes TTO	Validation reports	Validation events	Validation prevalence	AUROC	AUPRC	AUPRC lift	Brier score	Calibration intercept	Calibration slope
Primary temporal validation	Hematologic toxicity	Post-report elastic-net logistic	Yes	39,190	9,144	23.3%	0.691	0.386	1.65	0.244	-1.353	1.107
baseline-only sensitivity	Hematologic toxicity	Baseline-only elastic-net logistic	No	39,190	9,144	23.3%	0.689	0.373	1.6	0.245	-1.349	1.14
random forest sensitivity	Hematologic toxicity	Post-report random forest	Yes	39,190	9,144	23.3%	0.689	0.383	1.64	0.167	-0.259	1.314
Primary temporal validation	Gastrointestinal toxicity	Post-report elastic-net logistic	Yes	39,190	9,247	23.6%	0.719	0.442	1.87	0.239	-1.343	1.337
baseline-only sensitivity	Gastrointestinal toxicity	Baseline-only elastic-net logistic	No	39,190	9,247	23.6%	0.716	0.437	1.85	0.24	-1.348	1.312
random forest sensitivity	Gastrointestinal toxicity	Post-report random forest	Yes	39,190	9,247	23.6%	0.733	0.458	1.94	0.157	-0.117	1.009
Primary temporal validation	Hepatobiliary toxicity	Post-report elastic-net logistic	Yes	39,190	2,961	7.6%	0.783	0.205	2.71	0.228	-2.714	1.062
baseline-only sensitivity	Hepatobiliary toxicity	Baseline-only elastic-net logistic	No	39,190	2,961	7.6%	0.781	0.201	2.66	0.229	-2.697	1.099
random forest sensitivity	Hepatobiliary toxicity	Post-report random forest	Yes	39,190	2,961	7.6%	0.782	0.211	2.79	0.09	-1.325	0.915
Primary temporal validation	QT/cardiac toxicity	Post-report elastic-net logistic	Yes	39,190	648	1.7%	0.822	0.072	4.33	0.227	-4.315	1.253

Model layer	AESI	Model	Includes TTO	Validation reports	Validation events	Validation prevalence	AUROC	AUPRC	AUPRC lift	Brier score	Calibration intercept	Calibration slope
baseline-only sensitivity	QT/cardiac toxicity	Baseline-only elastic-net logistic	No	39,190	648	1.7%	0.822	0.07	4.21	0.225	-4.351	1.163
random forest sensitivity	QT/cardiac toxicity	Post-report random forest	Yes	39,190	648	1.7%	0.811	0.058	3.53	0.106	-3.395	0.8
Primary temporal validation	VTE/thrombotic events	Post-report elastic-net logistic	Yes	39,190	937	2.4%	0.683	0.049	2.04	0.244	-3.777	1.139
baseline-only sensitivity	VTE/thrombotic events	Baseline-only elastic-net logistic	No	39,190	937	2.4%	0.684	0.049	2.04	0.246	-3.785	1.157
random forest sensitivity	VTE/thrombotic events	Post-report random forest	Yes	39,190	937	2.4%	0.675	0.051	2.15	0.062	-2.392	0.77
Primary temporal validation	ILD/pneumonitis	Post-report elastic-net logistic	Yes	39,190	560	1.4%	0.732	0.045	3.18	0.22	-4.267	1.117
baseline-only sensitivity	ILD/pneumonitis	Baseline-only elastic-net logistic	No	39,190	560	1.4%	0.73	0.045	3.18	0.22	-4.228	1.254
random forest sensitivity	ILD/pneumonitis	Post-report random forest	Yes	39,190	560	1.4%	0.747	0.078	5.47	0.069	-3.126	0.937
Primary temporal validation	Infection/febrile events	Post-report elastic-net logistic	Yes	39,190	2,596	6.6%	0.68	0.12	1.81	0.231	-2.618	1.471
baseline-only sensitivity	Infection/febrile events	Baseline-only elastic-net logistic	No	39,190	2,596	6.6%	0.68	0.12	1.81	0.229	-2.611	1.499
random forest sensitivity	Infection/febrile events	Post-report random forest	Yes	39,190	2,596	6.6%	0.671	0.137	2.07	0.079	-1.264	1.039
Primary temporal validation	Renal/creatinine abnormality	Post-report elastic-net logistic	Yes	39,190	1,816	4.6%	0.71	0.106	2.29	0.258	-3.237	1
baseline-only sensitivity	Renal/creatinine abnormality	Baseline-only elastic-net logistic	No	39,190	1,816	4.6%	0.709	0.102	2.2	0.261	-3.259	0.963
random forest sensitivity	Renal/creatinine abnormality	Post-report random forest	Yes	39,190	1,816	4.6%	0.712	0.115	2.47	0.079	-1.808	0.851

Table S5. Top k enrichment of ML signal prioritization models.

Top-k metrics describe report-level enrichment for pharmacovigilance triage.

AESI	Validation prevalence	PPV@5%	recall@5%	enrichment@5%	PPV@10%	recall@10%	enrichment@10%	PPV@20%	recall@20%	enrichment@20%
Gastrointestinal toxicity	23.6%	58.3%	12.4%	2.47	54.4%	23.1%	2.31	46.1%	39.1%	1.95
Hematologic toxicity	23.3%	47.7%	10.2%	2.04	44.1%	18.9%	1.89	41.7%	35.8%	1.79
Hepatobiliary toxicity	7.6%	26.5%	17.5%	3.5	24.1%	31.9%	3.19	20.2%	53.5%	2.68
ILD/pneumonitis	1.4%	6.4%	22.3%	4.46	4.8%	33.8%	3.38	3.5%	49.5%	2.47
Infection/febrile events	6.6%	14.2%	10.7%	2.14	15.5%	23.4%	2.34	13.7%	41.3%	2.06
QT/cardiac toxicity	1.7%	9.2%	27.9%	5.58	7.5%	45.1%	4.51	5.6%	68.2%	3.41
Renal/creatinine abnormality	4.6%	13.7%	14.8%	2.96	12.1%	26.0%	2.6	10.2%	43.9%	2.2
VTE/thrombotic events	2.4%	6.3%	13.1%	2.62	6.3%	26.4%	2.64	5.2%	43.5%	2.18

Table S6. Incremental model performance, incremental differences, and practical interpretation by AESI.

Incremental performance rows use one shared model-step header.

AESI	Model step	Model	Includes TTO	AUROC	AUPRC	AUPRC lift	Brier score	Delta AUPRC model 2 vs model 1	Delta AUPRC full vs model 2	Delta AUROC model 2 vs model 1	Delta AUROC full vs model 2	Top feature groups	Interpretation boundary
Gastrointestinal toxicity	Model 1: drug only	Drug-only elastic-net logistic	No	0.614	0.334	1.41	0.25	0.074	0.034	0.083	0.022	reporter country/region; concomitant medication group; reporter type	ML priority is a FAERS report-review triage score for pharmacovigilance prioritization.
Gastrointestinal toxicity	Model 2: reporting-demographic	Reporting-demographic elastic-net logistic	No	0.697	0.408	1.73	0.241	0.074	0.034	0.083	0.022	reporter country/region; concomitant medication group; reporter type	ML priority is a FAERS report-review triage score for pharmacovigilance prioritization.
Gastrointestinal toxicity	Model 3: full post-report	Post-report elastic-net logistic	Yes	0.719	0.442	1.87	0.239	0.074	0.034	0.083	0.022	reporter country/region; concomitant medication group; reporter type	ML priority is a FAERS report-review triage score for pharmacovigilance prioritization.
Hematologic toxicity	Model 1: drug only	Drug-only elastic-net logistic	No	0.551	0.296	1.27	0.245	0.03	0.06	0.074	0.066	concomitant medication group; reporter country/region; reported TTO group	ML priority is a FAERS report-review triage score for pharmacovigilance prioritization.
Hematologic toxicity	Model 2: reporting-demographic	Reporting-demographic elastic-net logistic	No	0.625	0.326	1.4	0.234	0.03	0.06	0.074	0.066	concomitant medication group; reporter country/region; reported TTO group	ML priority is a FAERS report-review triage score for pharmacovigilance prioritization.
Hematologic toxicity	Model 3: full post-report	Post-report elastic-net logistic	Yes	0.691	0.386	1.65	0.244	0.03	0.06	0.074	0.066	concomitant medication group; reporter country/region; reported TTO group	ML priority is a FAERS report-review triage score for pharmacovigilance prioritization.
Hepatobiliary toxicity	Model 1: drug only	Drug-only elastic-net logistic	No	0.67	0.119	1.58	0.251	0.08	0.006	0.107	0.005	reporter country/region; drug; reporter type	ML priority is a FAERS report-review triage score for pharmacovigilance prioritization.
Hepatobiliary toxicity	Model 2: reporting-demographic	Reporting-demographic elastic-net logistic	No	0.777	0.199	2.63	0.23	0.08	0.006	0.107	0.005	reporter country/region; drug; reporter type	ML priority is a FAERS report-review triage score for pharmacovigilance prioritization.
Hepatobiliary toxicity	Model 3: full post-report	Post-report elastic-net logistic	Yes	0.783	0.205	2.71	0.228	0.08	0.006	0.107	0.005	reporter country/region; drug; reporter type	ML priority is a FAERS report-review triage score for pharmacovigilance prioritization.
ILD/pneumonitis	Model 1: drug only	Drug-only elastic-net logistic	No	0.64	0.021	1.49	0.254	0.023	0.001	0.082	0.011	reporter country/region; reporter type; drug	ML priority is a FAERS report-review triage score for pharmacovigilance prioritization.

AESI	Model step	Model	Includes TTO	AUROC	AUPRC	AUPRC lift	Brier score	Delta AUPRC model 2 vs model 1	Delta AUPRC full vs model 2	Delta AUROC model 2 vs model 1	Delta AUROC full vs model 2	Top feature groups	Interpretation boundary
ILD/pneumonitis	Model 2: reporting-demographic	Reporting-demographic elastic-net logistic	No	0.721	0.045	3.12	0.225	0.023	0.001	0.082	0.011	reporter country/region; reporter type; drug	ML priority is a FAERS report-review triage score for pharmacovigilance prioritization.
ILD/pneumonitis	Model 3: full post-report	Post-report elastic-net logistic	Yes	0.732	0.045	3.18	0.22	0.023	0.001	0.082	0.011	reporter country/region; reporter type; drug	ML priority is a FAERS report-review triage score for pharmacovigilance prioritization.
Infection/febrile events	Model 1: drug only	Drug-only elastic-net logistic	No	0.555	0.093	1.41	0.244	0.002	0.024	0.071	0.054	concomitant medication group; reporter country/region; sex	ML priority is a FAERS report-review triage score for pharmacovigilance prioritization.
Infection/febrile events	Model 2: reporting-demographic	Reporting-demographic elastic-net logistic	No	0.626	0.096	1.44	0.235	0.002	0.024	0.071	0.054	concomitant medication group; reporter country/region; sex	ML priority is a FAERS report-review triage score for pharmacovigilance prioritization.
Infection/febrile events	Model 3: full post-report	Post-report elastic-net logistic	Yes	0.68	0.12	1.81	0.231	0.002	0.024	0.071	0.054	concomitant medication group; reporter country/region; sex	ML priority is a FAERS report-review triage score for pharmacovigilance prioritization.
QT/cardiac toxicity	Model 1: drug only	Drug-only elastic-net logistic	No	0.775	0.049	2.98	0.247	0.02	0.002	0.043	0.005	drug; reporter country/region; reporter type	ML priority is a FAERS report-review triage score for pharmacovigilance prioritization.
QT/cardiac toxicity	Model 2: reporting-demographic	Reporting-demographic elastic-net logistic	No	0.817	0.07	4.21	0.228	0.02	0.002	0.043	0.005	drug; reporter country/region; reporter type	ML priority is a FAERS report-review triage score for pharmacovigilance prioritization.
QT/cardiac toxicity	Model 3: full post-report	Post-report elastic-net logistic	Yes	0.822	0.072	4.33	0.227	0.02	0.002	0.043	0.005	drug; reporter country/region; reporter type	ML priority is a FAERS report-review triage score for pharmacovigilance prioritization.
Renal/creatinine abnormality	Model 1: drug only	Drug-only elastic-net logistic	No	0.591	0.066	1.43	0.252	0.024	0.015	0.091	0.029	reporter country/region; concomitant medication group; drug	ML priority is a FAERS report-review triage score for pharmacovigilance prioritization.
Renal/creatinine abnormality	Model 2: reporting-demographic	Reporting-demographic elastic-net logistic	No	0.681	0.091	1.96	0.262	0.024	0.015	0.091	0.029	reporter country/region; concomitant medication group; drug	ML priority is a FAERS report-review triage score for pharmacovigilance prioritization.
Renal/creatinine abnormality	Model 3: full post-report	Post-report elastic-net logistic	Yes	0.71	0.106	2.29	0.258	0.024	0.015	0.091	0.029	reporter country/region; concomitant medication group; drug	ML priority is a FAERS report-review triage score for pharmacovigilance prioritization.

AESI	Model step	Model	Includes TTO	AUROC	AUPRC	AUPRC lift	Brier score	Delta AUPRC model 2 vs model 1	Delta AUPRC full vs model 2	Delta AUROC model 2 vs model 1	Delta AUROC full vs model 2	Top feature groups	Interpretation boundary
VTE/thrombotic events	Model 1: drug only	Drug-only elastic-net logistic	No	0.566	0.033	1.4	0.251	0.018	-0.002	0.111	0.006	reporter country/region; concomitant medication group; reporter type	ML priority is a FAERS report-review triage score for pharmacovigilance prioritization.
VTE/thrombotic events	Model 2: reporting-demographic	Reporting-demographic elastic-net logistic	No	0.677	0.051	2.14	0.245	0.018	-0.002	0.111	0.006	reporter country/region; concomitant medication group; reporter type	ML priority is a FAERS report-review triage score for pharmacovigilance prioritization.
VTE/thrombotic events	Model 3: full post-report	Post-report elastic-net logistic	Yes	0.683	0.049	2.04	0.244	0.018	-0.002	0.111	0.006	reporter country/region; concomitant medication group; reporter type	ML priority is a FAERS report-review triage score for pharmacovigilance prioritization.

Table S7. ML threshold metrics, grouped feature contributions, and practical interpretation of priority scores.

Threshold metrics, grouped feature contributions, and interpretation boundaries were converted into a single long table with one shared header.

Evidence layer	AESI	Model	Item	Metric values	Feature contribution	Use or boundary
Threshold metric	Gastrointestinal toxicity	Post-report elastic-net logistic	top5%	threshold=0.753; sensitivity=12.4%; specificity=97.3%; PPV=58.3%; NPV=78.2%; F1=0.204; enrichment=2.47	NA	top-k threshold for FAERS signal-triage workload planning
Threshold metric	Gastrointestinal toxicity	Post-report elastic-net logistic	top10%	threshold=0.715; sensitivity=23.1%; specificity=94.0%; PPV=54.4%; NPV=79.8%; F1=0.324; enrichment=2.31	NA	top-k threshold for FAERS signal-triage workload planning
Threshold metric	Gastrointestinal toxicity	Post-report elastic-net logistic	top20%	threshold=0.643; sensitivity=39.1%; specificity=85.9%; PPV=46.1%; NPV=82.0%; F1=0.423; enrichment=1.95	NA	top-k threshold for FAERS signal-triage workload planning
Threshold metric	Gastrointestinal toxicity	Baseline-only elastic-net logistic	top5%	threshold=0.757; sensitivity=12.3%; specificity=97.3%; PPV=58.1%; NPV=78.2%; F1=0.203; enrichment=2.46	NA	top-k threshold for FAERS signal-triage workload planning
Threshold metric	Gastrointestinal toxicity	Baseline-only elastic-net logistic	top10%	threshold=0.719; sensitivity=23.0%; specificity=94.0%; PPV=54.2%; NPV=79.8%; F1=0.323; enrichment=2.3	NA	top-k threshold for FAERS signal-triage workload planning
Threshold metric	Gastrointestinal toxicity	Baseline-only elastic-net logistic	top20%	threshold=0.642; sensitivity=38.8%; specificity=85.8%; PPV=45.7%; NPV=81.9%;	NA	top-k threshold for FAERS signal-triage workload planning

Evidence layer	AESI	Model	Item	Metric values	Feature contribution	Use or boundary
				F1=0.42; enrichment=1.94		
Threshold metric	Gastrointestinal toxicity	Post-report random forest	top5%	threshold=0.561; sensitivity=13.0%; specificity=97.5%; PPV=61.3%; NPV=78.4%; F1=0.215; enrichment=2.6	NA	top-k threshold for FAERS signal-triage workload planning
Threshold metric	Gastrointestinal toxicity	Post-report random forest	top10%	threshold=0.476; sensitivity=23.7%; specificity=94.2%; PPV=56.0%; NPV=80.0%; F1=0.333; enrichment=2.37	NA	top-k threshold for FAERS signal-triage workload planning
Threshold metric	Gastrointestinal toxicity	Post-report random forest	top20%	threshold=0.389; sensitivity=40.7%; specificity=86.4%; PPV=48.1%; NPV=82.5%; F1=0.441; enrichment=2.04	NA	top-k threshold for FAERS signal-triage workload planning
Threshold metric	Hematologic toxicity	Post-report elastic-net logistic	top5%	threshold=0.751; sensitivity=10.2%; specificity=96.6%; PPV=47.7%; NPV=78.0%; F1=0.168; enrichment=2.04	NA	top-k threshold for FAERS signal-triage workload planning
Threshold metric	Hematologic toxicity	Post-report elastic-net logistic	top10%	threshold=0.712; sensitivity=18.9%; specificity=92.7%; PPV=44.1%; NPV=79.0%; F1=0.265; enrichment=1.89	NA	top-k threshold for FAERS signal-triage workload planning
Threshold metric	Hematologic toxicity	Post-report elastic-net logistic	top20%	threshold=0.664; sensitivity=35.8%; specificity=84.8%; PPV=41.7%; NPV=81.3%; F1=0.385; enrichment=1.79	NA	top-k threshold for FAERS signal-triage workload planning
Threshold metric	Hematologic toxicity	Baseline-only elastic-net logistic	top5%	threshold=0.735; sensitivity=9.5%; specificity=96.4%; PPV=44.3%; NPV=77.8%; F1=0.157; enrichment=1.9	NA	top-k threshold for FAERS signal-triage workload planning
Threshold metric	Hematologic toxicity	Baseline-only elastic-net logistic	top10%	threshold=0.705; sensitivity=18.0%; specificity=92.4%; PPV=42.0%; NPV=78.7%; F1=0.252; enrichment=1.8	NA	top-k threshold for FAERS signal-triage workload planning
Threshold metric	Hematologic toxicity	Baseline-only elastic-net logistic	top20%	threshold=0.661; sensitivity=34.5%; specificity=84.4%; PPV=40.2%; NPV=80.9%; F1=0.371; enrichment=1.72	NA	top-k threshold for FAERS signal-triage workload planning
Threshold metric	Hematologic toxicity	Post-report random forest	top5%	threshold=0.469; sensitivity=9.8%; specificity=96.4%; PPV=45.5%; NPV=77.8%;	NA	top-k threshold for FAERS signal-triage workload planning

Evidence layer	AESI	Model	Item	Metric values	Feature contribution	Use or boundary
				F1=0.161; enrichment=1.95		
Threshold metric	Hematologic toxicity	Post-report random forest	top10%	threshold=0.427; sensitivity=19.9%; specificity=93.0%; PPV=46.5%; NPV=79.2%; F1=0.279; enrichment=1.99	NA	top-k threshold for FAERS signal-triage workload planning
Threshold metric	Hematologic toxicity	Post-report random forest	top20%	threshold=0.377; sensitivity=37.0%; specificity=85.2%; PPV=43.2%; NPV=81.6%; F1=0.399; enrichment=1.85	NA	top-k threshold for FAERS signal-triage workload planning
Threshold metric	Hepatobiliary toxicity	Post-report elastic-net logistic	top5%	threshold=0.824; sensitivity=17.5%; specificity=96.0%; PPV=26.5%; NPV=93.4%; F1=0.211; enrichment=3.5	NA	top-k threshold for FAERS signal-triage workload planning
Threshold metric	Hepatobiliary toxicity	Post-report elastic-net logistic	top10%	threshold=0.784; sensitivity=31.9%; specificity=91.8%; PPV=24.1%; NPV=94.3%; F1=0.275; enrichment=3.19	NA	top-k threshold for FAERS signal-triage workload planning
Threshold metric	Hepatobiliary toxicity	Post-report elastic-net logistic	top20%	threshold=0.681; sensitivity=53.5%; specificity=82.7%; PPV=20.2%; NPV=95.6%; F1=0.294; enrichment=2.68	NA	top-k threshold for FAERS signal-triage workload planning
Threshold metric	Hepatobiliary toxicity	Baseline-only elastic-net logistic	top5%	threshold=0.805; sensitivity=17.2%; specificity=96.0%; PPV=26.0%; NPV=93.4%; F1=0.207; enrichment=3.44	NA	top-k threshold for FAERS signal-triage workload planning
Threshold metric	Hepatobiliary toxicity	Baseline-only elastic-net logistic	top10%	threshold=0.779; sensitivity=31.5%; specificity=91.8%; PPV=23.8%; NPV=94.3%; F1=0.272; enrichment=3.15	NA	top-k threshold for FAERS signal-triage workload planning
Threshold metric	Hepatobiliary toxicity	Baseline-only elastic-net logistic	top20%	threshold=0.686; sensitivity=53.5%; specificity=82.7%; PPV=20.2%; NPV=95.6%; F1=0.293; enrichment=2.67	NA	top-k threshold for FAERS signal-triage workload planning
Threshold metric	Hepatobiliary toxicity	Post-report random forest	top5%	threshold=0.571; sensitivity=18.1%; specificity=96.1%; PPV=27.4%; NPV=93.5%; F1=0.218; enrichment=3.63	NA	top-k threshold for FAERS signal-triage workload planning
Threshold metric	Hepatobiliary toxicity	Post-report random forest	top10%	threshold=0.487; sensitivity=33.0%; specificity=91.9%; PPV=24.9%; NPV=94.4%;	NA	top-k threshold for FAERS signal-triage workload planning

Evidence layer	AESI	Model	Item	Metric values	Feature contribution	Use or boundary
				F1=0.284; enrichment=3.3		
Threshold metric	Hepatobiliary toxicity	Post-report random forest	top20%	threshold=0.358; sensitivity=54.5%; specificity=82.8%; PPV=20.6%; NPV=95.7%; F1=0.299; enrichment=2.72	NA	top-k threshold for FAERS signal-triage workload planning
Threshold metric	ILD/pneumonitis	Post-report elastic-net logistic	top5%	threshold=0.755; sensitivity=22.3%; specificity=95.2%; PPV=6.4%; NPV=98.8%; F1=0.099; enrichment=4.46	NA	top-k threshold for FAERS signal-triage workload planning
Threshold metric	ILD/pneumonitis	Post-report elastic-net logistic	top10%	threshold=0.69; sensitivity=33.8%; specificity=90.3%; PPV=4.8%; NPV=98.9%; F1=0.084; enrichment=3.38	NA	top-k threshold for FAERS signal-triage workload planning
Threshold metric	ILD/pneumonitis	Post-report elastic-net logistic	top20%	threshold=0.565; sensitivity=49.5%; specificity=80.4%; PPV=3.5%; NPV=99.1%; F1=0.066; enrichment=2.47	NA	top-k threshold for FAERS signal-triage workload planning
Threshold metric	ILD/pneumonitis	Baseline-only elastic-net logistic	top5%	threshold=0.728; sensitivity=23.6%; specificity=95.3%; PPV=6.7%; NPV=98.9%; F1=0.105; enrichment=4.71	NA	top-k threshold for FAERS signal-triage workload planning
Threshold metric	ILD/pneumonitis	Baseline-only elastic-net logistic	top10%	threshold=0.655; sensitivity=33.9%; specificity=90.3%; PPV=4.8%; NPV=99.0%; F1=0.085; enrichment=3.39	NA	top-k threshold for FAERS signal-triage workload planning
Threshold metric	ILD/pneumonitis	Baseline-only elastic-net logistic	top20%	threshold=0.56; sensitivity=50.2%; specificity=80.4%; PPV=3.6%; NPV=99.1%; F1=0.067; enrichment=2.51	NA	top-k threshold for FAERS signal-triage workload planning
Threshold metric	ILD/pneumonitis	Post-report random forest	top5%	threshold=0.482; sensitivity=26.4%; specificity=95.3%; PPV=7.6%; NPV=98.9%; F1=0.117; enrichment=5.28	NA	top-k threshold for FAERS signal-triage workload planning
Threshold metric	ILD/pneumonitis	Post-report random forest	top10%	threshold=0.414; sensitivity=36.8%; specificity=90.4%; PPV=5.3%; NPV=99.0%; F1=0.092; enrichment=3.68	NA	top-k threshold for FAERS signal-triage workload planning
Threshold metric	ILD/pneumonitis	Post-report random forest	top20%	threshold=0.338; sensitivity=51.4%; specificity=80.5%; PPV=3.7%; NPV=99.1%;	NA	top-k threshold for FAERS signal-triage workload planning

Evidence layer	AESI	Model	Item	Metric values	Feature contribution	Use or boundary
				F1=0.069; enrichment=2.57		
Threshold metric	Infection/febrile events	Post-report elastic-net logistic	top5%	threshold=0.648; sensitivity=10.7%; specificity=95.4%; PPV=14.2%; NPV=93.8%; F1=0.122; enrichment=2.14	NA	top-k threshold for FAERS signal-triage workload planning
Threshold metric	Infection/febrile events	Post-report elastic-net logistic	top10%	threshold=0.624; sensitivity=23.4%; specificity=90.9%; PPV=15.5%; NPV=94.4%; F1=0.186; enrichment=2.34	NA	top-k threshold for FAERS signal-triage workload planning
Threshold metric	Infection/febrile events	Post-report elastic-net logistic	top20%	threshold=0.581; sensitivity=41.3%; specificity=81.5%; PPV=13.7%; NPV=95.1%; F1=0.205; enrichment=2.06	NA	top-k threshold for FAERS signal-triage workload planning
Threshold metric	Infection/febrile events	Baseline-only elastic-net logistic	top5%	threshold=0.643; sensitivity=10.7%; specificity=95.4%; PPV=14.2%; NPV=93.8%; F1=0.122; enrichment=2.14	NA	top-k threshold for FAERS signal-triage workload planning
Threshold metric	Infection/febrile events	Baseline-only elastic-net logistic	top10%	threshold=0.62; sensitivity=24.0%; specificity=91.0%; PPV=15.9%; NPV=94.4%; F1=0.191; enrichment=2.4	NA	top-k threshold for FAERS signal-triage workload planning
Threshold metric	Infection/febrile events	Baseline-only elastic-net logistic	top20%	threshold=0.579; sensitivity=41.2%; specificity=81.5%; PPV=13.7%; NPV=95.1%; F1=0.205; enrichment=2.06	NA	top-k threshold for FAERS signal-triage workload planning
Threshold metric	Infection/febrile events	Post-report random forest	top5%	threshold=0.383; sensitivity=14.6%; specificity=95.7%; PPV=19.3%; NPV=94.0%; F1=0.166; enrichment=2.92	NA	top-k threshold for FAERS signal-triage workload planning
Threshold metric	Infection/febrile events	Post-report random forest	top10%	threshold=0.335; sensitivity=25.1%; specificity=91.1%; PPV=16.6%; NPV=94.5%; F1=0.2; enrichment=2.51	NA	top-k threshold for FAERS signal-triage workload planning
Threshold metric	Infection/febrile events	Post-report random forest	top20%	threshold=0.272; sensitivity=41.1%; specificity=81.5%; PPV=13.6%; NPV=95.1%; F1=0.204; enrichment=2.05	NA	top-k threshold for FAERS signal-triage workload planning
Threshold metric	QT/cardiac toxicity	Post-report elastic-net logistic	top5%	threshold=0.806; sensitivity=27.9%; specificity=95.4%; PPV=9.2%; NPV=98.7%;	NA	top-k threshold for FAERS signal-triage workload planning

Evidence layer	AESI	Model	Item	Metric values	Feature contribution	Use or boundary
				F1=0.139; enrichment=5.58		
Threshold metric	QT/cardiac toxicity	Post-report elastic-net logistic	top10%	threshold=0.793; sensitivity=45.1%; specificity=90.6%; PPV=7.5%; NPV=99.0%; F1=0.128; enrichment=4.51	NA	top-k threshold for FAERS signal-triage workload planning
Threshold metric	QT/cardiac toxicity	Post-report elastic-net logistic	top20%	threshold=0.688; sensitivity=68.2%; specificity=80.8%; PPV=5.6%; NPV=99.3%; F1=0.104; enrichment=3.41	NA	top-k threshold for FAERS signal-triage workload planning
Threshold metric	QT/cardiac toxicity	Baseline-only elastic-net logistic	top5%	threshold=0.824; sensitivity=26.1%; specificity=95.4%; PPV=8.6%; NPV=98.7%; F1=0.13; enrichment=5.21	NA	top-k threshold for FAERS signal-triage workload planning
Threshold metric	QT/cardiac toxicity	Baseline-only elastic-net logistic	top10%	threshold=0.802; sensitivity=44.0%; specificity=90.6%; PPV=7.3%; NPV=99.0%; F1=0.125; enrichment=4.4	NA	top-k threshold for FAERS signal-triage workload planning
Threshold metric	QT/cardiac toxicity	Baseline-only elastic-net logistic	top20%	threshold=0.691; sensitivity=68.5%; specificity=80.8%; PPV=5.7%; NPV=99.3%; F1=0.105; enrichment=3.43	NA	top-k threshold for FAERS signal-triage workload planning
Threshold metric	QT/cardiac toxicity	Post-report random forest	top5%	threshold=0.7; sensitivity=19.0%; specificity=95.2%; PPV=6.3%; NPV=98.6%; F1=0.094; enrichment=3.8	NA	top-k threshold for FAERS signal-triage workload planning
Threshold metric	QT/cardiac toxicity	Post-report random forest	top10%	threshold=0.637; sensitivity=37.2%; specificity=90.5%; PPV=6.1%; NPV=98.8%; F1=0.106; enrichment=3.72	NA	top-k threshold for FAERS signal-triage workload planning
Threshold metric	QT/cardiac toxicity	Post-report random forest	top20%	threshold=0.515; sensitivity=66.2%; specificity=80.8%; PPV=5.5%; NPV=99.3%; F1=0.101; enrichment=3.31	NA	top-k threshold for FAERS signal-triage workload planning
Threshold metric	Renal/creatinine abnormality	Post-report elastic-net logistic	top5%	threshold=0.784; sensitivity=14.8%; specificity=95.5%; PPV=13.7%; NPV=95.8%; F1=0.142; enrichment=2.96	NA	top-k threshold for FAERS signal-triage workload planning
Threshold metric	Renal/creatinine abnormality	Post-report elastic-net logistic	top10%	threshold=0.717; sensitivity=26.0%; specificity=90.8%; PPV=12.1%; NPV=96.2%;	NA	top-k threshold for FAERS signal-triage workload planning

Evidence layer	AESI	Model	Item	Metric values	Feature contribution	Use or boundary
				F1=0.165; enrichment=2.6		
Threshold metric	Renal/creatinine abnormality	Post-report elastic-net logistic	top20%	threshold=0.628; sensitivity=43.9%; specificity=81.2%; PPV=10.2%; NPV=96.8%; F1=0.165; enrichment=2.2	NA	top-k threshold for FAERS signal-triage workload planning
Threshold metric	Renal/creatinine abnormality	Baseline-only elastic-net logistic	top5%	threshold=0.793; sensitivity=14.2%; specificity=95.4%; PPV=13.2%; NPV=95.8%; F1=0.137; enrichment=2.84	NA	top-k threshold for FAERS signal-triage workload planning
Threshold metric	Renal/creatinine abnormality	Baseline-only elastic-net logistic	top10%	threshold=0.724; sensitivity=25.6%; specificity=90.8%; PPV=11.8%; NPV=96.2%; F1=0.162; enrichment=2.56	NA	top-k threshold for FAERS signal-triage workload planning
Threshold metric	Renal/creatinine abnormality	Baseline-only elastic-net logistic	top20%	threshold=0.632; sensitivity=45.6%; specificity=81.2%; PPV=10.6%; NPV=96.9%; F1=0.172; enrichment=2.28	NA	top-k threshold for FAERS signal-triage workload planning
Threshold metric	Renal/creatinine abnormality	Post-report random forest	top5%	threshold=0.493; sensitivity=17.6%; specificity=95.6%; PPV=16.3%; NPV=96.0%; F1=0.169; enrichment=3.52	NA	top-k threshold for FAERS signal-triage workload planning
Threshold metric	Renal/creatinine abnormality	Post-report random forest	top10%	threshold=0.422; sensitivity=30.5%; specificity=91.0%; PPV=14.1%; NPV=96.4%; F1=0.193; enrichment=3.05	NA	top-k threshold for FAERS signal-triage workload planning
Threshold metric	Renal/creatinine abnormality	Post-report random forest	top20%	threshold=0.319; sensitivity=47.7%; specificity=81.3%; PPV=11.1%; NPV=97.0%; F1=0.18; enrichment=2.39	NA	top-k threshold for FAERS signal-triage workload planning
Threshold metric	VTE/thrombotic events	Post-report elastic-net logistic	top5%	threshold=0.713; sensitivity=13.1%; specificity=95.2%; PPV=6.3%; NPV=97.8%; F1=0.085; enrichment=2.62	NA	top-k threshold for FAERS signal-triage workload planning
Threshold metric	VTE/thrombotic events	Post-report elastic-net logistic	top10%	threshold=0.667; sensitivity=26.4%; specificity=90.4%; PPV=6.3%; NPV=98.0%; F1=0.102; enrichment=2.64	NA	top-k threshold for FAERS signal-triage workload planning
Threshold metric	VTE/thrombotic events	Post-report elastic-net logistic	top20%	threshold=0.589; sensitivity=43.5%; specificity=80.6%; PPV=5.2%; NPV=98.3%;	NA	top-k threshold for FAERS signal-triage workload planning

Evidence layer	AESI	Model	Item	Metric values	Feature contribution	Use or boundary
				F1=0.093; enrichment=2.18		
Threshold metric	VTE/thrombotic events	Baseline-only elastic-net logistic	top5%	threshold=0.719; sensitivity=12.7%; specificity=95.2%; PPV=6.1%; NPV=97.8%; F1=0.082; enrichment=2.54	NA	top-k threshold for FAERS signal-triage workload planning
Threshold metric	VTE/thrombotic events	Baseline-only elastic-net logistic	top10%	threshold=0.67; sensitivity=25.5%; specificity=90.4%; PPV=6.1%; NPV=98.0%; F1=0.098; enrichment=2.55	NA	top-k threshold for FAERS signal-triage workload planning
Threshold metric	VTE/thrombotic events	Baseline-only elastic-net logistic	top20%	threshold=0.583; sensitivity=44.0%; specificity=80.6%; PPV=5.3%; NPV=98.3%; F1=0.094; enrichment=2.2	NA	top-k threshold for FAERS signal-triage workload planning
Threshold metric	VTE/thrombotic events	Post-report random forest	top5%	threshold=0.429; sensitivity=15.6%; specificity=95.3%; PPV=7.4%; NPV=97.9%; F1=0.101; enrichment=3.12	NA	top-k threshold for FAERS signal-triage workload planning
Threshold metric	VTE/thrombotic events	Post-report random forest	top10%	threshold=0.368; sensitivity=26.5%; specificity=90.4%; PPV=6.3%; NPV=98.0%; F1=0.102; enrichment=2.65	NA	top-k threshold for FAERS signal-triage workload planning
Threshold metric	VTE/thrombotic events	Post-report random forest	top20%	threshold=0.29; sensitivity=43.1%; specificity=80.6%; PPV=5.2%; NPV=98.3%; F1=0.092; enrichment=2.16	NA	top-k threshold for FAERS signal-triage workload planning
Grouped feature contribution	Gastrointestinal toxicity	Post-report elastic-net logistic	Grouped importance	NA	reporter country/region; terms=3; scaled importance=0.197	Leading feature contribution in the primary post-report model.
Grouped feature contribution	Gastrointestinal toxicity	Post-report elastic-net logistic	Grouped importance	NA	concomitant medication group; terms=3; scaled importance=0.192	Leading feature contribution in the primary post-report model.
Grouped feature contribution	Gastrointestinal toxicity	Post-report elastic-net logistic	Grouped importance	NA	reporter type; terms=4; scaled importance=0.17	Leading feature contribution in the primary post-report model.
Grouped feature contribution	Gastrointestinal toxicity	Post-report elastic-net logistic	Grouped importance	NA	reported TTO group; terms=3; scaled importance=0.126	Leading feature contribution in the primary post-report model.
Grouped feature contribution	Gastrointestinal toxicity	Post-report elastic-net logistic	Grouped importance	NA	drug; terms=2; scaled importance=0.124	Leading feature contribution in the primary post-report model.
Grouped feature contribution	Hematologic toxicity	Post-report elastic-net logistic	Grouped importance	NA	concomitant medication group; terms=3; scaled importance=0.239	Leading feature contribution in the primary post-report model.

Evidence layer	AESI	Model	Item	Metric values	Feature contribution	Use or boundary
Grouped feature contribution	Hematologic toxicity	Post-report elastic-net logistic	Grouped importance	NA	reporter country/region; terms=6; scaled importance=0.159	Leading feature contribution in the primary post-report model.
Grouped feature contribution	Hematologic toxicity	Post-report elastic-net logistic	Grouped importance	NA	reported TTO group; terms=3; scaled importance=0.154	Leading feature contribution in the primary post-report model.
Grouped feature contribution	Hematologic toxicity	Post-report elastic-net logistic	Grouped importance	NA	report year; terms=7; scaled importance=0.141	Leading feature contribution in the primary post-report model.
Grouped feature contribution	Hematologic toxicity	Post-report elastic-net logistic	Grouped importance	NA	reporter type; terms=5; scaled importance=0.103	Leading feature contribution in the primary post-report model.
Grouped feature contribution	Hepatobiliary toxicity	Post-report elastic-net logistic	Grouped importance	NA	reporter country/region; terms=3; scaled importance=0.362	Leading feature contribution in the primary post-report model.
Grouped feature contribution	Hepatobiliary toxicity	Post-report elastic-net logistic	Grouped importance	NA	drug; terms=2; scaled importance=0.197	Leading feature contribution in the primary post-report model.
Grouped feature contribution	Hepatobiliary toxicity	Post-report elastic-net logistic	Grouped importance	NA	reporter type; terms=3; scaled importance=0.102	Leading feature contribution in the primary post-report model.
Grouped feature contribution	Hepatobiliary toxicity	Post-report elastic-net logistic	Grouped importance	NA	age group; terms=2; scaled importance=0.094	Leading feature contribution in the primary post-report model.
Grouped feature contribution	Hepatobiliary toxicity	Post-report elastic-net logistic	Grouped importance	NA	concomitant medication group; terms=3; scaled importance=0.073	Leading feature contribution in the primary post-report model.
Grouped feature contribution	ILD/pneumonitis	Post-report elastic-net logistic	Grouped importance	NA	reporter country/region; terms=3; scaled importance=0.349	Leading feature contribution in the primary post-report model.
Grouped feature contribution	ILD/pneumonitis	Post-report elastic-net logistic	Grouped importance	NA	reporter type; terms=2; scaled importance=0.224	Leading feature contribution in the primary post-report model.
Grouped feature contribution	ILD/pneumonitis	Post-report elastic-net logistic	Grouped importance	NA	drug; terms=2; scaled importance=0.179	Leading feature contribution in the primary post-report model.
Grouped feature contribution	ILD/pneumonitis	Post-report elastic-net logistic	Grouped importance	NA	concomitant endocrine therapy; terms=1; scaled importance=0.078	Leading feature contribution in the primary post-report model.
Grouped feature contribution	ILD/pneumonitis	Post-report elastic-net logistic	Grouped importance	NA	age group; terms=2; scaled importance=0.058	Leading feature contribution in the primary post-report model.
Grouped feature contribution	Infection/febrile events	Post-report elastic-net logistic	Grouped importance	NA	concomitant medication group; terms=3; scaled importance=0.484	Leading feature contribution in the primary post-report model.
Grouped feature contribution	Infection/febrile events	Post-report elastic-net logistic	Grouped importance	NA	reporter country/region; terms=1; scaled importance=0.084	Leading feature contribution in the primary post-report model.

Evidence layer	AESI	Model	Item	Metric values	Feature contribution	Use or boundary
Grouped feature contribution	Infection/febrile events	Post-report elastic-net logistic	Grouped importance	NA	sex; terms=1; scaled importance=0.084	Leading feature contribution in the primary post-report model.
Grouped feature contribution	Infection/febrile events	Post-report elastic-net logistic	Grouped importance	NA	drug; terms=2; scaled importance=0.082	Leading feature contribution in the primary post-report model.
Grouped feature contribution	Infection/febrile events	Post-report elastic-net logistic	Grouped importance	NA	age group; terms=2; scaled importance=0.078	Leading feature contribution in the primary post-report model.
Grouped feature contribution	QT/cardiac toxicity	Post-report elastic-net logistic	Grouped importance	NA	drug; terms=2; scaled importance=0.572	Leading feature contribution in the primary post-report model.
Grouped feature contribution	QT/cardiac toxicity	Post-report elastic-net logistic	Grouped importance	NA	reporter country/region; terms=2; scaled importance=0.303	Leading feature contribution in the primary post-report model.
Grouped feature contribution	QT/cardiac toxicity	Post-report elastic-net logistic	Grouped importance	NA	reporter type; terms=2; scaled importance=0.052	Leading feature contribution in the primary post-report model.
Grouped feature contribution	QT/cardiac toxicity	Post-report elastic-net logistic	Grouped importance	NA	reported TTO group; terms=1; scaled importance=0.027	Leading feature contribution in the primary post-report model.
Grouped feature contribution	QT/cardiac toxicity	Post-report elastic-net logistic	Grouped importance	NA	concomitant endocrine therapy; terms=1; scaled importance=0.018	Leading feature contribution in the primary post-report model.
Grouped feature contribution	Renal/creatinine abnormality	Post-report elastic-net logistic	Grouped importance	NA	reporter country/region; terms=3; scaled importance=0.244	Leading feature contribution in the primary post-report model.
Grouped feature contribution	Renal/creatinine abnormality	Post-report elastic-net logistic	Grouped importance	NA	concomitant medication group; terms=3; scaled importance=0.208	Leading feature contribution in the primary post-report model.
Grouped feature contribution	Renal/creatinine abnormality	Post-report elastic-net logistic	Grouped importance	NA	drug; terms=2; scaled importance=0.134	Leading feature contribution in the primary post-report model.
Grouped feature contribution	Renal/creatinine abnormality	Post-report elastic-net logistic	Grouped importance	NA	reporter type; terms=4; scaled importance=0.126	Leading feature contribution in the primary post-report model.
Grouped feature contribution	Renal/creatinine abnormality	Post-report elastic-net logistic	Grouped importance	NA	age group; terms=3; scaled importance=0.118	Leading feature contribution in the primary post-report model.
Grouped feature contribution	VTE/thrombotic events	Post-report elastic-net logistic	Grouped importance	NA	reporter country/region; terms=4; scaled importance=0.365	Leading feature contribution in the primary post-report model.
Grouped feature contribution	VTE/thrombotic events	Post-report elastic-net logistic	Grouped importance	NA	concomitant medication group; terms=3; scaled importance=0.175	Leading feature contribution in the primary post-report model.
Grouped feature contribution	VTE/thrombotic events	Post-report elastic-net logistic	Grouped importance	NA	reporter type; terms=3; scaled importance=0.118	Leading feature contribution in the primary post-report model.

Evidence layer	AESI	Model	Item	Metric values	Feature contribution	Use or boundary
Grouped feature contribution	VTE/thrombotic events	Post-report elastic-net logistic	Grouped importance	NA	drug; terms=2; scaled importance=0.115	Leading feature contribution in the primary post-report model.
Grouped feature contribution	VTE/thrombotic events	Post-report elastic-net logistic	Grouped importance	NA	reported TTO group; terms=3; scaled importance=0.096	Leading feature contribution in the primary post-report model.
Practical interpretation	NA	NA	Elevated ML priority score	NA	NA	Use as a report-level triage flag to prioritize pharmacovigilance review of a drug-AESI domain. Clinical plausibility review and source-level confirmation complete this triage signal.
Practical interpretation	NA	NA	Top-k threshold metrics	NA	NA	Use to estimate review burden and enrichment when selecting reports/domains for manual safety review. Top 5%, 10%, and 20% cutoffs are operational review strata for report-level triage.
Practical interpretation	NA	NA	Discordance between ML and ROR	NA	NA	Trigger review of reporting context, reporter region/type, concomitant medication burden, and TTO coding. Discordance identifies manual-review priorities across complementary methods.
Practical interpretation	NA	NA	Rare AESI with lower AUROC	NA	NA	Interpret as limited separability within FAERS report metadata, especially when baseline risk factors are Missing. Clinical review remains important for lower-discrimination AESI.
Practical interpretation	NA	NA	Clinical confirmation	NA	NA	Chart review, denominator-based cohorts, and prospective studies provide denominator-based risk estimation and practice-level evidence. FAERS ML estimates report-level prioritization; denominator-based designs address incidence, causality, and individual risk.

Table S8. Triangulated signal credibility and FAERS reporting context stratifications.

Triangulated signal summaries and FAERS context stratifications were converted into a single long table with one shared header.

Evidence layer	Stratification variable	Stratum	Drug	AESI	Reports	AESI-positive reports	Within-stratum %	Signal summary	Interpretation boundary
Triangulated signal credibility	Signal matrix	Prespecified label/sentinel AESI	NA	Hematologic toxicity	NA	NA	NA	Top by disproportionality: Palbociclib; top by intraclass OR: Palbociclib; top by ML priority: Palbociclib; institutional observed: Yes; institutional consequential: Yes	median reported TTO range 28-50 days; availability 22.3%; Tier 1 high-priority triangulated reporting signal; Tiering summarizes reporting-signal credibility for pharmacovigilance prioritization.
Triangulated signal credibility	Signal matrix	Prespecified label/sentinel AESI	NA	Gastrointestinal toxicity	NA	NA	NA	Top by disproportionality: Abemaciclib; top by intraclass OR: Abemaciclib; top by ML priority: Abemaciclib; institutional observed: Yes; institutional consequential: Yes	median reported TTO range 25-54 days; availability 17.2%; Tier 1 high-priority triangulated reporting signal; Tiering summarizes reporting-signal credibility for pharmacovigilance prioritization.
Triangulated signal credibility	Signal matrix	Prespecified label/sentinel AESI	NA	Hepatobiliary toxicity	NA	NA	NA	Top by disproportionality: Ribociclib; top by intraclass OR: Ribociclib; top by ML priority: Ribociclib; institutional observed: Yes; institutional consequential: Yes	median reported TTO range 41-70 days; availability 29.5%; Tier 1 high-priority triangulated reporting signal; Tiering summarizes reporting-signal credibility for pharmacovigilance prioritization.
Triangulated signal credibility	Signal matrix	Prespecified label/sentinel AESI	NA	QT/cardiac toxicity	NA	NA	NA	Top by disproportionality: Ribociclib; top by intraclass OR: Ribociclib; top by ML priority: Ribociclib; institutional observed: No; institutional consequential: No	median reported TTO range 24-76 days; availability 29.9%; Tier 2 moderate-priority reporting signal; Tiering summarizes reporting-signal credibility for pharmacovigilance prioritization.
Triangulated signal credibility	Signal matrix	Prespecified label/sentinel AESI	NA	VTE/thrombotic events	NA	NA	NA	Top by disproportionality: Abemaciclib; top by intraclass OR: Abemaciclib; top by ML priority: Abemaciclib; institutional observed: No; institutional consequential: No	median reported TTO range 76-103 days; availability 28.6%; Tier 2 moderate-priority reporting signal; Tiering summarizes reporting-signal credibility for pharmacovigilance prioritization.
Triangulated signal credibility	Signal matrix	Prespecified label/sentinel AESI	NA	ILD/pneumonitis	NA	NA	NA	Top by disproportionality: Abemaciclib; top by intraclass OR: Abemaciclib; top by ML priority: Abemaciclib; institutional observed: No; institutional	median reported TTO range 70-138 days; availability 31.7%; Tier 2 moderate-priority reporting signal; Tiering summarizes reporting-signal credibility for pharmacovigilance

Evidence layer	Stratification variable	Stratum	Drug	AESI	Reports	AESI-positive reports	Within-stratum %	Signal summary	Interpretation boundary
								consequential: No	prioritization.
Triangulated signal credibility	Signal matrix	Prespecified label/sentinel AESI	NA	Infection/febrile events	NA	NA	NA	Top by disproportionality: Abemaciclib; top by intraclass OR: Ribociclib; top by ML priority: Ribociclib; institutional observed: Yes; institutional consequential: Yes	median reported TTO range 38-102 days; availability 25.9%; Tier 1 high-priority triangulated reporting signal; Tiering summarizes reporting-signal credibility for pharmacovigilance prioritization.
Triangulated signal credibility	Signal matrix	Prespecified label/sentinel AESI	NA	Renal/creatinine abnormality	NA	NA	NA	Top by disproportionality: Ribociclib; top by intraclass OR: Abemaciclib; top by ML priority: Abemaciclib; institutional observed: Yes; institutional consequential: Yes	median reported TTO range 17-70 days; availability 29.0%; Tier 1 high-priority triangulated reporting signal; Tiering summarizes reporting-signal credibility for pharmacovigilance prioritization.
FAERS reporting context stratification	FAERS endocrine therapy recording	Concomitant endocrine therapy recorded	Abemaciclib	Hematologic toxicity	3,283	724	22.1%	NA	Exploratory concomitant endocrine reporting context.
FAERS reporting context stratification	FAERS endocrine therapy recording	Concomitant endocrine therapy recorded	Palbociclib	Hematologic toxicity	25,576	8,203	32.1%	NA	Exploratory concomitant endocrine reporting context.
FAERS reporting context stratification	FAERS endocrine therapy recording	Concomitant endocrine therapy recorded	Ribociclib	Hematologic toxicity	6,751	2,036	30.2%	NA	Exploratory concomitant endocrine reporting context.
FAERS reporting context stratification	FAERS endocrine therapy recording	No endocrine therapy recorded	Abemaciclib	Hematologic toxicity	12,047	1,388	11.5%	NA	Exploratory concomitant endocrine reporting context.
FAERS reporting context stratification	FAERS endocrine therapy recording	No endocrine therapy recorded	Palbociclib	Hematologic toxicity	55,106	11,482	20.8%	NA	Exploratory concomitant endocrine reporting context.
FAERS reporting context stratification	FAERS endocrine therapy recording	No endocrine therapy recorded	Ribociclib	Hematologic toxicity	13,008	2,145	16.5%	NA	Exploratory concomitant endocrine reporting context.
FAERS reporting context stratification	FAERS endocrine therapy recording	Concomitant endocrine therapy recorded	Abemaciclib	Gastrointestinal toxicity	3,283	1,134	34.5%	NA	Exploratory concomitant endocrine reporting context.
FAERS reporting context stratification	FAERS endocrine therapy recording	Concomitant endocrine therapy recorded	Palbociclib	Gastrointestinal toxicity	25,576	5,516	21.6%	NA	Exploratory concomitant endocrine reporting context.
FAERS reporting context stratification	FAERS endocrine therapy recording	Concomitant endocrine therapy recorded	Ribociclib	Gastrointestinal toxicity	6,751	1,589	23.5%	NA	Exploratory concomitant endocrine reporting context.
FAERS reporting context stratification	FAERS endocrine therapy recording	No endocrine therapy recorded	Abemaciclib	Gastrointestinal toxicity	12,047	4,646	38.6%	NA	Exploratory concomitant endocrine reporting context.
FAERS reporting context stratification	FAERS endocrine therapy recording	No endocrine therapy recorded	Palbociclib	Gastrointestinal toxicity	55,106	8,896	16.1%	NA	Exploratory concomitant endocrine reporting context.
FAERS reporting	FAERS endocrine	No endocrine therapy	Ribociclib	Gastrointestinal	13,008	2,335	18.0%	NA	Exploratory

Evidence layer	Stratification variable	Stratum	Drug	AESI	Reports	AESI-positive reports	Within-stratum %	Signal summary	Interpretation boundary
context stratification	therapy recording	recorded		toxicity					concomitant endocrine reporting context.
FAERS reporting context stratification	FAERS endocrine therapy recording	Concomitant endocrine therapy recorded	Abemaciclib	Hepatobiliary toxicity	3,283	500	15.2%	NA	Exploratory concomitant endocrine reporting context.
FAERS reporting context stratification	FAERS endocrine therapy recording	Concomitant endocrine therapy recorded	Palbociclib	Hepatobiliary toxicity	25,576	1,067	4.2%	NA	Exploratory concomitant endocrine reporting context.
FAERS reporting context stratification	FAERS endocrine therapy recording	Concomitant endocrine therapy recorded	Ribociclib	Hepatobiliary toxicity	6,751	1,247	18.5%	NA	Exploratory concomitant endocrine reporting context.
FAERS reporting context stratification	FAERS endocrine therapy recording	No endocrine therapy recorded	Abemaciclib	Hepatobiliary toxicity	12,047	623	5.2%	NA	Exploratory concomitant endocrine reporting context.
FAERS reporting context stratification	FAERS endocrine therapy recording	No endocrine therapy recorded	Palbociclib	Hepatobiliary toxicity	55,106	987	1.8%	NA	Exploratory concomitant endocrine reporting context.
FAERS reporting context stratification	FAERS endocrine therapy recording	No endocrine therapy recorded	Ribociclib	Hepatobiliary toxicity	13,008	1,431	11.0%	NA	Exploratory concomitant endocrine reporting context.
FAERS reporting context stratification	FAERS endocrine therapy recording	Concomitant endocrine therapy recorded	Abemaciclib	QT/cardiac toxicity	3,283	30	0.9%	NA	Exploratory concomitant endocrine reporting context.
FAERS reporting context stratification	FAERS endocrine therapy recording	Concomitant endocrine therapy recorded	Palbociclib	QT/cardiac toxicity	25,576	156	0.6%	NA	Exploratory concomitant endocrine reporting context.
FAERS reporting context stratification	FAERS endocrine therapy recording	Concomitant endocrine therapy recorded	Ribociclib	QT/cardiac toxicity	6,751	346	5.1%	NA	Exploratory concomitant endocrine reporting context.
FAERS reporting context stratification	FAERS endocrine therapy recording	No endocrine therapy recorded	Abemaciclib	QT/cardiac toxicity	12,047	41	0.3%	NA	Exploratory concomitant endocrine reporting context.
FAERS reporting context stratification	FAERS endocrine therapy recording	No endocrine therapy recorded	Palbociclib	QT/cardiac toxicity	55,106	149	0.3%	NA	Exploratory concomitant endocrine reporting context.
FAERS reporting context stratification	FAERS endocrine therapy recording	No endocrine therapy recorded	Ribociclib	QT/cardiac toxicity	13,008	603	4.6%	NA	Exploratory concomitant endocrine reporting context.
FAERS reporting context stratification	FAERS endocrine therapy recording	Concomitant endocrine therapy recorded	Abemaciclib	VTE/thrombotic events	3,283	127	3.9%	NA	Exploratory concomitant endocrine reporting context.
FAERS reporting context stratification	FAERS endocrine therapy recording	Concomitant endocrine therapy recorded	Palbociclib	VTE/thrombotic events	25,576	715	2.8%	NA	Exploratory concomitant endocrine reporting context.
FAERS reporting context stratification	FAERS endocrine therapy recording	Concomitant endocrine therapy recorded	Ribociclib	VTE/thrombotic events	6,751	248	3.7%	NA	Exploratory concomitant endocrine reporting context.
FAERS reporting context stratification	FAERS endocrine therapy recording	No endocrine therapy recorded	Abemaciclib	VTE/thrombotic events	12,047	388	3.2%	NA	Exploratory concomitant endocrine reporting context.
FAERS reporting context stratification	FAERS endocrine therapy recording	No endocrine therapy recorded	Palbociclib	VTE/thrombotic events	55,106	717	1.3%	NA	Exploratory concomitant endocrine reporting context.

Evidence layer	Stratification variable	Stratum	Drug	AESI	Reports	AESI-positive reports	Within-stratum %	Signal summary	Interpretation boundary
FAERS reporting context stratification	FAERS endocrine therapy recording	No endocrine therapy recorded	Ribociclib	VTE/thrombotic events	13,008	226	1.7%	NA	Exploratory concomitant endocrine reporting context.
FAERS reporting context stratification	FAERS endocrine therapy recording	Concomitant endocrine therapy recorded	Abemaciclib	ILD/pneumonitis	3,283	232	7.1%	NA	Exploratory concomitant endocrine reporting context.
FAERS reporting context stratification	FAERS endocrine therapy recording	Concomitant endocrine therapy recorded	Palbociclib	ILD/pneumonitis	25,576	248	1.0%	NA	Exploratory concomitant endocrine reporting context.
FAERS reporting context stratification	FAERS endocrine therapy recording	Concomitant endocrine therapy recorded	Ribociclib	ILD/pneumonitis	6,751	124	1.8%	NA	Exploratory concomitant endocrine reporting context.
FAERS reporting context stratification	FAERS endocrine therapy recording	No endocrine therapy recorded	Abemaciclib	ILD/pneumonitis	12,047	222	1.8%	NA	Exploratory concomitant endocrine reporting context.
FAERS reporting context stratification	FAERS endocrine therapy recording	No endocrine therapy recorded	Palbociclib	ILD/pneumonitis	55,106	238	0.4%	NA	Exploratory concomitant endocrine reporting context.
FAERS reporting context stratification	FAERS endocrine therapy recording	No endocrine therapy recorded	Ribociclib	ILD/pneumonitis	13,008	165	1.3%	NA	Exploratory concomitant endocrine reporting context.
FAERS reporting context stratification	FAERS endocrine therapy recording	Concomitant endocrine therapy recorded	Abemaciclib	Infection/febrile events	3,283	212	6.5%	NA	Exploratory concomitant endocrine reporting context.
FAERS reporting context stratification	FAERS endocrine therapy recording	Concomitant endocrine therapy recorded	Palbociclib	Infection/febrile events	25,576	2,223	8.7%	NA	Exploratory concomitant endocrine reporting context.
FAERS reporting context stratification	FAERS endocrine therapy recording	Concomitant endocrine therapy recorded	Ribociclib	Infection/febrile events	6,751	767	11.4%	NA	Exploratory concomitant endocrine reporting context.
FAERS reporting context stratification	FAERS endocrine therapy recording	No endocrine therapy recorded	Abemaciclib	Infection/febrile events	12,047	424	3.5%	NA	Exploratory concomitant endocrine reporting context.
FAERS reporting context stratification	FAERS endocrine therapy recording	No endocrine therapy recorded	Palbociclib	Infection/febrile events	55,106	2,594	4.7%	NA	Exploratory concomitant endocrine reporting context.
FAERS reporting context stratification	FAERS endocrine therapy recording	No endocrine therapy recorded	Ribociclib	Infection/febrile events	13,008	758	5.8%	NA	Exploratory concomitant endocrine reporting context.
FAERS reporting context stratification	FAERS endocrine therapy recording	Concomitant endocrine therapy recorded	Abemaciclib	Renal/creatinine abnormality	3,283	290	8.8%	NA	Exploratory concomitant endocrine reporting context.
FAERS reporting context stratification	FAERS endocrine therapy recording	Concomitant endocrine therapy recorded	Palbociclib	Renal/creatinine abnormality	25,576	968	3.8%	NA	Exploratory concomitant endocrine reporting context.
FAERS reporting context stratification	FAERS endocrine therapy recording	Concomitant endocrine therapy recorded	Ribociclib	Renal/creatinine abnormality	6,751	542	8.0%	NA	Exploratory concomitant endocrine reporting context.
FAERS reporting context stratification	FAERS endocrine therapy recording	No endocrine therapy recorded	Abemaciclib	Renal/creatinine abnormality	12,047	578	4.8%	NA	Exploratory concomitant endocrine reporting context.
FAERS reporting context stratification	FAERS endocrine therapy recording	No endocrine therapy recorded	Palbociclib	Renal/creatinine abnormality	55,106	929	1.7%	NA	Exploratory concomitant endocrine

Evidence layer	Stratification variable	Stratum	Drug	AESI	Reports	AESI-positive reports	Within-stratum %	Signal summary	Interpretation boundary
									reporting context.
FAERS reporting context stratification	FAERS endocrine therapy recording	No endocrine therapy recorded	Ribociclib	Renal/creatinine abnormality	13,008	595	4.6%	NA	Exploratory concomitant endocrine reporting context.
FAERS reporting context stratification	FAERS concomitant-medication count group	0	Abemaciclib	Hematologic toxicity	9,309	865	9.3%	NA	Exploratory concomitant-medication burden context.
FAERS reporting context stratification	FAERS concomitant-medication count group	0	Palbociclib	Hematologic toxicity	39,467	6,201	15.7%	NA	Exploratory concomitant-medication burden context.
FAERS reporting context stratification	FAERS concomitant-medication count group	0	Ribociclib	Hematologic toxicity	9,589	1,042	10.9%	NA	Exploratory concomitant-medication burden context.
FAERS reporting context stratification	FAERS concomitant-medication count group	1-2	Abemaciclib	Hematologic toxicity	3,990	784	19.6%	NA	Exploratory concomitant-medication burden context.
FAERS reporting context stratification	FAERS concomitant-medication count group	1-2	Palbociclib	Hematologic toxicity	21,374	6,243	29.2%	NA	Exploratory concomitant-medication burden context.
FAERS reporting context stratification	FAERS concomitant-medication count group	1-2	Ribociclib	Hematologic toxicity	5,409	1,373	25.4%	NA	Exploratory concomitant-medication burden context.
FAERS reporting context stratification	FAERS concomitant-medication count group	3-5	Abemaciclib	Hematologic toxicity	1,003	268	26.7%	NA	Exploratory concomitant-medication burden context.
FAERS reporting context stratification	FAERS concomitant-medication count group	3-5	Palbociclib	Hematologic toxicity	8,448	3,317	39.3%	NA	Exploratory concomitant-medication burden context.
FAERS reporting context stratification	FAERS concomitant-medication count group	3-5	Ribociclib	Hematologic toxicity	2,482	867	34.9%	NA	Exploratory concomitant-medication burden context.
FAERS reporting context stratification	FAERS concomitant-medication count group	>5	Abemaciclib	Hematologic toxicity	1,028	195	19.0%	NA	Exploratory concomitant-medication burden context.
FAERS reporting context stratification	FAERS concomitant-medication count group	>5	Palbociclib	Hematologic toxicity	11,393	3,924	34.4%	NA	Exploratory concomitant-medication burden context.
FAERS reporting context stratification	FAERS concomitant-medication count group	>5	Ribociclib	Hematologic toxicity	2,279	899	39.4%	NA	Exploratory concomitant-medication burden context.
FAERS reporting context stratification	FAERS concomitant-medication count group	0	Abemaciclib	Gastrointestinal toxicity	9,309	3,210	34.5%	NA	Exploratory concomitant-medication burden

Evidence layer	Stratification variable	Stratum	Drug	AESI	Reports	AESI-positive reports	Within-stratum %	Signal summary	Interpretation boundary
									context.
FAERS reporting context stratification	FAERS concomitant-medication count group	0	Palbociclib	Gastrointestinal toxicity	39,467	4,996	12.7%	NA	Exploratory concomitant-medication burden context.
FAERS reporting context stratification	FAERS concomitant-medication count group	0	Ribociclib	Gastrointestinal toxicity	9,589	1,420	14.8%	NA	Exploratory concomitant-medication burden context.
FAERS reporting context stratification	FAERS concomitant-medication count group	1-2	Abemaciclib	Gastrointestinal toxicity	3,990	1,764	44.2%	NA	Exploratory concomitant-medication burden context.
FAERS reporting context stratification	FAERS concomitant-medication count group	1-2	Palbociclib	Gastrointestinal toxicity	21,374	4,250	19.9%	NA	Exploratory concomitant-medication burden context.
FAERS reporting context stratification	FAERS concomitant-medication count group	1-2	Ribociclib	Gastrointestinal toxicity	5,409	1,058	19.6%	NA	Exploratory concomitant-medication burden context.
FAERS reporting context stratification	FAERS concomitant-medication count group	3-5	Abemaciclib	Gastrointestinal toxicity	1,003	459	45.8%	NA	Exploratory concomitant-medication burden context.
FAERS reporting context stratification	FAERS concomitant-medication count group	3-5	Palbociclib	Gastrointestinal toxicity	8,448	2,128	25.2%	NA	Exploratory concomitant-medication burden context.
FAERS reporting context stratification	FAERS concomitant-medication count group	3-5	Ribociclib	Gastrointestinal toxicity	2,482	668	26.9%	NA	Exploratory concomitant-medication burden context.
FAERS reporting context stratification	FAERS concomitant-medication count group	>5	Abemaciclib	Gastrointestinal toxicity	1,028	347	33.8%	NA	Exploratory concomitant-medication burden context.
FAERS reporting context stratification	FAERS concomitant-medication count group	>5	Palbociclib	Gastrointestinal toxicity	11,393	3,038	26.7%	NA	Exploratory concomitant-medication burden context.
FAERS reporting context stratification	FAERS concomitant-medication count group	>5	Ribociclib	Gastrointestinal toxicity	2,279	778	34.1%	NA	Exploratory concomitant-medication burden context.
FAERS reporting context stratification	FAERS concomitant-medication count group	0	Abemaciclib	Hepatobiliary toxicity	9,309	459	4.9%	NA	Exploratory concomitant-medication burden context.
FAERS reporting context stratification	FAERS concomitant-medication count group	0	Palbociclib	Hepatobiliary toxicity	39,467	650	1.6%	NA	Exploratory concomitant-medication burden context.
FAERS reporting context stratification	FAERS concomitant-medication count group	0	Ribociclib	Hepatobiliary toxicity	9,589	901	9.4%	NA	Exploratory concomitant-

Evidence layer	Stratification variable	Stratum	Drug	AESI	Reports	AESI-positive reports	Within-stratum %	Signal summary	Interpretation boundary
									medication burden context.
FAERS reporting context stratification	FAERS concomitant-medication count group	1-2	Abemaciclib	Hepatobiliary toxicity	3,990	403	10.1%	NA	Exploratory concomitant-medication burden context.
FAERS reporting context stratification	FAERS concomitant-medication count group	1-2	Palbociclib	Hepatobiliary toxicity	21,374	671	3.1%	NA	Exploratory concomitant-medication burden context.
FAERS reporting context stratification	FAERS concomitant-medication count group	1-2	Ribociclib	Hepatobiliary toxicity	5,409	824	15.2%	NA	Exploratory concomitant-medication burden context.
FAERS reporting context stratification	FAERS concomitant-medication count group	3-5	Abemaciclib	Hepatobiliary toxicity	1,003	157	15.7%	NA	Exploratory concomitant-medication burden context.
FAERS reporting context stratification	FAERS concomitant-medication count group	3-5	Palbociclib	Hepatobiliary toxicity	8,448	335	4.0%	NA	Exploratory concomitant-medication burden context.
FAERS reporting context stratification	FAERS concomitant-medication count group	3-5	Ribociclib	Hepatobiliary toxicity	2,482	481	19.4%	NA	Exploratory concomitant-medication burden context.
FAERS reporting context stratification	FAERS concomitant-medication count group	>5	Abemaciclib	Hepatobiliary toxicity	1,028	104	10.1%	NA	Exploratory concomitant-medication burden context.
FAERS reporting context stratification	FAERS concomitant-medication count group	>5	Palbociclib	Hepatobiliary toxicity	11,393	398	3.5%	NA	Exploratory concomitant-medication burden context.
FAERS reporting context stratification	FAERS concomitant-medication count group	>5	Ribociclib	Hepatobiliary toxicity	2,279	472	20.7%	NA	Exploratory concomitant-medication burden context.
FAERS reporting context stratification	FAERS concomitant-medication count group	0	Abemaciclib	QT/cardiac toxicity	9,309	28	0.3%	NA	Exploratory concomitant-medication burden context.
FAERS reporting context stratification	FAERS concomitant-medication count group	0	Palbociclib	QT/cardiac toxicity	39,467	100	0.3%	NA	Exploratory concomitant-medication burden context.
FAERS reporting context stratification	FAERS concomitant-medication count group	0	Ribociclib	QT/cardiac toxicity	9,589	401	4.2%	NA	Exploratory concomitant-medication burden context.
FAERS reporting context stratification	FAERS concomitant-medication count group	1-2	Abemaciclib	QT/cardiac toxicity	3,990	22	0.6%	NA	Exploratory concomitant-medication burden context.
FAERS reporting	FAERS concomitant-	1-2	Palbociclib	QT/cardiac toxicity	21,374	93	0.4%	NA	Exploratory

Evidence layer	Stratification variable	Stratum	Drug	AESI	Reports	AESI-positive reports	Within-stratum %	Signal summary	Interpretation boundary
context stratification	medication count group								concomitant-medication burden context.
FAERS reporting context stratification	FAERS concomitant-medication count group	1-2	Ribociclib	QT/cardiac toxicity	5,409	244	4.5%	NA	Exploratory concomitant-medication burden context.
FAERS reporting context stratification	FAERS concomitant-medication count group	3-5	Abemaciclib	QT/cardiac toxicity	1,003	13	1.3%	NA	Exploratory concomitant-medication burden context.
FAERS reporting context stratification	FAERS concomitant-medication count group	3-5	Palbociclib	QT/cardiac toxicity	8,448	46	0.5%	NA	Exploratory concomitant-medication burden context.
FAERS reporting context stratification	FAERS concomitant-medication count group	3-5	Ribociclib	QT/cardiac toxicity	2,482	131	5.3%	NA	Exploratory concomitant-medication burden context.
FAERS reporting context stratification	FAERS concomitant-medication count group	>5	Abemaciclib	QT/cardiac toxicity	1,028	8	0.8%	NA	Exploratory concomitant-medication burden context.
FAERS reporting context stratification	FAERS concomitant-medication count group	>5	Palbociclib	QT/cardiac toxicity	11,393	66	0.6%	NA	Exploratory concomitant-medication burden context.
FAERS reporting context stratification	FAERS concomitant-medication count group	>5	Ribociclib	QT/cardiac toxicity	2,279	173	7.6%	NA	Exploratory concomitant-medication burden context.
FAERS reporting context stratification	FAERS concomitant-medication count group	0	Abemaciclib	VTE/thrombotic events	9,309	313	3.4%	NA	Exploratory concomitant-medication burden context.
FAERS reporting context stratification	FAERS concomitant-medication count group	0	Palbociclib	VTE/thrombotic events	39,467	447	1.1%	NA	Exploratory concomitant-medication burden context.
FAERS reporting context stratification	FAERS concomitant-medication count group	0	Ribociclib	VTE/thrombotic events	9,589	134	1.4%	NA	Exploratory concomitant-medication burden context.
FAERS reporting context stratification	FAERS concomitant-medication count group	1-2	Abemaciclib	VTE/thrombotic events	3,990	128	3.2%	NA	Exploratory concomitant-medication burden context.
FAERS reporting context stratification	FAERS concomitant-medication count group	1-2	Palbociclib	VTE/thrombotic events	21,374	425	2.0%	NA	Exploratory concomitant-medication burden context.
FAERS reporting context stratification	FAERS concomitant-medication count group	1-2	Ribociclib	VTE/thrombotic events	5,409	155	2.9%	NA	Exploratory concomitant-medication burden context.

Evidence layer	Stratification variable	Stratum	Drug	AESI	Reports	AESI-positive reports	Within-stratum %	Signal summary	Interpretation boundary
FAERS reporting context stratification	FAERS concomitant-medication count group	3-5	Abemaciclib	VTE/thrombotic events	1,003	48	4.8%	NA	Exploratory concomitant-medication burden context.
FAERS reporting context stratification	FAERS concomitant-medication count group	3-5	Palbociclib	VTE/thrombotic events	8,448	247	2.9%	NA	Exploratory concomitant-medication burden context.
FAERS reporting context stratification	FAERS concomitant-medication count group	3-5	Ribociclib	VTE/thrombotic events	2,482	73	2.9%	NA	Exploratory concomitant-medication burden context.
FAERS reporting context stratification	FAERS concomitant-medication count group	>5	Abemaciclib	VTE/thrombotic events	1,028	26	2.5%	NA	Exploratory concomitant-medication burden context.
FAERS reporting context stratification	FAERS concomitant-medication count group	>5	Palbociclib	VTE/thrombotic events	11,393	313	2.7%	NA	Exploratory concomitant-medication burden context.
FAERS reporting context stratification	FAERS concomitant-medication count group	>5	Ribociclib	VTE/thrombotic events	2,279	112	4.9%	NA	Exploratory concomitant-medication burden context.
FAERS reporting context stratification	FAERS concomitant-medication count group	0	Abemaciclib	ILD/pneumonitis	9,309	183	2.0%	NA	Exploratory concomitant-medication burden context.
FAERS reporting context stratification	FAERS concomitant-medication count group	0	Palbociclib	ILD/pneumonitis	39,467	188	0.5%	NA	Exploratory concomitant-medication burden context.
FAERS reporting context stratification	FAERS concomitant-medication count group	0	Ribociclib	ILD/pneumonitis	9,589	111	1.2%	NA	Exploratory concomitant-medication burden context.
FAERS reporting context stratification	FAERS concomitant-medication count group	1-2	Abemaciclib	ILD/pneumonitis	3,990	146	3.7%	NA	Exploratory concomitant-medication burden context.
FAERS reporting context stratification	FAERS concomitant-medication count group	1-2	Palbociclib	ILD/pneumonitis	21,374	124	0.6%	NA	Exploratory concomitant-medication burden context.
FAERS reporting context stratification	FAERS concomitant-medication count group	1-2	Ribociclib	ILD/pneumonitis	5,409	73	1.3%	NA	Exploratory concomitant-medication burden context.
FAERS reporting context stratification	FAERS concomitant-medication count group	3-5	Abemaciclib	ILD/pneumonitis	1,003	66	6.6%	NA	Exploratory concomitant-medication burden context.
FAERS reporting context stratification	FAERS concomitant-medication count group	3-5	Palbociclib	ILD/pneumonitis	8,448	72	0.9%	NA	Exploratory concomitant-medication burden

Evidence layer	Stratification variable	Stratum	Drug	AESI	Reports	AESI-positive reports	Within-stratum %	Signal summary	Interpretation boundary
									context.
FAERS reporting context stratification	FAERS concomitant-medication count group	3-5	Ribociclib	ILD/pneumonitis	2,482	45	1.8%	NA	Exploratory concomitant-medication burden context.
FAERS reporting context stratification	FAERS concomitant-medication count group	>5	Abemaciclib	ILD/pneumonitis	1,028	59	5.7%	NA	Exploratory concomitant-medication burden context.
FAERS reporting context stratification	FAERS concomitant-medication count group	>5	Palbociclib	ILD/pneumonitis	11,393	102	0.9%	NA	Exploratory concomitant-medication burden context.
FAERS reporting context stratification	FAERS concomitant-medication count group	>5	Ribociclib	ILD/pneumonitis	2,279	60	2.6%	NA	Exploratory concomitant-medication burden context.
FAERS reporting context stratification	FAERS concomitant-medication count group	0	Abemaciclib	Infection/febrile events	9,309	285	3.1%	NA	Exploratory concomitant-medication burden context.
FAERS reporting context stratification	FAERS concomitant-medication count group	0	Palbociclib	Infection/febrile events	39,467	1,429	3.6%	NA	Exploratory concomitant-medication burden context.
FAERS reporting context stratification	FAERS concomitant-medication count group	0	Ribociclib	Infection/febrile events	9,589	354	3.7%	NA	Exploratory concomitant-medication burden context.
FAERS reporting context stratification	FAERS concomitant-medication count group	1-2	Abemaciclib	Infection/febrile events	3,990	175	4.4%	NA	Exploratory concomitant-medication burden context.
FAERS reporting context stratification	FAERS concomitant-medication count group	1-2	Palbociclib	Infection/febrile events	21,374	1,334	6.2%	NA	Exploratory concomitant-medication burden context.
FAERS reporting context stratification	FAERS concomitant-medication count group	1-2	Ribociclib	Infection/febrile events	5,409	432	8.0%	NA	Exploratory concomitant-medication burden context.
FAERS reporting context stratification	FAERS concomitant-medication count group	3-5	Abemaciclib	Infection/febrile events	1,003	87	8.7%	NA	Exploratory concomitant-medication burden context.
FAERS reporting context stratification	FAERS concomitant-medication count group	3-5	Palbociclib	Infection/febrile events	8,448	826	9.8%	NA	Exploratory concomitant-medication burden context.
FAERS reporting context stratification	FAERS concomitant-medication count group	3-5	Ribociclib	Infection/febrile events	2,482	334	13.5%	NA	Exploratory concomitant-medication burden context.
FAERS reporting context stratification	FAERS concomitant-medication count group	>5	Abemaciclib	Infection/febrile events	1,028	89	8.7%	NA	Exploratory concomitant-

Evidence layer	Stratification variable	Stratum	Drug	AESI	Reports	AESI-positive reports	Within-stratum %	Signal summary	Interpretation boundary
									medication burden context.
FAERS reporting context stratification	FAERS concomitant-medication count group	>5	Palbociclib	Infection/febrile events	11,393	1,228	10.8%	NA	Exploratory concomitant-medication burden context.
FAERS reporting context stratification	FAERS concomitant-medication count group	>5	Ribociclib	Infection/febrile events	2,279	405	17.8%	NA	Exploratory concomitant-medication burden context.
FAERS reporting context stratification	FAERS concomitant-medication count group	0	Abemaciclib	Renal/creatinine abnormality	9,309	398	4.3%	NA	Exploratory concomitant-medication burden context.
FAERS reporting context stratification	FAERS concomitant-medication count group	0	Palbociclib	Renal/creatinine abnormality	39,467	549	1.4%	NA	Exploratory concomitant-medication burden context.
FAERS reporting context stratification	FAERS concomitant-medication count group	0	Ribociclib	Renal/creatinine abnormality	9,589	311	3.2%	NA	Exploratory concomitant-medication burden context.
FAERS reporting context stratification	FAERS concomitant-medication count group	1-2	Abemaciclib	Renal/creatinine abnormality	3,990	253	6.3%	NA	Exploratory concomitant-medication burden context.
FAERS reporting context stratification	FAERS concomitant-medication count group	1-2	Palbociclib	Renal/creatinine abnormality	21,374	555	2.6%	NA	Exploratory concomitant-medication burden context.
FAERS reporting context stratification	FAERS concomitant-medication count group	1-2	Ribociclib	Renal/creatinine abnormality	5,409	335	6.2%	NA	Exploratory concomitant-medication burden context.
FAERS reporting context stratification	FAERS concomitant-medication count group	3-5	Abemaciclib	Renal/creatinine abnormality	1,003	100	10.0%	NA	Exploratory concomitant-medication burden context.
FAERS reporting context stratification	FAERS concomitant-medication count group	3-5	Palbociclib	Renal/creatinine abnormality	8,448	281	3.3%	NA	Exploratory concomitant-medication burden context.
FAERS reporting context stratification	FAERS concomitant-medication count group	3-5	Ribociclib	Renal/creatinine abnormality	2,482	207	8.3%	NA	Exploratory concomitant-medication burden context.
FAERS reporting context stratification	FAERS concomitant-medication count group	>5	Abemaciclib	Renal/creatinine abnormality	1,028	117	11.4%	NA	Exploratory concomitant-medication burden context.
FAERS reporting context stratification	FAERS concomitant-medication count group	>5	Palbociclib	Renal/creatinine abnormality	11,393	512	4.5%	NA	Exploratory concomitant-medication burden context.
FAERS reporting	FAERS concomitant-	>5	Ribociclib	Renal/creatinine	2,279	284	12.5%	NA	Exploratory

Evidence layer	Stratification variable	Stratum	Drug	AESI	Reports	AESI-positive reports	Within-stratum %	Signal summary	Interpretation boundary
context stratification	medication count group			abnormality					concomitant-medication burden context.

Table S9. Institutional clinically meaningful AESI by drug.

Institutional findings provide exploratory clinical contextualization from the single-center cohort.

Drug	AESI	Evaluable patients	Clinically meaningful AESI	Proportion (95% CI)	Grade >=3	Dose modification	Hospitalization after AESI	Death after AESI	Median onset days (IQR)
Palbociclib	Hematologic toxicity	44	32	32/44 (72.7%; 57.2% to 85.0%)	30	12	5	1	70 (49-99)
Abemaciclib	Hematologic toxicity	21	14	14/21 (66.7%; 43.0% to 85.4%)	14	5	1	0	69.5 (47-91)
Ribociclib	Hematologic toxicity	6	2	2/6 (33.3%; 4.3% to 77.7%)	2	0	1	0	48 (30-66)
Other CDK4/6 inhibitor	Hematologic toxicity	11	8	8/11 (72.7%; 39.0% to 94.0%)	8	3	2	0	54.5 (44-66)
Palbociclib	Gastrointestinal toxicity	44	11	11/44 (25.0%; 13.2% to 40.3%)	3	4	2	1	87 (58-110)
Abemaciclib	Gastrointestinal toxicity	21	8	8/21 (38.1%; 18.1% to 61.6%)	1	1	0	0	55.5 (35-76)
Ribociclib	Gastrointestinal toxicity	6	3	3/6 (50.0%; 11.8% to 88.2%)	0	1	1	0	67 (48-76)
Other CDK4/6 inhibitor	Gastrointestinal toxicity	11	3	3/11 (27.3%; 6.0% to 61.0%)	0	0	1	0	66 (55-92)
Palbociclib	Hepatobiliary toxicity	44	14	14/44 (31.8%; 18.6% to 47.6%)	0	6	1	2	64.5 (51-102)
Abemaciclib	Hepatobiliary toxicity	21	6	6/21 (28.6%; 11.3% to 52.2%)	0	4	0	0	63.5 (46-66)
Ribociclib	Hepatobiliary toxicity	6	3	3/6 (50.0%; 11.8% to 88.2%)	0	2	0	0	79 (73-85)
Other CDK4/6 inhibitor	Hepatobiliary toxicity	11	2	2/11 (18.2%; 2.3% to 51.8%)	0	0	0	0	71 (48-94)
Palbociclib	QT/cardiac toxicity	44	1	1/44 (2.3%; 0.1% to 12.0%)	0	0	0	0	110 (110-110)
Abemaciclib	QT/cardiac toxicity	21	2	2/21 (9.5%; 1.2% to 30.4%)	0	2	0	0	54 (47-61)
Ribociclib	QT/cardiac toxicity	6	0	0/6 (0.0%; 0.0% to 45.9%)	0	0	0	0	()
Other CDK4/6 inhibitor	QT/cardiac toxicity	11	1	1/11 (9.1%; 0.2% to 41.3%)	0	0	1	0	44 (44-44)
Palbociclib	VTE/thrombotic events	44	0	0/44 (0.0%; 0.0% to 8.0%)	0	0	0	0	()
Abemaciclib	VTE/thrombotic events	21	0	0/21 (0.0%; 0.0% to 16.1%)	0	0	0	0	()
Ribociclib	VTE/thrombotic events	6	0	0/6 (0.0%; 0.0% to 45.9%)	0	0	0	0	()
Other CDK4/6	VTE/thrombotic events	11	0	0/11 (0.0%; 0.0% to	0	0	0	0	()

Drug	AESI	Evaluable patients	Clinically meaningful AESI	Proportion (95% CI)	Grade >=3	Dose modification	Hospitalization after AESI	Death after AESI	Median onset days (IQR)
inhibitor				28.5%)					
Palbociclib	ILD/pneumonitis	44	2	2/44 (4.5%; 0.6% to 15.5%)	0	0	1	0	85.5 (68-103)
Abemaciclib	ILD/pneumonitis	21	2	2/21 (9.5%; 1.2% to 30.4%)	0	0	0	0	105 (99-111)
Ribociclib	ILD/pneumonitis	6	0	0/6 (0.0%; 0.0% to 45.9%)	0	0	0	0	()
Other CDK4/6 inhibitor	ILD/pneumonitis	11	0	0/11 (0.0%; 0.0% to 28.5%)	0	0	0	0	()
Palbociclib	Infection/febrile events	44	3	3/44 (6.8%; 1.4% to 18.7%)	0	1	0	0	84 (66-91)
Abemaciclib	Infection/febrile events	21	0	0/21 (0.0%; 0.0% to 16.1%)	0	0	0	0	()
Ribociclib	Infection/febrile events	6	0	0/6 (0.0%; 0.0% to 45.9%)	0	0	0	0	()
Other CDK4/6 inhibitor	Infection/febrile events	11	1	1/11 (9.1%; 0.2% to 41.3%)	0	0	0	0	44 (44-44)
Palbociclib	Renal/creatinine abnormality	44	9	9/44 (20.5%; 9.8% to 35.3%)	0	0	1	0	51 (35-66)
Abemaciclib	Renal/creatinine abnormality	21	5	5/21 (23.8%; 8.2% to 47.2%)	0	0	0	0	93 (84-97)
Ribociclib	Renal/creatinine abnormality	6	1	1/6 (16.7%; 0.4% to 64.1%)	0	0	0	0	29 (29-29)
Other CDK4/6 inhibitor	Renal/creatinine abnormality	11	2	2/11 (18.2%; 2.3% to 51.8%)	0	0	0	0	40.5 (30-51)

Table S10. Institutional clinical consequences and endocrine-partner context by AESI.

Clinical consequence and endocrine-partner context rows use one shared header.

Evidence layer	Drug	AESI or endocrine partner	Clinical marker	Cases or patients	Denominator	n/N (%)	Interpretation boundary
Clinical consequence by AESI	NA	Hematologic toxicity	Clinically meaningful AESI	56	82	56/82 (68.3%)	Derived from institutional clinical source records for descriptive contextualization.
Clinical consequence by AESI	NA	Hematologic toxicity	Grade >=3	54	82	54/82 (65.9%)	Derived from institutional clinical source records for descriptive contextualization.
Clinical consequence by AESI	NA	Hematologic toxicity	Dose interruption/reduction/discontinuation	20	82	20/82 (24.4%)	Derived from institutional clinical source records for descriptive contextualization.
Clinical consequence by AESI	NA	Hematologic toxicity	All-cause hospitalization after AESI	9	82	9/82 (11.0%)	Derived from institutional clinical source records for descriptive contextualization.
Clinical consequence by	NA	Hematologic toxicity	All-cause death after AESI	1	82	1/82 (1.2%)	Derived from institutional

Evidence layer	Drug	AESI or endocrine partner	Clinical marker	Cases or patients	Denominator	n/N (%)	Interpretation boundary
AESI							clinical source records for descriptive contextualization.
Clinical consequence by AESI	NA	Gastrointestinal toxicity	Clinically meaningful AESI	25	82	25/82 (30.5%)	Derived from institutional clinical source records for descriptive contextualization.
Clinical consequence by AESI	NA	Gastrointestinal toxicity	Grade >=3	4	82	4/82 (4.9%)	Derived from institutional clinical source records for descriptive contextualization.
Clinical consequence by AESI	NA	Gastrointestinal toxicity	Dose interruption/reduction/discontinuation	6	82	6/82 (7.3%)	Derived from institutional clinical source records for descriptive contextualization.
Clinical consequence by AESI	NA	Gastrointestinal toxicity	All-cause hospitalization after AESI	4	82	4/82 (4.9%)	Derived from institutional clinical source records for descriptive contextualization.
Clinical consequence by AESI	NA	Gastrointestinal toxicity	All-cause death after AESI	1	82	1/82 (1.2%)	Derived from institutional clinical source records for descriptive contextualization.
Clinical consequence by AESI	NA	Hepatobiliary toxicity	Clinically meaningful AESI	25	82	25/82 (30.5%)	Derived from institutional clinical source records for descriptive contextualization.
Clinical consequence by AESI	NA	Hepatobiliary toxicity	Grade >=3	0	82	0/82 (0.0%)	Derived from institutional clinical source records for descriptive contextualization.
Clinical consequence by AESI	NA	Hepatobiliary toxicity	Dose interruption/reduction/discontinuation	12	82	12/82 (14.6%)	Derived from institutional clinical source records for descriptive contextualization.
Clinical consequence by AESI	NA	Hepatobiliary toxicity	All-cause hospitalization after AESI	1	82	1/82 (1.2%)	Derived from institutional clinical source records for descriptive contextualization.
Clinical consequence by AESI	NA	Hepatobiliary toxicity	All-cause death after AESI	2	82	2/82 (2.4%)	Derived from institutional clinical source records for descriptive contextualization.
Clinical consequence by AESI	NA	QT/cardiac toxicity	Clinically meaningful AESI	4	82	4/82 (4.9%)	Derived from institutional clinical source records for descriptive contextualization.
Clinical consequence by AESI	NA	QT/cardiac toxicity	Grade >=3	0	82	0/82 (0.0%)	Derived from institutional clinical source records for descriptive contextualization.
Clinical consequence by	NA	QT/cardiac toxicity	Dose	2	82	2/82 (2.4%)	Derived from institutional

Evidence layer	Drug	AESI or endocrine partner	Clinical marker	Cases or patients	Denominator	n/N (%)	Interpretation boundary
AESI			interruption/reduction/discontinuation				clinical source records for descriptive contextualization.
Clinical consequence by AESI	NA	QT/cardiac toxicity	All-cause hospitalization after AESI	1	82	1/82 (1.2%)	Derived from institutional clinical source records for descriptive contextualization.
Clinical consequence by AESI	NA	QT/cardiac toxicity	All-cause death after AESI	0	82	0/82 (0.0%)	Derived from institutional clinical source records for descriptive contextualization.
Clinical consequence by AESI	NA	VTE/thrombotic events	Clinically meaningful AESI	0	82	0/82 (0.0%)	Derived from institutional clinical source records for descriptive contextualization.
Clinical consequence by AESI	NA	VTE/thrombotic events	Grade >=3	0	82	0/82 (0.0%)	Derived from institutional clinical source records for descriptive contextualization.
Clinical consequence by AESI	NA	VTE/thrombotic events	Dose interruption/reduction/discontinuation	0	82	0/82 (0.0%)	Derived from institutional clinical source records for descriptive contextualization.
Clinical consequence by AESI	NA	VTE/thrombotic events	All-cause hospitalization after AESI	0	82	0/82 (0.0%)	Derived from institutional clinical source records for descriptive contextualization.
Clinical consequence by AESI	NA	VTE/thrombotic events	All-cause death after AESI	0	82	0/82 (0.0%)	Derived from institutional clinical source records for descriptive contextualization.
Clinical consequence by AESI	NA	ILD/pneumonitis	Clinically meaningful AESI	4	82	4/82 (4.9%)	Derived from institutional clinical source records for descriptive contextualization.
Clinical consequence by AESI	NA	ILD/pneumonitis	Grade >=3	0	82	0/82 (0.0%)	Derived from institutional clinical source records for descriptive contextualization.
Clinical consequence by AESI	NA	ILD/pneumonitis	Dose interruption/reduction/discontinuation	0	82	0/82 (0.0%)	Derived from institutional clinical source records for descriptive contextualization.
Clinical consequence by AESI	NA	ILD/pneumonitis	All-cause hospitalization after AESI	1	82	1/82 (1.2%)	Derived from institutional clinical source records for descriptive contextualization.
Clinical consequence by AESI	NA	ILD/pneumonitis	All-cause death after AESI	0	82	0/82 (0.0%)	Derived from institutional clinical source records for descriptive contextualization.
Clinical consequence by	NA	Infection/febrile events	Clinically meaningful AESI	4	82	4/82 (4.9%)	Derived from institutional

Evidence layer	Drug	AESI or endocrine partner	Clinical marker	Cases or patients	Denominator	n/N (%)	Interpretation boundary
AESI							clinical source records for descriptive contextualization.
Clinical consequence by AESI	NA	Infection/febrile events	Grade >=3	0	82	0/82 (0.0%)	Derived from institutional clinical source records for descriptive contextualization.
Clinical consequence by AESI	NA	Infection/febrile events	Dose interruption/reduction/discontinuation	1	82	1/82 (1.2%)	Derived from institutional clinical source records for descriptive contextualization.
Clinical consequence by AESI	NA	Infection/febrile events	All-cause hospitalization after AESI	0	82	0/82 (0.0%)	Derived from institutional clinical source records for descriptive contextualization.
Clinical consequence by AESI	NA	Infection/febrile events	All-cause death after AESI	0	82	0/82 (0.0%)	Derived from institutional clinical source records for descriptive contextualization.
Clinical consequence by AESI	NA	Renal/creatinine abnormality	Clinically meaningful AESI	17	82	17/82 (20.7%)	Derived from institutional clinical source records for descriptive contextualization.
Clinical consequence by AESI	NA	Renal/creatinine abnormality	Grade >=3	0	82	0/82 (0.0%)	Derived from institutional clinical source records for descriptive contextualization.
Clinical consequence by AESI	NA	Renal/creatinine abnormality	Dose interruption/reduction/discontinuation	0	82	0/82 (0.0%)	Derived from institutional clinical source records for descriptive contextualization.
Clinical consequence by AESI	NA	Renal/creatinine abnormality	All-cause hospitalization after AESI	1	82	1/82 (1.2%)	Derived from institutional clinical source records for descriptive contextualization.
Clinical consequence by AESI	NA	Renal/creatinine abnormality	All-cause death after AESI	0	82	0/82 (0.0%)	Derived from institutional clinical source records for descriptive contextualization.
Endocrine partner by drug	Abemaciclib	依西美坦(Exemestane)	Patients with any AESI	4	4	4/4 (100.0%)	Single-center descriptive context for practice-context review.
Endocrine partner by drug	Abemaciclib	来曲唑(Letrozole)	Patients with any AESI	5	6	5/6 (83.3%)	Single-center descriptive context for practice-context review.
Endocrine partner by drug	Abemaciclib	氟维司群(Fulvestrant)	Patients with any AESI	4	5	4/5 (80.0%)	Single-center descriptive context for practice-context review.
Endocrine partner by drug	Abemaciclib	阿那曲唑(Anastrozole)	Patients with any AESI	5	6	5/6 (83.3%)	Single-center descriptive context for practice-context review.

Evidence layer	Drug	AESI or endocrine partner	Clinical marker	Cases or patients	Denominator	n/N (%)	Interpretation boundary
Endocrine partner by drug	Other CDK4/6 inhibitor	依西美坦(Exemestane)	Patients with any AESI	3	3	3/3 (100.0%)	Single-center descriptive context for practice-context review.
Endocrine partner by drug	Other CDK4/6 inhibitor	来曲唑(Letrozole)	Patients with any AESI	1	1	1/1 (100.0%)	Single-center descriptive context for practice-context review.
Endocrine partner by drug	Other CDK4/6 inhibitor	氟维司群(Fulvestrant)	Patients with any AESI	4	4	4/4 (100.0%)	Single-center descriptive context for practice-context review.
Endocrine partner by drug	Other CDK4/6 inhibitor	阿那曲唑(Anastrozole)	Patients with any AESI	3	3	3/3 (100.0%)	Single-center descriptive context for practice-context review.
Endocrine partner by drug	Palbociclib	依西美坦(Exemestane)	Patients with any AESI	8	8	8/8 (100.0%)	Single-center descriptive context for practice-context review.
Endocrine partner by drug	Palbociclib	来曲唑(Letrozole)	Patients with any AESI	10	11	10/11 (90.9%)	Single-center descriptive context for practice-context review.
Endocrine partner by drug	Palbociclib	氟维司群(Fulvestrant)	Patients with any AESI	12	13	12/13 (92.3%)	Single-center descriptive context for practice-context review.
Endocrine partner by drug	Palbociclib	阿那曲唑(Anastrozole)	Patients with any AESI	11	12	11/12 (91.7%)	Single-center descriptive context for practice-context review.
Endocrine partner by drug	Ribociclib	依西美坦(Exemestane)	Patients with any AESI	2	2	2/2 (100.0%)	Single-center descriptive context for practice-context review.
Endocrine partner by drug	Ribociclib	来曲唑(Letrozole)	Patients with any AESI	1	1	1/1 (100.0%)	Single-center descriptive context for practice-context review.
Endocrine partner by drug	Ribociclib	氟维司群(Fulvestrant)	Patients with any AESI	3	3	3/3 (100.0%)	Single-center descriptive context for practice-context review.
Endocrine partner overall	All institutional CDK4/6 inhibitors	依西美坦(Exemestane)	Patients with any AESI	17	17	17/17 (100.0%)	Single-center descriptive context for practice-context review.
Endocrine partner overall	All institutional CDK4/6 inhibitors	来曲唑(Letrozole)	Patients with any AESI	17	19	17/19 (89.5%)	Single-center descriptive context for practice-context review.
Endocrine partner overall	All institutional CDK4/6 inhibitors	氟维司群(Fulvestrant)	Patients with any AESI	23	25	23/25 (92.0%)	Single-center descriptive context for practice-context review.
Endocrine partner overall	All institutional CDK4/6 inhibitors	阿那曲唑(Anastrozole)	Patients with any AESI	19	21	19/21 (90.5%)	Single-center descriptive context for practice-context review.

Table S11. FAERS, ML, and institutional concordance matrix.

Concordance categories provide descriptive alignment across evidence layers.

AESI	FAERS top drug	Intraclass OR top drug	ML top drug	Institutional observed top drug	Concordance category
Hematologic toxicity	Palbociclib	Palbociclib	Palbociclib	Palbociclib	fully concordant
Gastrointestinal toxicity	Abemaciclib	Abemaciclib	Abemaciclib	Ribociclib	partially concordant
Hepatobiliary toxicity	Ribociclib	Ribociclib	Ribociclib	Ribociclib	fully concordant
QT/cardiac toxicity	Ribociclib	Ribociclib	Ribociclib	Abemaciclib	partially concordant
VTE/thrombotic events	Abemaciclib	Abemaciclib	Abemaciclib	Not observed	FAERS/ML signal not observed institutionally
ILD/pneumonitis	Abemaciclib	Abemaciclib	Abemaciclib	Abemaciclib	fully concordant
Infection/febrile events	Abemaciclib	Ribociclib	Ribociclib	Other CDK4/6 inhibitor	institutionally observed but not FAERS/ML-prioritised
Renal/creatinine abnormality	Ribociclib	Abemaciclib	Abemaciclib	Abemaciclib	partially concordant

Table S12. Clinical data derivation, recoding rules, and variable availability.

Clinical recoding rules and variable-availability rows use one shared header.

Evidence layer	Dataset or item	Variable domain or derivation item	Status or rule	Columns identified	Rationale or boundary
Clinical derivation rule	Clinical cohort framing	NA	Institutional analysis was framed as a single-center retrospective cohort of 82 patients with documented CDK4/6 inhibitor exposure.	NA	Aligns the institutional component with clinical contextualization.
Clinical derivation rule	Treatment-emergent AESI	NA	AESI required new onset or worsening after CDK4/6 inhibitor initiation plus laboratory abnormality, clinical documentation, dose modification, hospitalization, or death.	NA	Prevents test availability from being counted as an event.
Clinical derivation rule	Grade 3 or higher AESI	NA	Patient-level any grade 3 or higher AESI was separated from domain-specific grade 3 or higher consequences.	NA	Maintains consistency between Table 2 and Figure 6.
Clinical derivation rule	Hospitalization and death	NA	Hospitalization and death were retained as all-cause outcomes after AESI when source records supported all-cause outcome classification.	NA	Documents clinical-consequence boundaries.
Variable availability	FAERS report-level ML dataset	sex	available	sex_group	Structured FAERS-derived demographic group; counts checked against total reports.
Variable availability	FAERS report-level ML dataset	age	available	age_group	Age is available as a grouped FAERS demographic variable.
Variable availability	FAERS report-level ML dataset	report year/time split	available	report_year; split	Used for temporal split and verification.
Variable availability	FAERS report-level ML dataset	reporter geography/type	available	reporter_region; reporter_type	Reporting-context variables that may reflect reporting bias.
Variable availability	FAERS report-level ML dataset	breast-cancer indication grouping	available	indication_group	Derived indication grouping from FAERS indication text.
Variable availability	FAERS report-level ML dataset	endocrine therapy record	available_limited	endocrine_group	FAERS concomitant drug record at report level.
Variable availability	FAERS report-level ML dataset	concomitant medication count	available_limited	concomitant_count_group	Report-level concomitant count for reporting-context review.

Evidence layer	Dataset or item	Variable domain or derivation item	Status or rule	Columns identified	Rationale or boundary
Variable availability	FAERS report-level ML dataset	time-to-onset	available_limited	TTO_group; TTO_days	Only available when valid event/start dates exist; TTO availability checked separately.
Variable availability	FAERS report-level ML dataset	tumor stage	not_available	NA	Not a reliable structured FAERS field in the submitted analysis dataset.
Variable availability	FAERS report-level ML dataset	comorbidity	not_available	NA	Not available as validated patient-level clinical comorbidity.
Variable availability	FAERS report-level ML dataset	line of therapy/baseline risk	not_available	NA	Not available as structured verified treatment-line or baseline-risk variable.
Variable availability	Institutional derived patient table	drug and dates	available	drug; cdk46_start_date; cdk46_end_date; diagnosis_date	Derived patient-level treatment/date fields.
Variable availability	Institutional derived patient table	baseline and worst labs	available	WBC_baseline; WBC_worst; ANC_baseline; ANC_worst; PLT_baseline; PLT_worst; Hb_baseline; Hb_worst; ALT_baseline; ALT_worst	Lab-derived toxicity context; see source columns for WBC/ANC/PLT/Hb/liver/renal markers.
Variable availability	Institutional derived patient table	CTCAE grade and onset	available	CTCAE_grade_numeric; onset_days	Patient-level clinical contextualization variables.
Variable availability	Institutional derived patient table	clinical consequence	available_limited	dose_modified_any; all_cause_hospitalization_after_AESI; all_cause_death_after_AESI	Exploratory clinical contextualization for descriptive practice-context review.
Variable availability	Institutional derived patient table	tumor stage/comorbidity/line therapy	not_in_derived_table	NA	Not present in the derived patient-level CSV used for Figure 6 source-data summary.
Variable availability	Clinical extraction/baseline workbook	age/sex/BMI	available	年龄; 性别; BMI	Clinical extraction workbook fields; some values are inferred/filled.
Variable availability	Clinical extraction/baseline workbook	tumor stage	available	临床病理分期	Non-missing rows: 82/82; includes extracted/inferred clinical information.
Variable availability	Clinical extraction/baseline workbook	comorbidity	available	基础合并症	Non-missing rows: 82/82; use descriptively after verification.
Variable availability	Clinical extraction/baseline workbook	metastasis site/subtype/menopausal status	available	转移灶部位; 分子分型; 绝经状态	Available in clinical extraction workbook; suitability depends on manual verification.
Variable availability	Clinical extraction/baseline workbook	endocrine/concomitant treatment	available	联合内分泌治疗; 其它合并用药	Available for institutional contextualization.
Variable availability	Clinical extraction/baseline workbook	line of therapy	not_available_or_not_structured	NA	No dedicated structured line-of-therapy column identified in the current extraction workbook.
Variable availability	Clinical extraction/baseline workbook	PFS/OS follow-up fields	available_limited	PFS(个月); PFS删失状态(1=事件); OS(个月); OS删失状态(1=事件)	Available but belongs to clinical outcome/PFS follow-up summary, not primary FAERS signal model.

Evidence layer	Dataset or item	Variable domain or derivation item	Status or rule	Columns identified	Rationale or boundary
Variable availability	FAERS report-level ML dataset	prior chemotherapy/prior treatment history	not_available	NA	Not available as a structured verified prior-treatment field in FAERS; not used for stratified FAERS modeling.
Variable availability	Clinical extraction/baseline workbook	prior chemotherapy/prior treatment history	not_available_or_not_structured	NA	No dedicated structured prior-chemotherapy or prior-treatment-history column identified in the current extraction workbook.

Table S13. Representative PT terms selected for the main PT signal panel.

Terms were selected after clinical review for relevance, AESI coverage, report count, disproportionality consistency, and review priority.

Drug	AESI	Preferred term label	Preferred term, English	System organ class	Report n	ROR (95% CI)	Death reports (%)	Serious reports (%)	Positive algorithms	Priority score	Review or inclusion note
Abemaciclib	Hematologic toxicity	Neutropenic Sepsis	NEUTROPENIC SEPSIS	INFECTIONS AND INFESTATIONS	12	2.37 (1.35 to 4.18)	5 (41.7%)	8 (66.7%)	3	5	selected for representative PT signal panel
Abemaciclib	ILD/pneumonitis	Pulmonary Fibrosis	PULMONARY FIBROSIS	RESPIRATORY, THORACIC AND MEDIASTINAL DISORDERS	39	2.93 (2.14 to 4.02)	5 (12.8%)	36 (92.3%)	4	4	selected for representative PT signal panel
Abemaciclib	VTE/thrombotic events	Portal Vein Thrombosis	PORTAL VEIN THROMBOSIS	HEPATOBILIARY DISORDERS	7	3.25 (1.55 to 6.82)	3 (42.9%)	7 (100.0%)	3	4	selected for representative PT signal panel
Abemaciclib	Hepatobiliary toxicity	Hepatic Failure	HEPATIC FAILURE	HEPATOBILIARY DISORDERS	67	3.47 (2.73 to 4.41)	33 (49.3%)	61 (91.0%)	4	5	selected for representative PT signal panel
Abemaciclib	Hepatobiliary toxicity	Drug-Induced Liver Injury	DRUG-INDUCED LIVER INJURY	HEPATOBILIARY DISORDERS	101	3.85 (3.16 to 4.68)	5 (5.0%)	97 (96.0%)	4	4	selected for representative PT signal panel
Abemaciclib	Hematologic toxicity	White Blood Cell Count Decreased	WHITE BLOOD CELL COUNT DECREASED	INVESTIGATIONS	630	7.76 (7.17 to 8.4)	11 (1.7%)	236 (37.5%)	4	3	selected for representative PT signal panel
Abemaciclib	Gastrointestinal toxicity	Diarrhoea	DIARRHOEA	GASTROINTESTINAL DISORDERS	5,735	12.82 (12.47 to 13.18)	82 (1.4%)	1,440 (25.1%)	4	4	selected for representative PT signal panel

Drug	AESI	Preferred term label	Preferred term, English	System organ class	Report n	ROR (95% CI)	Death reports (%)	Serious reports (%)	Positive algorithms	Priority score	Review or inclusion note
Palbociclib	Hepatobiliary toxicity	Hepatic Failure	HEPATIC FAILURE	HEPATOBILIARY DISORDERS	201	1.25 (1.09 to 1.43)	81 (40.3%)	120 (59.7%)	2	4	selected for representative PT signal panel
Palbociclib	ILD/pneumonitis	Pneumonitis	PNEUMONITIS	RESPIRATORY, THORACIC AND MEDIASTINAL DISORDERS	370	2.1 (1.9 to 2.33)	31 (8.4%)	190 (51.4%)	3	3	selected for representative PT signal panel
Palbociclib	Gastrointestinal toxicity	Nausea	NAUSEA	GASTROINTESTINAL DISORDERS	10,911	2.28 (2.24 to 2.33)	291 (2.7%)	3,076 (28.2%)	4	3	selected for representative PT signal panel
Palbociclib	Hematologic toxicity	Pancytopenia	PANCYTOPENIA	BLOOD AND LYMPHATIC SYSTEM DISORDERS	760	2.43 (2.26 to 2.61)	59 (7.8%)	468 (61.6%)	4	4	selected for representative PT signal panel
Palbociclib	VTE/thrombotic events	Pulmonary Thrombosis	PULMONARY THROMBOSIS	RESPIRATORY, THORACIC AND MEDIASTINAL DISORDERS	249	3.58 (3.15 to 4.06)	6 (2.4%)	158 (63.5%)	4	3	selected for representative PT signal panel
Palbociclib	Hematologic toxicity	Neutropenia	NEUTROPENIA	BLOOD AND LYMPHATIC SYSTEM DISORDERS	6,823	8.47 (8.27 to 8.68)	209 (3.1%)	4,152 (60.9%)	4	4	selected for representative PT signal panel
Palbociclib	Hematologic toxicity	White Blood Cell Count Decreased	WHITE BLOOD CELL COUNT DECREASED	INVESTIGATIONS	15,875	27.68 (27.21 to 28.16)	413 (2.6%)	4,794 (30.2%)	4	3	selected for representative PT signal panel
Ribociclib	Hematologic toxicity	Pancytopenia	PANCYTOPENIA	BLOOD AND LYMPHATIC SYSTEM DISORDERS	183	2.1 (1.81 to 2.42)	23 (12.6%)	139 (76.0%)	3	4	selected for representative PT signal panel
Ribociclib	Hematologic toxicity	Neutropenic Sepsis	NEUTROPENIC SEPSIS	INFECTIONS AND INFESTATIONS	26	2.23 (1.52 to 3.28)	6 (23.1%)	18 (69.2%)	3	4	selected for representative PT signal panel
Ribociclib	Hepatobiliary toxicity	Autoimmune Hepatitis	AUTOIMMUNE HEPATITIS	HEPATOBILIARY DISORDERS	32	2.68 (1.89 to 3.79)	1 (3.1%)	28 (87.5%)	3	4	selected for representative PT signal panel
Ribociclib	Hepatobiliary toxicity	Hepatic Failure	HEPATIC FAILURE	HEPATOBILIARY DISORDERS	122	2.74 (2.29 to 3.27)	63 (51.6%)	99 (81.1%)	4	6	selected for representative PT signal panel
Ribociclib	Hematologic toxicity	Neutropenia	NEUTROPENIA	BLOOD AND LYMPHATIC SYSTEM DISORDERS	1,950	8.48 (8.1 to 8.87)	99 (5.1%)	1,562 (80.1%)	4	4	selected for representative PT signal panel
Ribociclib	QT/cardiac toxicity	Electrocardiogram Qt Prolonged	ELECTROCARDIOGRAM QT PROLONGED	INVESTIGATIONS	669	10.82 (10.02 to 11.68)	21 (3.1%)	523 (78.2%)	4	3	selected for representative PT signal panel

Table S14. PT terms excluded or requiring manual review for figure construction.

Rows document preferred terms excluded from the main visual panel or retained only for review context.

Drug	AESI	Preferred term label	Preferred term, English	System organ class	Report n	ROR (95% CI)	Death reports (%)	Serious reports (%)	Positive algorithms	Priority score	Review or inclusion note
Abemaciclib	VTE/thrombotic events	Pulmonary Tumour Thrombotic Microangiopathy	PULMONARY TUMOUR THROMBOTIC MICROANGIOPATHY	RESPIRATORY, THORACIC AND MEDIASTINAL DISORDERS	3	41.74 (13.24 to 131.59)	1 (33.3%)	2 (66.7%)	4	4	excluded from main figure panel or retained only for review context
Ribociclib	Renal/creatinine abnormality	Metastases To Kidney	METASTASES TO KIDNEY	NEOPLASMS BENIGN, MALIGNANT AND	12	12.87 (7.26 to 22.8)	3 (25.0%)	12 (100.0%)	4	4	excluded from main figure panel or retained only for review context

Drug	AESI	Preferred term label	Preferred term, English	System organ class	Report n	ROR (95% CI)	Death reports (%)	Serious reports (%)	Positive algorithms	Priority score	Review or inclusion note
				UNSPECIFIED (INCL CYSTS AND POLYPS)							
Ribociclib	Hepatobiliary toxicity	Metastases To Liver	METASTASES TO LIVER	NEOPLASMS BENIGN, MALIGNANT AND UNSPECIFIED (INCL CYSTS AND POLYPS)	546	18.47 (16.96 to 20.11)	126 (23.1%)	472 (86.4%)	4	3	excluded from main figure panel or retained only for review context

Table S15. Preferred-term cleaning, clinical review, sensitivity analysis, and death/fatal outcome-handling analysis.

PT review, cleaning sensitivity, and death/outcome summary rows were converted into a single long table with one shared header.

Evidence layer	Drug or source	AESI	Item or scenario	Signal or count summary	Death/fatal or serious information	Top PTs or source relevance	Interpretation note
Clinical-review PT	Palbociclib	Gastrointestinal toxicity	Stomatitis Necrotising	STOMATITIS NECROTISING; SOC=GASTROINTESTINAL DISORDERS; report n=8; ROR=9.07 (4.45 to 18.48); positive algorithms=4; priority score=3	death reports=0 (0.0%); serious reports=5 (62.5%)	NA	Retained for clinical review before high-level visual interpretation.
Clinical-review PT	Abemaciclib	Infection/febrile events	Atypical Mycobacterial Infection	ATYPICAL MYCOBACTERIAL INFECTION; SOC=INFECTIONS AND INFESTATIONS; report n=5; ROR=5.17 (2.15 to 12.44); positive algorithms=4; priority score=4	death reports=2 (40.0%); serious reports=3 (60.0%)	NA	Retained for clinical review before high-level visual interpretation.
Clinical-review PT	Ribociclib	Hepatobiliary toxicity	Hepatic Infection	HEPATIC INFECTION; SOC=INFECTIONS AND INFESTATIONS; report n=15; ROR=6.59 (3.96 to 10.96); positive algorithms=4; priority score=4	death reports=4 (26.7%); serious reports=15 (100.0%)	NA	Retained for clinical review before high-level visual interpretation.
Clinical-review PT	Ribociclib	Gastrointestinal toxicity	Faecal Vomiting	FAECAL VOMITING; SOC=GASTROINTESTINAL DISORDERS; report n=4; ROR=4.17 (1.56 to 11.15); positive algorithms=3; priority score=4	death reports=2 (50.0%); serious reports=4 (100.0%)	NA	Retained for clinical review before high-level visual interpretation.
Clinical-review PT	Ribociclib	Hepatobiliary toxicity	Coma Hepatic	COMA HEPATIC; SOC=NERVOUS SYSTEM DISORDERS; report n=3; ROR=3.69 (1.19 to 11.49); positive algorithms=2; priority score=4	death reports=3 (100.0%); serious reports=3 (100.0%)	NA	Retained for clinical review before high-level visual interpretation.
PT cleaning sensitivity	Abemaciclib	NA	unfiltered_positive_PT	eligible PT rows=272	death/fatal rows=1; top 10 contains death/fatal=Yes	HEPATIC FAILURE; NEUTROPENIC SEPSIS; DEATH; DIARRHOEA; MALIGNANT NEOPLASM PROGRESSION; DRUG-INDUCED LIVER INJURY; PULMONARY FIBROSIS; DISEASE COMPLICATION; LYMPHANGIOSIS CARCINOMATOSA; PSEUDOCIRRHOSIS	Sensitivity summary for preferred-term cleaning and reviewer-requested death/outcome handling.
PT cleaning sensitivity	Palbociclib	NA	unfiltered_positive_PT	eligible PT rows=576	death/fatal rows=1; top 10 contains death/fatal=Yes	DEATH; NEUTROPENIA; BONE MARROW FAILURE;	Sensitivity summary for preferred-term cleaning and

Evidence layer	Drug or source	AESI	Item or scenario	Signal or count summary	Death/fatal or serious information	Top PTs or source relevance	Interpretation note
						PANCYTOPENIA; SURFACTANT PROTEIN INCREASED; PARANEOPLASTIC SYNDROME; HEPATIC FAILURE; FATIGUE; WHITE BLOOD CELL COUNT DECREASED; NEOPLASM PROGRESSION	reviewer-requested death/outcome handling.
PT cleaning sensitivity	Ribociclib	NA	unfiltered_positive_PT	eligible PT rows=819	death/fatal rows=3; top 10 contains death/fatal=Yes	HEPATIC FAILURE; MALIGNANT NEOPLASM PROGRESSION; HYDROTHORAX; BRAIN STEM GLIOMA; DEATH; NEUTROPENIA; BREAST CANCER METASTATIC; BREAST CANCER; METASTASES TO CENTRAL NERVOUS SYSTEM; ASCITES	Sensitivity summary for preferred-term cleaning and reviewer-requested death/outcome handling.
PT cleaning sensitivity	Abemaciclib	NA	old_main_cleaning	eligible PT rows=260	death/fatal rows=1; top 10 contains death/fatal=Yes	HEPATIC FAILURE; NEUTROPENIC SEPSIS; DEATH; DIARRHOEA; DRUG-INDUCED LIVER INJURY; PULMONARY FIBROSIS; DISEASE COMPLICATION; LYMPHANGIOSIS CARCINOMATOSA; PSEUDOCIRRHOSIS; MALIGNANT PLEURAL EFFUSION	Sensitivity summary for preferred-term cleaning and reviewer-requested death/outcome handling.
PT cleaning sensitivity	Palbociclib	NA	old_main_cleaning	eligible PT rows=561	death/fatal rows=1; top 10 contains death/fatal=Yes	DEATH; NEUTROPENIA; BONE MARROW FAILURE; PANCYTOPENIA; SURFACTANT PROTEIN INCREASED; PARANEOPLASTIC SYNDROME; HEPATIC FAILURE; FATIGUE; WHITE BLOOD CELL COUNT DECREASED; NAUSEA	Sensitivity summary for preferred-term cleaning and reviewer-requested death/outcome handling.
PT cleaning sensitivity	Ribociclib	NA	old_main_cleaning	eligible PT rows=765	death/fatal rows=3; top 10 contains death/fatal=Yes	HEPATIC FAILURE; HYDROTHORAX; BRAIN STEM GLIOMA; DEATH; NEUTROPENIA; ASCITES; METASTATIC NEOPLASM; GRANULOCYTOPENIA; ORGAN FAILURE; BONE MARROW INFILTRATION	Sensitivity summary for preferred-term cleaning and reviewer-requested death/outcome handling.

Evidence layer	Drug or source	AESI	Item or scenario	Signal or count summary	Death/fatal or serious information	Top PTs or source relevance	Interpretation note
PT cleaning sensitivity	Abemaciclib	NA	reviewer_death_outcome_cleaning	eligible PT rows=259	death/fatal rows=0; top 10 contains death/fatal=No	HEPATIC FAILURE; NEUTROPENIC SEPSIS; DIARRHOEA; DRUG-INDUCED LIVER INJURY; PULMONARY FIBROSIS; DISEASE COMPLICATION; LYMPHANGIOSIS CARCINOMATOSA; PSEUDOCIRRHOSIS; MALIGNANT PLEURAL EFFUSION; PNEUMOMEDIASTINUM	Sensitivity summary for preferred-term cleaning and reviewer-requested death/outcome handling.
PT cleaning sensitivity	Palbociclib	NA	reviewer_death_outcome_cleaning	eligible PT rows=560	death/fatal rows=0; top 10 contains death/fatal=No	NEUTROPENIA; BONE MARROW FAILURE; PANCYTOPENIA; SURFACTANT PROTEIN INCREASED; PARANEOPLASTIC SYNDROME; HEPATIC FAILURE; FATIGUE; WHITE BLOOD CELL COUNT DECREASED; NAUSEA; ALOPECIA	Sensitivity summary for preferred-term cleaning and reviewer-requested death/outcome handling.
PT cleaning sensitivity	Ribociclib	NA	reviewer_death_outcome_cleaning	eligible PT rows=762	death/fatal rows=0; top 10 contains death/fatal=No	HEPATIC FAILURE; HYDROTHORAX; BRAIN STEM GLIOMA; NEUTROPENIA; ASCITES; METASTATIC NEOPLASM; GRANULOCYTOPENIA; ORGAN FAILURE; BONE MARROW INFILTRATION; ONCOLOGIC COMPLICATION	Sensitivity summary for preferred-term cleaning and reviewer-requested death/outcome handling.
Death/fatal outcome-handling summary	Death/fatal PT analysis	NA	Screened and retained/deleted death-like PT rows	screened=42; retained descriptive=20; deleted=22	Generic death/fatal labels handled as outcome metadata outside the primary PT signal panel.	BRAIN DEATH; CELL DEATH; DEATH OF RELATIVE; DROWNING; FEAR OF DEATH; NEAR DEATH EXPERIENCE	Non-specific or clinically ambiguous death-like PTs are summarized as death/outcome exclusion metadata.
Death/outcome source reference	FDA AEMS/FAERS latest quarterly data files	NA	FAERS death/outcome handling source	https://www.fda.gov/drugs/fda-adverse-event-monitoring-system-aems/fda-adverse-event-monitoring-system-aems-latest-quarterly-data-files	NA	FAERS quarterly files separate reaction information from patient outcome information.	Source used to document separation of reaction terms from report-level outcomes.
Death/outcome source reference	FDA FAERS Public Dashboard FAQ	NA	FAERS death/outcome handling source	https://fis.fda.gov/extensions/FPD-FAQ/FPD-FAQ.html	NA	Patient outcomes are report-level serious outcomes; causality and incidence require denominator-based evidence.	Source used to document separation of reaction terms from report-level outcomes.
Death/outcome source reference	openFDA Drug Adverse Event field reference	NA	FAERS death/outcome handling source	https://open.fda.gov/fields/drugevent_reference.pdf	NA	reactionmeddrapt is a reaction term; seriousnessdeath and patientdeath are separate	Source used to document separation of reaction terms from report-level outcomes.

Evidence layer	Drug or source	AESI	Item or scenario	Signal or count summary	Death/fatal or serious information	Top PTs or source relevance	Interpretation note
						death/outcome fields.	
Death/outcome source reference	openFDA Drug Event API overview	NA	FAERS death/outcome handling source	https://open.fda.gov/apis/drug/event/	NA	FAERS reports can include multiple drugs and multiple reactions; causal assessment requires source-level and denominator-based evidence.	Source used to document separation of reaction terms from report-level outcomes.

Table S16. Supplementary figure map, boundary statements, and Figure 6 source-data summary.

The figure map and Figure 6 source-data summary summary use one shared header.

Evidence layer	Figure or source panel	Main message or checks	PASS checks	Overall status	Boundary
Figure map	Figure 1	Dual-track study workflow linking FAERS and institutional clinical contextualization.	NA	NA	Boundary statements define the evidentiary role of each figure.
Figure map	Figure 2	FAERS AESI landscape and representative preferred-term signatures.	NA	NA	Boundary statements define the evidentiary role of each figure.
Figure map	Figure 3	Intraclass adjusted reporting odds and reported TTO.	NA	NA	Boundary statements define the evidentiary role of each figure.
Figure map	Figure 4	ML temporal validation with AUROC, AUPRC, ROC, and PR curves.	NA	NA	Boundary statements define the evidentiary role of each figure.
Figure map	Figure 5	Model contribution, ML-ROR concordance, and calibration.	NA	NA	Boundary statements define the evidentiary role of each figure.
Figure map	Figure 6	Institutional clinical contextualization.	NA	NA	Boundary statements define the evidentiary role of each figure.
Figure 6 source-data summary	Panel A	32 source-count checks performed	32	PASS	Verified against institutional patient/event-level derived source files; exploratory contextualization only.
Figure 6 source-data summary	Panel B	40 source-count checks performed	40	PASS	Verified against institutional patient/event-level derived source files; exploratory contextualization only.
Figure 6 source-data summary	Panel C	Figure-map source-data and top-drug context verification performed	8 AESI rows checked across FAERS signal, ML priority, and institutional context layers	PASS	Panel C is a descriptive integration map; it does not estimate incidence or causal effects.
